# A Water-Soluble Curcumin Glycoside Exhibits Superior Anti-Colitic Efficacy over Native Curcumin in DSS-Induced Colitis through Anti-Inflammatory Activity and Gut Microbiota Modulation

**DOI:** 10.4014/jmb.2604.04082

**Published:** 2026-08-05

**Authors:** Seung Yeob Yu, Hyebin Park, Dong Jin Yu, Woo Song Lee, Hyung Jae Jeong, Ju Huck Lee

**Affiliations:** 1 Korean Collection for Type Cultures, Biological Resource Center, Korea Research Institute of Bioscience and Biotechnology, Jeongeup 56212, Republic of Korea; 2 University of Science and Technology (UST), Daejeon 34113, Republic of Korea; 3 Bioten Corporation, Jeongeup 56212, Republic of Korea; 4 Functional Biomaterial Research Center, Korea Research Institute of Bioscience and Biotechnology, Jeongeup 56212, Republic of Korea

**Keywords:** Curcumin, Anti-inflammation, Colitis, Gut microbiota

## Abstract

Curcumin is a hydrophobic polyphenol with diverse biological activities; however, its practical application is limited by poor water solubility and low oral bioavailability. In this study, we investigated whether TSP, a previously developed water-soluble curcumin-stevioside glycoside, exerts superior anti-colitic efficacy compared with native curcumin. In LPS-stimulated macrophages, TSP more effectively suppressed IL-6 and TNF-α production than curcumin under aqueous conditions, indicating greater anti-inflammatory activity in a physiologically relevant environment. In a dextran sulfate sodium (DSS)-induced mouse colitis model, oral administration of TSP ameliorated disease severity more effectively than native curcumin administered at an equivalent curcumin dose, as evidenced by reduced body weight loss, preservation of colon length, improved histopathological features, and restoration of intestinal barrier integrity. TSP also was associated with altered gut microbial diversity and composition relative to the DSS group, including enrichment of short-chain fatty acid (SCFA)-associated taxa, particularly *Akkermansia*. Consistent with these compositional changes, functional prediction analysis revealed an increased abundance of microbial genetic pathways associated with SCFA biosynthesis in the TSP-treated group. Collectively, these findings demonstrate that TSP exerts superior anti-colitic effects relative to native curcumin, likely owing to its improved aqueous solubility and the resulting enhancement of curcumin availability under physiological conditions. The protective effects of TSP appear to involve both direct suppression of inflammatory responses and microbiota-mediated support of intestinal barrier homeostasis. These results highlight TSP as a promising curcumin-based candidate for functional food or adjunct therapeutic applications in inflammatory bowel disease.

## Introduction

Inflammatory bowel disease (IBD), including Crohn’s disease and ulcerative colitis, is a chronic relapsing inflammatory disorder of the gastrointestinal tract characterized by abdominal pain, mucosal ulceration, bloody stools, and diarrhea [1]. Although the molecular pathogenesis of IBD has not been fully elucidated, accumulating evidence suggests that it is driven by a complex interplay among gut microbiota dysbiosis, epithelial barrier disruption, and dysregulated mucosal immune responses [2, 3]. Under healthy conditions, the intestinal immune system maintains tolerance toward commensal microbes and dietary antigens; in IBD, however, this homeostatic balance is disrupted, resulting in excessive immune activation against luminal antigens [3]. In particular, compromised barrier integrity facilitates the translocation of microbial-associated molecular patterns (MAMPs), which activate innate immune cells such as macrophages and trigger the overproduction of pro-inflammatory cytokines, including tumor necrosis factor-alpha (TNF-α), interleukin-6 (IL-6), and IL-1β [3, 4]. This persistent inflammatory cascade further aggravates epithelial injury and perpetuates chronic intestinal inflammation [3, 4]. Although conventional therapies, including 5-aminosalicylic acid (5-ASA) and TNF-α inhibitors, are widely used, their long-term clinical utility is often limited by incomplete response, secondary loss of efficacy, and significant adverse effects [3, 5, 6]. Therefore, safer and more effective therapeutic strategies are urgently needed, particularly bioavailable agents capable of simultaneously attenuating intestinal inflammation and restoring gut microbial homeostasis.

Curcumin is a major bioactive polyphenol derived from turmeric (*Curcuma longa*), a medicinal plant belonging to the *Zingiberaceae* family that has long been used in traditional medicine [7]. Structurally, curcumin contains two aromatic rings linked by an α, β-unsaturated diketone moiety, a feature closely associated with its diverse biological activities [7]. Extensive studies have shown that curcumin exerts a broad range of pharmacological effects, including anti-inflammatory, antioxidant, anti-fibrotic, hypolipidemic, and anti-atherosclerotic activities [8-11]. Notably, curcumin has been reported to suppress key inflammatory mediators and signaling pathways, including inducible nitric oxide synthase (iNOS), TNF-α, and nuclear factor-kappa B (NF-κB) [12]. In addition, curcumin exhibits relatively low toxicity, supporting its potential as a safe bioactive compound for therapeutic or nutraceutical applications [13]. Despite these beneficial properties, however, the practical application of curcumin remains limited because of its poor water solubility, chemical instability, and low systemic bioavailability.

To overcome the limitations of native curcumin, various formulation strategies, including nanoparticles, liposomes, and other delivery systems, have been explored to improve its solubility and bioavailability [14-17]. More recently, glycosylation has emerged as a promising approach for enhancing the physicochemical properties of hydrophobic bioactive compounds [18-20]. In this context, Water-Soluble Curcumin Complex (TSP), a previously developed water-soluble curcumin-stevioside glycoside prepared using a microwave-assisted process, has attracted attention as a potential alternative to conventional curcumin formulations [21, 22]. Previous studies have shown that TSP markedly improves the aqueous solubility and stability of curcumin while preserving its biological activity, and that it can exert antioxidant and immunomodulatory effects under biologically relevant conditions [21, 22]. However, whether these physicochemical advantages translate into superior efficacy against colitis and gut microbiota dysbiosis compared with native curcumin remains unclear.

Because IBD is driven by excessive inflammatory responses, epithelial barrier disruption, and gut microbial dysbiosis, we hypothesized that the improved aqueous solubility of TSP would translate into greater biological activity under physiological conditions and, consequently, superior therapeutic efficacy compared with native curcumin in colitis. To test this hypothesis, we first compared the anti-inflammatory activities of TSP and curcumin in LPS-stimulated macrophages. We then employed a DSS-induced mouse colitis model to determine whether oral administration of TSP provides greater protection than native curcumin against intestinal inflammation, barrier dysfunction, and gut microbiota disruption. Collectively, our study identifies TSP as a promising curcumin-based candidate with enhanced practical efficacy for the management of intestinal inflammatory disorders.

## Materials and Methods

### Preparation of TSP

To prepare the water-soluble curcumin complex, 10 g of curcumin and 200 g of stevioside were dispersed in 1.0 L of distilled water in a 2.0 L glass bottle. The resulting suspension was exposed using a microwave-assisted extraction system (MARS; CEM, USA) at 1200 W for 30 min. The processed mixture was then filtered, and the filtrate was freeze-dried to yield the water-soluble curcumin complex powder (TSP) for use in subsequent experiments.

### Quantification of Curcumin in TSP by HPLC

The curcumin content of the TSP powder was quantified using an Agilent 1100 series HPLC system (Agilent Technologies, USA). Chromatographic separation was performed on a ZORBAX SB-C18 column (4.6 × 150 mm, 5 μm) maintained at 30°C. Isocratic elution was carried out using a mobile phase consisting of 0.1% trifluoroacetic acid (TFA) in water and 0.1% TFA in acetonitrile (55:45, v/v) at a flow rate of 1.0 mL/min. The injection volume was 10 μL. Curcumin standard solutions were prepared in methanol at concentrations ranging from 0.015 to 1.0 mg/mL to generate a calibration curve. The resulting regression equation was *y* = 30077.03*x* + 20.57 (*r²* = 0.9999). Based on this calibration curve, the curcumin content in the TSP powder was determined to be 18.6 g/kg, indicating that 100 mg of the final TSP formulation contains 1.86 mg of curcumin. Based on the HPLC quantification of curcumin and the initial input ratio (or total recovered mass), the stevioside content in 100 mg of the final TSP powder was estimated to be 71.43 mg.

### *In Vitro* Macrophage Stimulation Assay

RAW 264.7 cells were seeded in 24-well plates at a density of 3 × 10^5^ cells/well and cultured in Dulbecco’s modified Eagle’s medium (DMEM; Welgene, Republic of Korea) supplemented with 10% fetal bovine serum (FBS; Welgene, Republic of Korea) and 1% antibiotic-antimycotic solution (ABS; Welgene, Republic of Korea). Cells were incubated at 37°C in a humidified atmosphere containing 5% CO_2_ for 24 h. After incubation, the medium was replaced with fresh DMEM containing 1% ABS. Cells were then co-treated with lipopolysaccharide (LPS; 500 ng/mL) and the indicated samples for 6 h. TSP was administered at concentrations corresponding to 10 or 20 μM curcumin based on its curcumin content. For comparison, native curcumin was treated at the same final concentrations (10 or 20 μM), and stevioside was administered at amounts equivalent to those contained in the respective TSP preparations. To compare activity under lipophilic and aqueous conditions, samples were dissolved in dimethyl sulfoxide (DMSO) or distilled water (DW), respectively, and sterilized by filtration through a 0.2 μm pore-size filter before use. After treatment, culture supernatants were collected, and the concentrations of IL-6 and TNF-α were measured using ELISA kits (BD, USA) according to the manufacturer’s instructions.

### Mice

Five-week-old male C57BL/6 mice were obtained from KOSA BIO (Republic of Korea). Mice were housed under specific pathogen-free conditions at a density of four animals per cage in a temperature- and humidity-controlled room (23 ± 2°C, 55 ± 10% relative humidity) under a 12 h light/dark cycle, with lights on from 09:00 to 21:00.

### DSS-Induced Colitis Model and Oral Administration of Test Substances

After a 7-day acclimatization period, mice were randomly assigned to five groups: normal control (Con), DSS control (DSS), TSP-treated (TSP), curcumin-treated (Cur), and stevioside-treated (Stv) groups. All test substances, including TSP, curcumin, and stevioside, were suspended in 2% carboxymethyl cellulose (CMC) and administered by oral gavage every other day for 14 days from day 0 to day 14. TSP was administered at 100 mg/kg, which corresponded to 1.86 mg/kg curcumin based on its curcumin content, and curcumin was administered at an equivalent dose of 1.86 mg/kg. Stevioside was administered at 71.43 mg/kg, corresponding to the amount of stevioside present in the TSP preparation. The Con and DSS groups received the vehicle alone.

Acute colitis was induced by providing 2.5% (w/v) dextran sulfate sodium (DSS; molecular weight, 36,000−50,000; MP Biomedicals, USA) in the drinking water for 7 consecutive days from day 7 to day 14 in all groups except the Con group, which received normal drinking water throughout the experiment. During the DSS treatment period, body weight and disease activity index (DAI) were monitored daily. On day 14, mice were euthanized, and colon tissues, serum, and fecal samples were collected and immediately stored at -80°C until further analysis.

### Histopathological Analysis of Colon Tissues

Collected colon tissues were fixed in 10% neutral-buffered formalin and embedded in paraffin. The paraffin blocks were sectioned into 5 μm-thick slices and stained with hematoxylin and eosin (H&E). For histopathological analysis, sections were prepared from the distal colon (1−2 cm proximal to the anus) in all animals, and at least three non-overlapping sections from the same anatomical region of each colon were examined under a light microscope equipped with 4 × and 20 × objective lenses. Histopathological scores were assigned on a scale of 1 to 5 according to the degree of inflammatory cell infiltration, epithelial damage, and disruption of mucosal architecture.

### *In vivo* Intestinal Permeability Assay

Intestinal barrier integrity was evaluated *in vivo* using fluorescein isothiocyanate-conjugated dextran (FITC-dextran; Sigma-Aldrich, USA). Mice were fasted for 12 h and then orally administered FITC-dextran (400 mg/kg) dissolved in phosphate-buffered saline (PBS). Four hours after administration, blood samples were collected, and serum was isolated. To establish a baseline fluorescence level, an additional normal control group without FITC-dextran administration was included in the measurement. The fluorescence intensity of FITC-dextran in serum was measured using a microplate reader at excitation and emission wavelengths of 485 and 538 nm, respectively.

### Fecal Microbiota Analysis by 16S rRNA Gene Sequencing

Microbial DNA was extracted from approximately 200 mg of mouse feces using the QIAamp Fast DNA Stool Mini Kit (Qiagen, Germany) according to the manufacturer’s instructions. The V3-V4 hypervariable region of the bacterial 16S rRNA gene was amplified using the primers 341F (5'-CCTACGGGNGGCWGCAG-3') and 805R (5'-GACTACHVGGTATCTAATCC-3'). Sequencing was carried out by Macrogen (Republic of Korea). Raw reads were processed using DADA2 (v1.18.0) [23], including dereplication, sequence-variant inference, and error correction. Amplicon sequence variants (ASVs) were classified using BLASTN (v2.9.0+) against the NCBI 16S Microbial database [24]. Representative ASV sequences were aligned using MAFFT (v7.474) [25]. Alpha-diversity was analyzed using QIIME (v1.9.0) [26] based on the ACE, Shannon, and Simpson indices. Group differences in alpha-diversity were tested by one-way analysis of variance (ANOVA) followed by Dunnett’s *post-hoc* test when each group was compared with a designated reference group. Beta-diversity was assessed using Bray-Curtis dissimilarity and visualized by principal coordinate analysis (PCoA). Differences in microbial community structure were evaluated using PERMANOVA, and the corresponding pseudo-F statistic, R^2^ value and *P* value were reported.

Differential abundance analysis of microbial taxa was additionally performed using ANCOM-BC with Benjamini–Hochberg false discovery rate correction to account for the compositional nature of 16S rRNA sequencing data. Genus-level raw count data prior to relative-abundance normalization were used as input, and the DSS group was set as the reference group. Functional profiles were predicted from the 16S rRNA gene data using Tax4Fun through MicrobiomeAnalyst [27, 28].

### Statistical Analysis

Statistical analyses for non-microbiota experimental data were performed using GraphPad Prism software (GraphPad Software, USA). Data are presented as mean ± standard error of the mean (SEM). Comparisons among multiple groups were performed using one-way ANOVA followed by Dunnett’s *post-hoc* test. Differences were considered statistically significant at *P* < 0.05. Significance levels are indicated as follows: * *P* < 0.05, ** *P* < 0.01, *** *P* < 0.001, and **** *P* < 0.0001; ns, not significant.

## Results

### TSP Shows Superior Anti-Inflammatory Activity under Aqueous Conditions

**Fig. 1:**
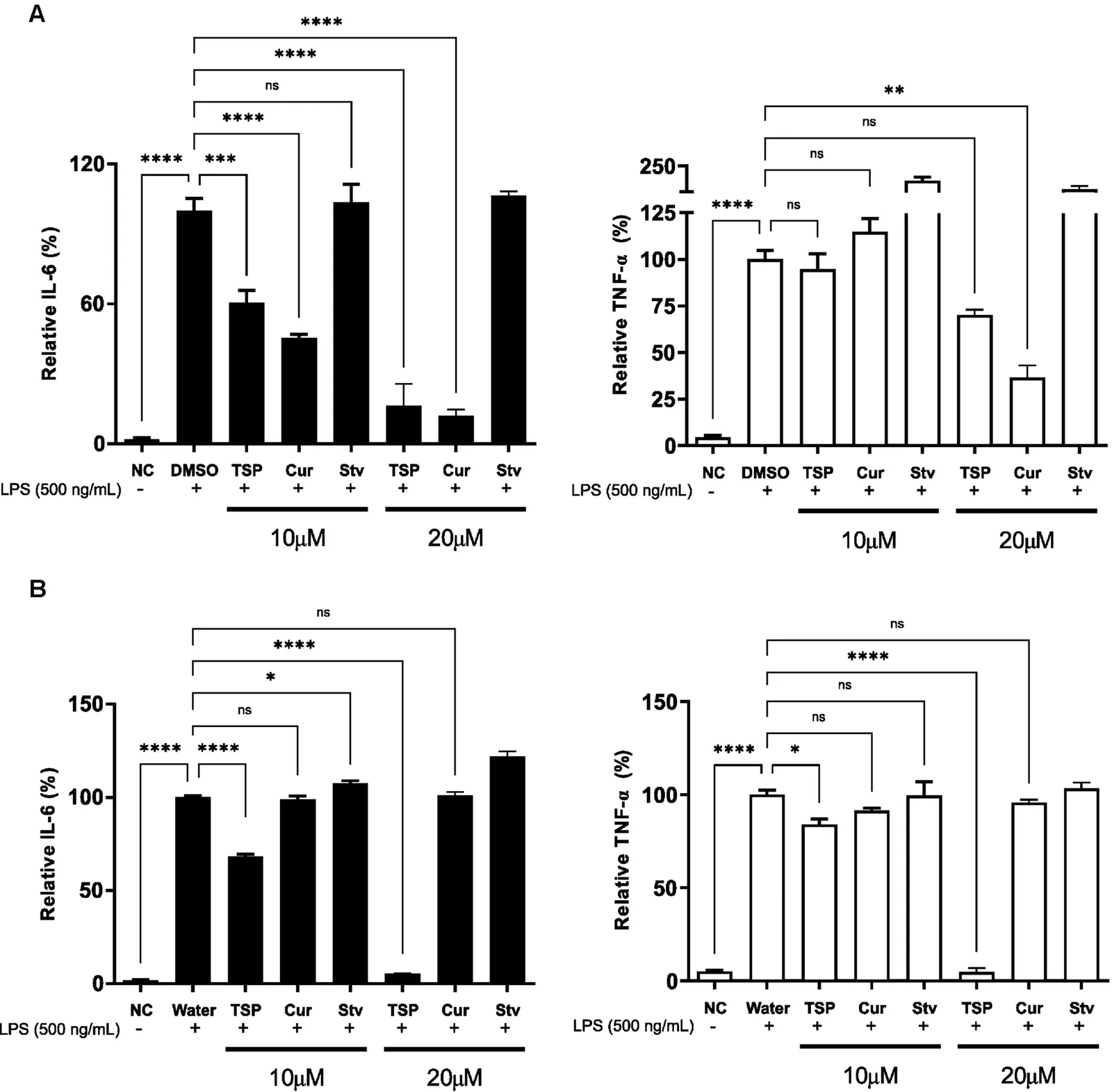
TSP shows superior anti-inflammatory activity under aqueous conditions in LPS-stimulated mouse macrophages. IL-6 and TNF-α levels were measured by ELISA in the culture supernatants of RAW 264.7 cells. Cells were stimulated with LPS (500 ng/mL) and treated for 6 h with the indicated samples dissolved in either (**A**) DMSO or (**B**) distilled water. For the 10 μM treatment, the concentrations used were 3.68 μg/mL for curcumin (Cur), 198.05 μg/mL for TSP, and 141.47 μg/mL for stevioside (Stv). Data are presented as mean ± SEM from three independent experiments. NC, normal control; TSP, water-soluble curcumin complex; Cur, curcumin; Stv, stevioside. Statistical analysis was performed using one-way ANOVA followed by Dunnett’s *post-hoc* test. Statistical significance was determined relative to the DMSO control in (**A**) and the water control in (**B**). Significance levels are indicated as follows: *p < 0.05, **p < 0.01, ***p < 0.001, and ****p < 0.0001; ns, not significant.

To compare the anti-inflammatory activities of TSP and native curcumin, RAW 264.7 macrophages were stimulated with LPS and treated with TSP, curcumin, or stevioside. TSP was administered at doses corresponding to 10 or 20 μM curcumin based on its curcumin content, while native curcumin was tested at the same concentrations and stevioside was applied at amounts equivalent to those present in the corresponding TSP doses. First, to assess their intrinsic anti-inflammatory activity under lipophilic conditions, all samples were dissolved in DMSO. Under these conditions, both TSP and curcumin significantly suppressed IL-6 and TNF-α production (*P* < 0.0001 for both), with curcumin showing slightly greater activity than TSP (Fig. 1A). We next evaluated their activities under aqueous conditions by dissolving the samples in distilled water. In contrast to curcumin (IL-6, *P* = 0.9944; TNF-α, *P* = 0.9038) and stevioside (IL-6, P > 0.999; TNF-α, *P* = 0.9618), TSP retained potent anti-inflammatory activity and markedly reduced the production of IL-6 and TNF-α (*P* < 0.0001 for both; Fig. 1B). These results indicate that TSP exhibits superior anti-inflammatory efficacy under aqueous conditions, likely owing to its enhanced water solubility and resulting improvement in biological availability under physiologically relevant conditions.

### TSP Alleviates DSS-Induced Colitis More Effectively Than Native Curcumin

**Fig. 2:**
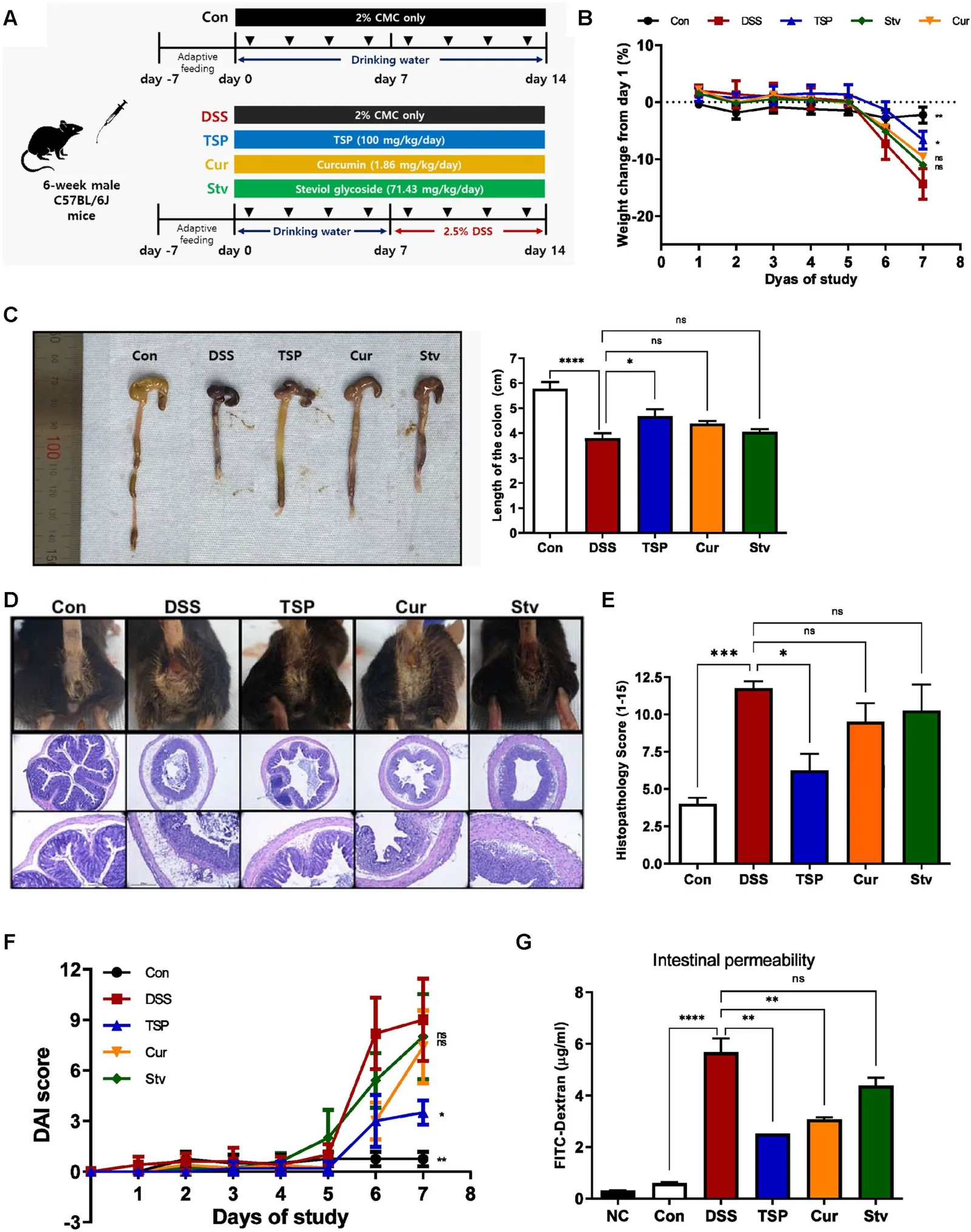
TSP alleviates DSS-induced colitis in mice. Experimental scheme of the DSS-induced colitis model. Mice were orally administered vehicle (2% CMC), curcumin (Cur), stevioside (Stv), or TSP every other day for 14 days. Colitis was induced by 2.5% DSS in drinking water during the last 7 days. (**B**) Body weight changes during DSS administration (days 7-14). (**C**) Representative images of colon length at day 14. (**D**) Representative macroscopic images and H&E-stained colon sections at day 14 (distal colon; scale bar = 200 μm). (**E**) Histological scores. (**F**) Disease activity index (DAI) scores on day 14. (**G**) Intestinal permeability assessed by serum FITC-dextran levels. The NC group represents a baseline measurement from normal mice without FITC-dextran administration. Data are presented as mean ± SEM (*n* = 8 per group). Data were analyzed using a two-way ANOVA followed by Dunnett’s *post-hoc* test. *p < 0.05, ** *p* < 0.01, *** *p* < 0.001, **** *p* < 0.0001 relative to the DSS group; ns, not significant.

To evaluate the therapeutic efficacy of TSP *in vivo*, we employed a DSS-induced mouse colitis model (Fig. 2A). DSS administration caused a rapid decline in body weight in the DSS control group, whereas this loss was significantly attenuated in the TSP-treated group (*P* = 0.0137), indicating greater protection than that observed with native curcumin treatment (Fig. 2B). Consistent with this finding, TSP markedly prevented DSS-induced colon shortening compared with the DSS group (*p* = 0.0206, Fig. 2C). Macroscopic signs of colitis, including rectal bleeding and diarrhea, were also substantially reduced by TSP treatment after DSS exposure (Fig. 2D). Histopathological analysis further supported these clinical improvements. Whereas the DSS group exhibited severe epithelial damage, tissue necrosis, and inflammatory cell infiltration, these pathological changes were markedly ameliorated in the TSP-treated group (*P* = 0.0116), resulting in histological scores that approached those of the normal control group (Fig. 2D and 2E). In line with these observations, disease activity index (DAI) scores were significantly lower in the TSP group than in the DSS group (*p* = 0.0434, Fig. 2F). In addition, intestinal barrier function was effectively preserved by TSP treatment, as demonstrated by the significant reduction in serum FITC-dextran levels relative to the DSS group (*p* = 0.0067, Fig. 2G). Importantly, the protective effects of TSP were consistently greater than those observed with native curcumin across clinical, histological, and barrier-function parameters. In contrast, stevioside alone did not confer significant benefit (*p* = 0.0852), indicating that the superior anti-colitic efficacy of TSP was primarily driven by the solubilized curcumin component.

### TSP Alters Gut Microbial Diversity and Community Structure Relative to the DSS Group

**Fig. 3:**
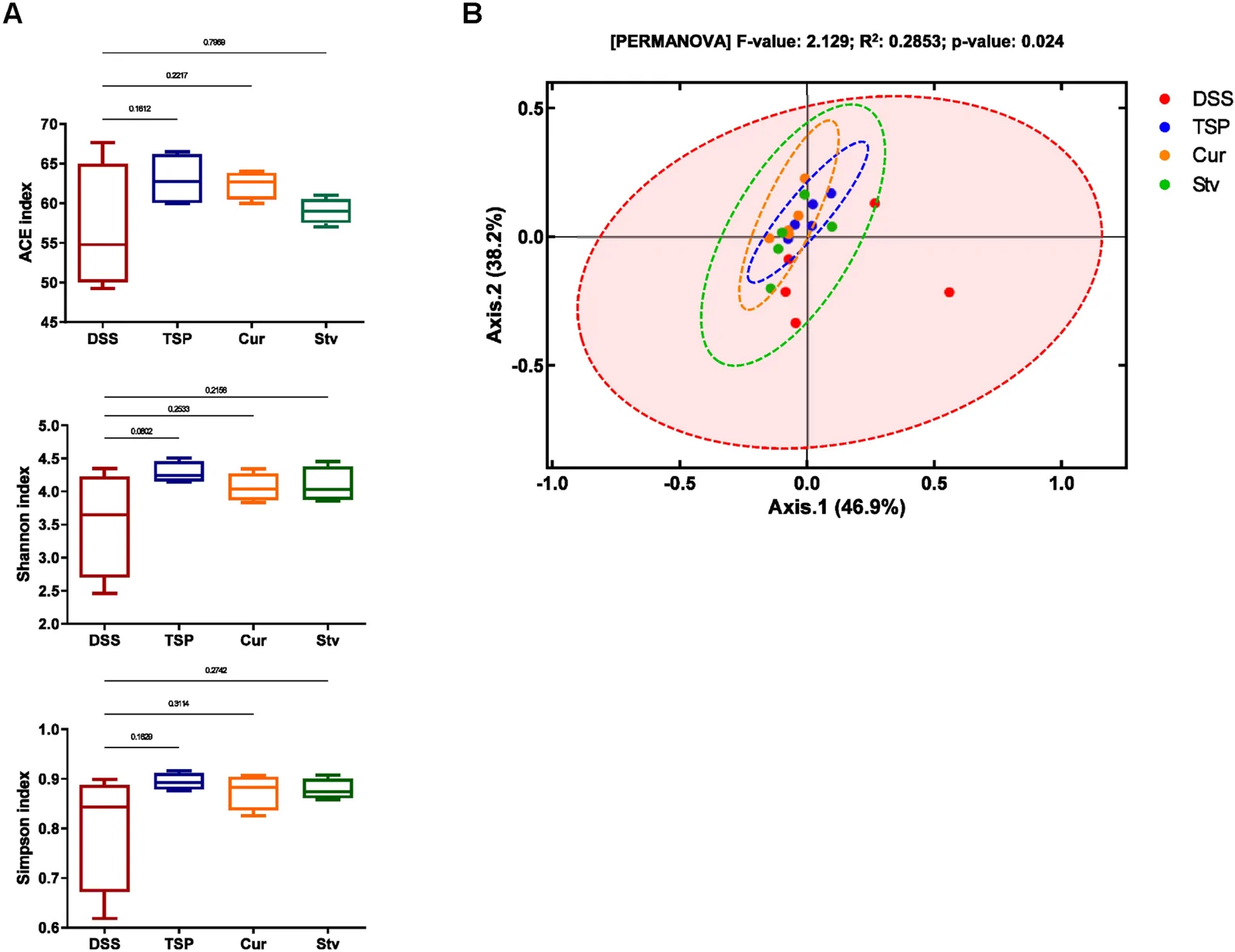
TSP is associated with altered gut microbial diversity and community structure relative to the DSS group. (**A**) Alpha-diversity indices of the gut microbiota, including ACE, Shannon, and Simpson indices. Statistical significance was determined relative to the DSS group using one-way ANOVA followed by Dunnett’s *post-hoc* test, and the exact *P*-values are shown above the corresponding comparisons. (**B**) Beta-diversity analysis by principal coordinate analysis (PCoA) based on Bray-Curtis dissimilarity. Each point represents an individual sample, and colored ellipses indicate group-level clustering. Differences in microbial community structure among groups were tested using PERMANOVA (F = 2.129, R² = 0.2853, *p* = 0.024). Data are presented as mean ± SEM (*n* = 5 per group).

To determine whether TSP modulates gut microbial community structure in DSS-induced colitis, we first assessed alpha-diversity using the ACE, Shannon, and Simpson indices (Fig. 3A). DSS treatment markedly reduced microbial richness and evenness and was associated with substantial inter-individual variation. In contrast, TSP treatment showed higher alpha-diversity indices than the DSS group and reduced the variability observed among DSS-treated mice, suggesting treatment-associated changes in the disrupted gut microbial community. Compared with native curcumin, TSP showed a clearer restorative trend in microbial diversity and community stability. Interestingly, the Stv group also exhibited a partial improvement in alpha-diversity relative to the DSS group, although this effect was not accompanied by clear protection against DSS-induced colitic phenotypes. Beta-diversity analysis further supported these findings (Fig. 3B). PERMANOVA based on Bray-Curtis distance dissimilarity revealed that the DSS group displayed a broadly dispersed clustering pattern (pseudo-F = 2.129, R^2^ = 0.2853, *p* = 0.024), whereas the TSP-treated group formed a tighter and more distinct cluster, suggesting a distinct community pattern relative to the DSS group. Collectively, these findings indicate that TSP was associated with changes in microbial diversity and community structure compared with the DSS group.

### TSP Shifts Gut Microbiota Composition Relative to the DSS Group

**Fig. 4:**
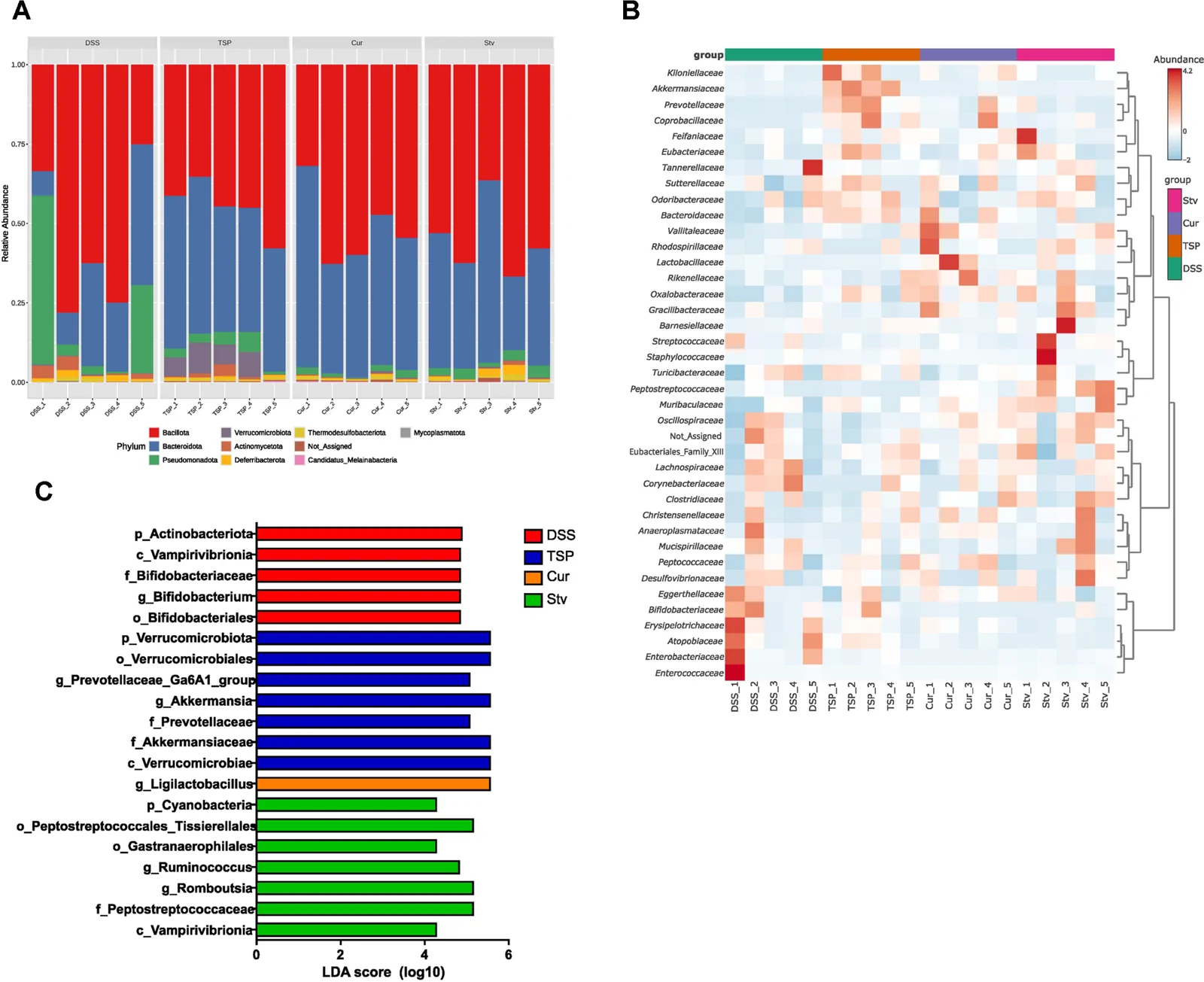
TSP modulates gut microbiota composition relative to the DSS group. (**A**) Relative abundances of gut microbial phyla across the experimental groups (*n* = 5 per group). (**B**) Family-level Heatmap showing microbial composition patterns across the experimental groups. Colors represent row-wise z-scores, with red and blue indicating abundance above and below the family-specific mean, respectively. (**C**) Linear discriminant analysis effect size (LEfSe) identifying discriminative microbial taxa (LDA score > 4.0).

To further characterize the microbial changes associated with TSP treatment, we analyzed gut microbiota composition at the phylum level (Fig. 4A). DSS-induced colitis caused a marked shift in the microbial community structure, whereas TSP treatment promoted a distinct compositional profile compared with the DSS group. Among these changes, the relative abundance of *Verrucomicrobiota* was higher in the TSP group. Family-level heatmap analysis further showed distinct microbial compositional patterns among the experimental groups, including a treatment-associated shift in the TSP group relative to the DSS group (Fig. 4B). This heatmap was used as an exploratory overview of family-level compositional patterns rather than as a statistical test for differentially abundant taxa. To identify the key microbial features distinguishing the treatment groups, we performed linear discriminant analysis effect size (LEfSe) analysis (Fig. 4C). Notably, the beneficial mucin-degrading genus *Akkermansia* and its parent phylum *Verrucomicrobiota* were identified as major biomarkers enriched in the TSP group (*P* = 0.0325). Collectively, these findings suggest that TSP exerts anti-colitic effects, at least in part, in association with enrichment of selected beneficial commensals and microbiota compositional shifts relative to the DSS group.

### TSP Enriches Key SCFA-Associated Bacterial Taxa in the Gut Microbiota

**Fig. 5:**
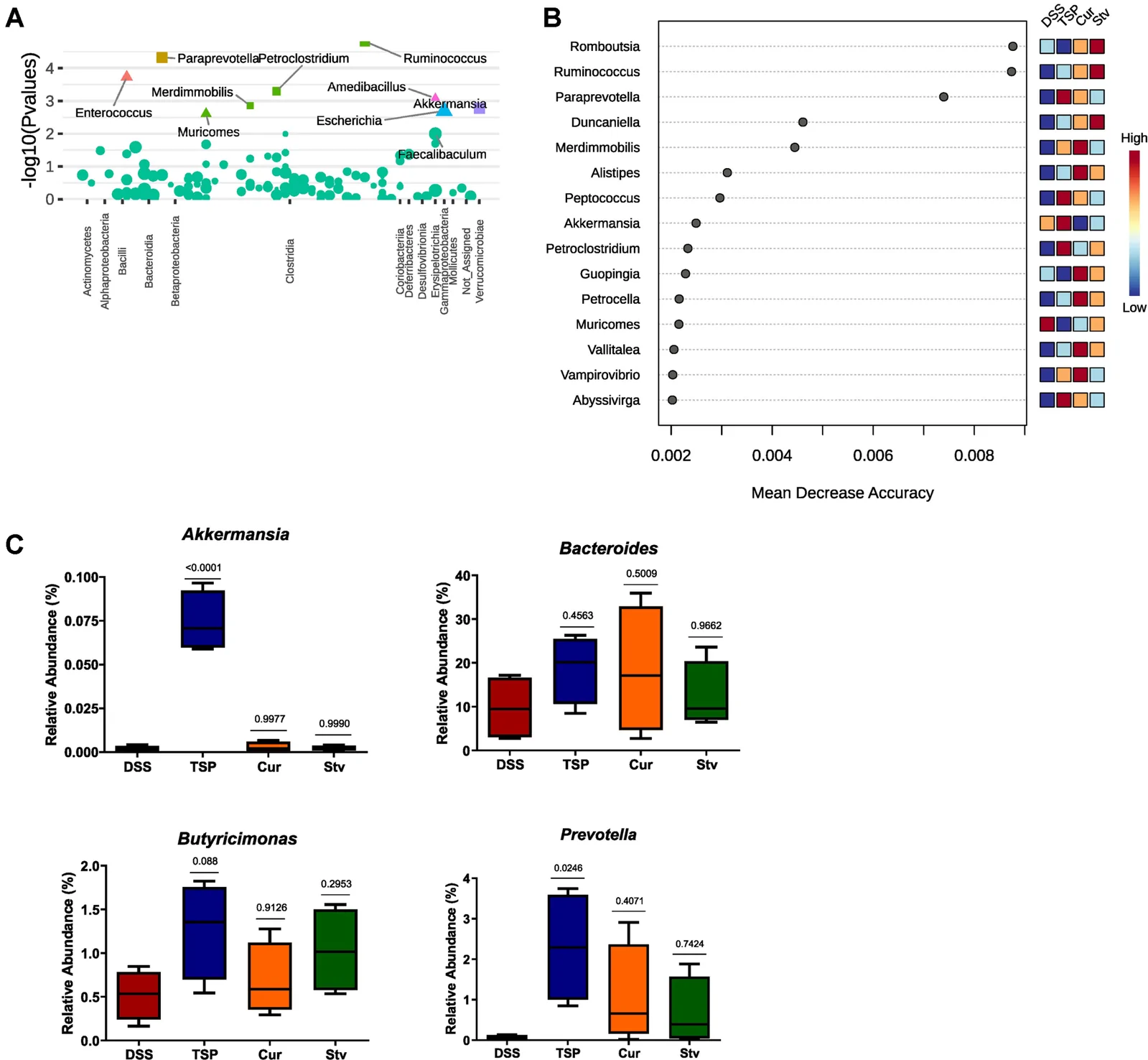
TSP enriches key SCFA-associated bacterial taxa in the gut microbiota. (**A**) Univariate analysis of bacterial genera across the experimental groups. Each point represents an OTU/ASV; point size reflects its log total count, and the y-axis represents -log10 (*P*-value) from the omnibus test. Colored non-circular symbols identify the labeled taxa. (**B**) Random Forest analysis showing the major discriminative microbial taxa. (**C**) Relative abundances of major SCFA-associated genera. Data are presented as mean ± SEM (*n* = 5 per group). Statistical significance was determined relative to the DSS group using one-way ANOVA followed by Dunnett’s *post-hoc* test, and the exact *P*-values are shown above the corresponding comparisons.

To identify the bacterial taxa underlying the TSP-associated restructuring of the gut microbiota, we performed a taxonomic analysis at the genus level. Several genera showed differential relative-abundance patterns among the experimental groups, with notable differences observed in taxa belonging to the classes *Clostridia*, *Bacteroidia*, and *Verrucomicrobiae* (Fig. 5A). To further define the microbial features that most strongly distinguished the groups, we applied a Random Forest algorithm (Fig. 5B). This analysis identified *Romboutsia*, *Ruminococcus*, *Paraprevotella*, *Duncaniella*, and *Akkermansia* as major discriminative taxa. Given the importance of short-chain fatty acids (SCFAs) in intestinal immune homeostasis, we next focused on SCFA-associated genera identified in our dataset (Fig. 5C). Among these, *Akkermansia* showed lower relative abundance in the DSS group and higher relative abundance in the TSP group. In addition, *Prevotella* showed an increasing trend in the TSP group, whereas *Bacteroides* and *Butyricimonas* also exhibited treatment-associated changes relative to the DSS group. Overall, these changes were more pronounced in the TSP group than in the native curcumin group, suggesting that TSP treatment was associated with enrichment patterns of selected SCFA-associated commensals in DSS-induced colitis.

To account for the compositional nature of 16S rRNA sequencing data, the principal genus-level taxa were further evaluated using ANCOM-BC with Benjamini–Hochberg false discovery rate correction. The full ANCOM-BC output is provided in Supplementary Table S1 and S2. This analysis showed that *Akkermansia* and selected SCFA-associated genera exhibited directional changes that were generally consistent with the patterns observed in the LEfSe and Random Forest analyses. Although not all individual genera reached statistical significance after FDR correction, the ANCOM-BC results supported an overall treatment-associated shift in genus-level microbial composition in the TSP group relative to the DSS group. These findings suggest that the microbiota changes associated with TSP treatment should be interpreted as coordinated compositional shifts rather than as definitive changes in every individual taxon.

### TSP Increases the Predicted Abundance of Microbial Pathways Associated with SCFA Biosynthesis

**Fig. 6:**
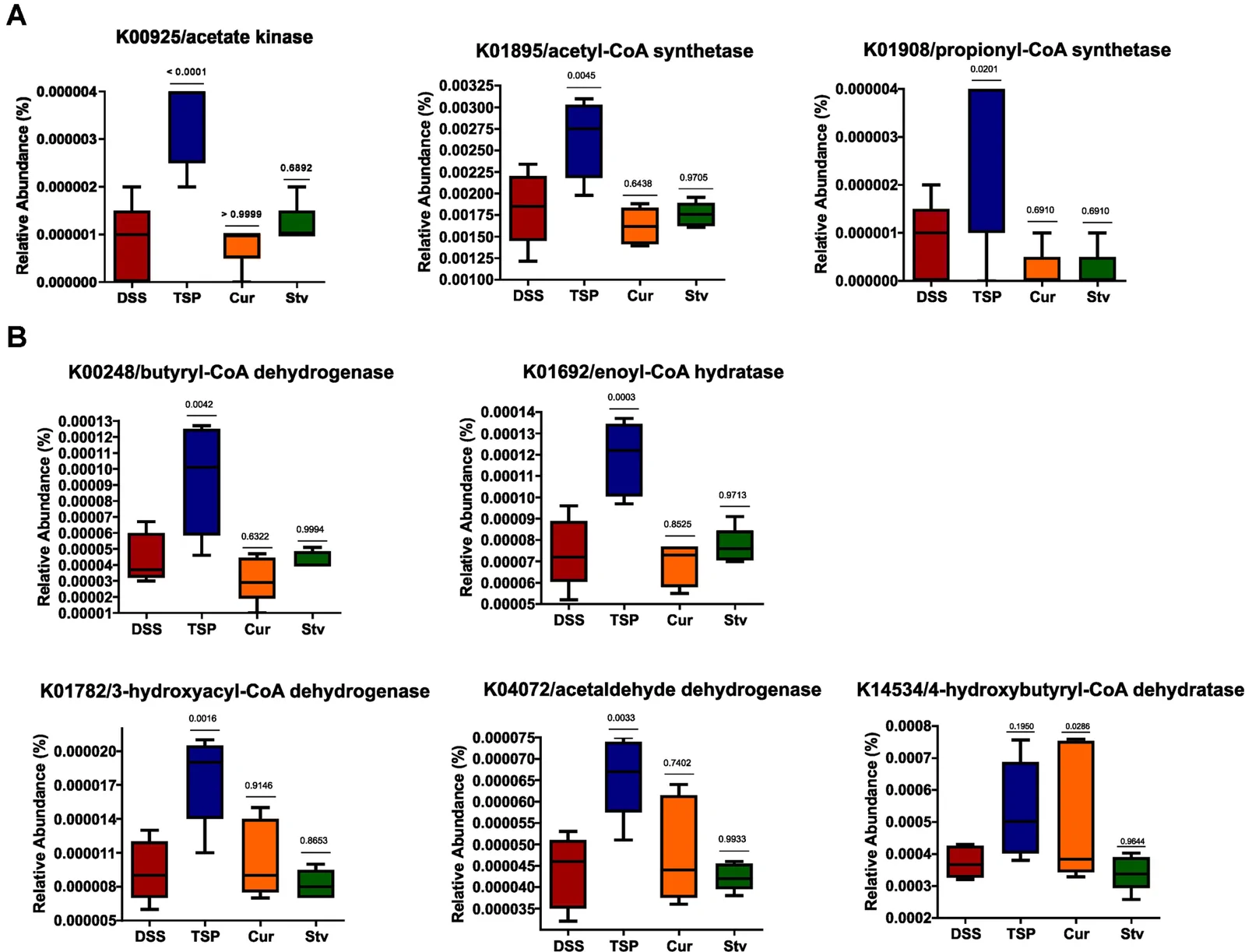
TSP increases the predicted abundance of microbial pathways associated with SCFA biosynthesis. (**A**) Predicted relative abundance of microbial pathways associated with acetate and propionate metabolism. (**B**) Predicted relative abundance of microbial pathways associated with butyrate biosynthesis. Data are presented as mean ± SEM (*n* = 5 per group). Statistical significance was determined relative to the DSS group using one-way ANOVA followed by Dunnett’s *post-hoc* test, and the exact *P*-values are shown above the corresponding comparisons.

To explore the functional implications of the TSP-induced changes in gut microbiota composition, we performed functional prediction analysis using Tax4Fun. Consistent with the enrichment of SCFA-associated bacterial taxa, the TSP group showed an increased predicted abundance of microbial pathways related to SCFA biosynthesis compared with the DSS group. In particular, pathways associated with acetate and propionate metabolism (Fig. 6A) as well as butyrate biosynthesis (Fig. 6B) were enriched in the TSP-treated group. These findings suggest that TSP-mediated remodeling of the gut microbiota is accompanied by an increased functional potential for SCFA production in DSS-induced colitis.

## Discussion

In the present study, we demonstrated that TSP, a previously developed water-soluble curcumin-stevioside glycoside, exerted superior therapeutic efficacy to native curcumin in a DSS-induced colitis model. The key finding of this study is that TSP showed greater anti-colitic effects than native curcumin, as evidenced by improved clinical symptoms, preservation of colon length, attenuation of histopathological damage, and restoration of intestinal barrier integrity. These *in vivo* findings were supported by the *in vitro* observation that TSP retained potent anti-inflammatory activity under aqueous conditions, where native curcumin showed minimal efficacy. Together, these results suggest that the enhanced practical efficacy of TSP is closely associated with its improved aqueous solubility, which may facilitate greater biological availability under physiologically relevant conditions.

Various strategies, including nanoparticles, liposomes, and co-administration with adjuvants such as piperine, have been explored to improve the bioavailability of curcumin [13, 17, 29-31]. However, these approaches often face practical limitations, including variable absorption, formulation complexity, instability during storage, and challenges in large-scale production. In this context, glycosylation represents a promising alternative strategy for improving the physicochemical properties of curcumin. TSP, a water-soluble curcumin glycoside, appears to offer a practical advantage by markedly enhancing aqueous solubility without requiring additional carriers or bioavailability enhancers. Consistent with this, our *in vitro* results showed that under aqueous conditions, native curcumin exhibited little anti-inflammatory activity, whereas TSP retained potent suppression of IL-6 and TNF-α production (Fig. 1A and 1B). These findings suggest that the superior efficacy of TSP is not attributable solely to its solubility as a formulation property, but also to improved functional activity under physiologically relevant conditions. Therefore, TSP may represent a more effective curcumin-based approach for intestinal inflammatory disorders than native curcumin.

In our *in vivo* experiments, TSP showed clearly greater therapeutic efficacy than native curcumin in DSS-induced colitis. Whereas curcumin treatment produced only limited improvement in body weight loss and intestinal tissue injury, TSP treatment significantly ameliorated multiple indicators of disease severity, including colon shortening, histopathological damage, disease activity index, and intestinal permeability (Fig. 2). Previous studies have shown that curcumin can reinforce intestinal barrier function, reduce circulating lipopolysaccharide levels, and promote acidic mucus secretion, thereby contributing to the protection of intestinal structure and function [32-34]. Although the present study did not directly investigate these specific mechanisms, our findings suggest that TSP may confer stronger protection against colitis than native curcumin by improving the practical availability of curcumin under physiological conditions. Taken together, these results indicate that the superior aqueous solubility of TSP is likely to contribute to its enhanced *in vivo* anti-colitic efficacy.

In addition to its direct anti-inflammatory activity and protective effects against colitic injury, TSP exerted a pronounced modulatory effect on the gut microbiota, a key factor in IBD pathogenesis. DSS treatment induced marked dysbiosis, characterized by reduced microbial diversity, disruption of community structure, and expansion of inflammation-associated taxa (Figs. 3 and 4A). Notably, the DSS group showed an increased relative abundance of the phylum *Pseudomonadota*, which is commonly associated with inflammatory dysbiosis (Fig. 4A). In contrast, TSP treatment was associated with higher alpha-diversity indices and a distinct microbial community structure compared with the DSS group. However, because microbiota data from the normal control group were not available, these changes should not be interpreted as definitive restoration toward a healthy baseline composition. (Fig. 3). These restorative effects appeared more consistent in the TSP group than in the native curcumin group, further supporting the superior *in vivo* efficacy of TSP. At the taxonomic level, TSP suppressed the DSS-associated expansion of *Pseudomonadota* while enriching beneficial commensals, particularly *Verrucomicrobiota* and *Akkermansia* (Fig. 4). This reduction may be relevant because *Pseudomonadota* include major LPS-producing bacteria [35], suggesting that TSP may help reduce microbiota-derived inflammatory stimuli in the intestine. In addition, taxa associated with SCFA metabolism and intestinal homeostasis, including *Bacteroides*, *Butyricimonas*, and *Prevotella* [36-41], showed restorative changes in the TSP group (Fig. 5C). These compositional shifts were accompanied by an increased predicted abundance of microbial pathways related to acetate, propionate, and butyrate biosynthesis (Fig. 6), suggesting that TSP may promote a gut environment favorable for epithelial protection and immune regulation. Interestingly, stevioside alone showed a partial effect on microbial diversity relative to the DSS group (Fig. 3A), although this change was not accompanied by clear improvement in clinical or histopathological outcomes (Fig. 2D-2F). Together, these findings suggest that the superior anti-colitic efficacy of TSP is likely driven by the combined contribution of solubilized curcumin-mediated anti-inflammatory activity and microbiota remodeling, rather than by microbiota modulation alone.

Several limitations of this study should be acknowledged. First, although TSP showed superior anti-colitic efficacy compared with native curcumin, we did not directly measure the pharmacokinetics, systemic absorption, or tissue distribution of TSP and its metabolites. Therefore, the proposed link between improved aqueous solubility and enhanced *in vivo* availability requires further validation. Second, although TSP treatment was associated with enrichment of SCFA-associated taxa and an increased predicted abundance of microbial pathways related to SCFA biosynthesis, actual SCFA concentrations in the intestine were not quantified in this study. Third, microbiota sequencing data from the normal vehicle-treated control group were not available in this study; therefore, the microbiota findings should be interpreted as treatment-associated differences relative to the DSS group rather than definitive restoration to a normal microbial state. In addition, the molecular mechanisms underlying the protective effects of TSP on intestinal inflammation, barrier integrity, and mucus homeostasis were not directly examined. Despite these limitations, our findings consistently demonstrate that TSP exerts superior anti-inflammatory and anti-colitic effects compared with native curcumin in both *in vitro* and *in vivo* settings. Collectively, these results suggest that TSP is a promising curcumin-based candidate that may overcome the practical limitations of native curcumin and warrants further mechanistic and translational investigation for intestinal inflammatory disorders.

## Supplemental Materials

Supplementary data for this paper are available on-line only at http://jmb.or.kr.
