# Supplementary material for: A Water-Soluble Curcumin Glycoside Exhibits Superior Anti-Colitic Efficacy over Native Curcumin in DSS-Induced Colitis through Anti-Inflammatory Activity and Gut Microbiota Modulation
Source: J Microbiol Biotechnol. 2026 Aug 5;36:e2604082. doi: 10.4014/jmb.2604.04082 (PMC13500919; doi:10.4014/jmb.2604.04082)
Supplement: Supplementary file 1 [file jmb-36-e2604082-supple.pdf]

Supplementary Table 1. ANCOM-BC differential abundance analysis at the genus level

taxon	comparison	log2FC_clr_median	log2FC_clr_mean	log2FC_clr_ci_low	log2FC_clr_ci_high	p_value	q_value	total_count
Turicibacter	TSP_vs_DSS	1.371415362	1.368926289	1.142047489	1.53344331	0.347515286	0.760359392	72642
Kineothrix	TSP_vs_DSS	-0.159222827	-0.158225177	-0.328241833	0.02331462	0.856192161	0.879023952	70740
Duncaniella	TSP_vs_DSS	1.220051137	1.217984939	1.048260638	1.379007149	0.077167879	0.760359392	53394
Bacteroides	TSP_vs_DSS	1.369126883	1.370522965	1.1886453	1.519195448	0.223625855	0.760359392	47788
Phocaeicola	TSP_vs_DSS	0.97268049	0.964770314	0.799487025	1.097593709	0.402393407	0.760359392	45692
Alistipes	TSP_vs_DSS	0.086162434	0.087237344	-0.08681323	0.257774687	0.913008838	0.918976216	39461
Escherichia	TSP_vs_DSS	-1.768154997	-1.786248855	-2.049630581	-1.551880759	0.490425456	0.760359392	34306
Faecalibaculum	TSP_vs_DSS	-1.967633657	-1.967925103	-2.184636693	-1.758630498	0.330044183	0.760359392	24274
Parabacteroides	TSP_vs_DSS	0.0113653	0.005636456	-0.163150269	0.184037439	0.958263223	0.958263223	16673
Lawsonibacter	TSP_vs_DSS	-0.601742308	-0.598360177	-0.775036314	-0.438991485	0.417003262	0.760359392	16576
Ligilactobacillus	TSP_vs_DSS	-0.287966021	-0.295937393	-0.475252897	-0.14213945	0.664917379	0.777287225	16313
Muribaculum	TSP_vs_DSS	-0.936299827	-0.946143325	-1.142828013	-0.780108317	0.325398701	0.760359392	13905
Akkermansia	TSP_vs_DSS	3.907434897	3.910915467	3.471315008	4.34664809	0.093495889	0.760359392	11460
Heminiphilus	TSP_vs_DSS	-0.175912903	-0.175834012	-0.422864953	0.08952392	0.90478813	0.916693236	11253
Paraprevotella	TSP_vs_DSS	6.622227724	6.809341958	5.921440289	8.355171058	0.017059693	0.458234688	10096
Lachnoclostridium	TSP_vs_DSS	0.893630654	0.885316897	0.686858133	1.060473482	0.158361035	0.760359392	9496
Unclassified	TSP_vs_DSS	0.135617729	0.135014347	-0.047732989	0.305278711	0.75375753	0.818822371	9340
Romboutsia	TSP_vs_DSS	-2.245944348	-2.158421991	-4.847388276	0.178753377	0.492930559	0.760359392	9090
Simiaoa	TSP_vs_DSS	1.27040023	1.27269124	0.941247832	1.544771994	0.326640514	0.760359392	8275
Marseillibacter	TSP_vs_DSS	-0.530984764	-0.538760655	-0.772091014	-0.353551552	0.294658198	0.760359392	7520
Paramuribaculum	TSP_vs_DSS	1.300937667	1.315757431	1.158873124	1.518327785	0.047292518	0.606920645	7407
Lacrimispora	TSP_vs_DSS	0.462361	0.464447401	0.236434327	0.705740118	0.647151491	0.777287225	7368
Mediterraneibacter	TSP_vs_DSS	-0.132798641	-0.136325194	-0.315298043	0.028508757	0.84735302	0.875787685	6960
Parasutterella	TSP_vs_DSS	0.538834182	0.53742593	0.307745942	0.735355812	0.680026008	0.777287225	6731
Butyricimonas	TSP_vs_DSS	0.53336597	0.524950719	0.276644426	0.704735838	0.582952116	0.760359392	6433
Acetatifactor	TSP_vs_DSS	-0.341723537	-0.355977983	-0.631186396	-0.114301123	0.773671086	0.827398244	5455
Vallitalea	TSP_vs_DSS	0.742957263	0.724367771	0.460838102	0.937696257	0.459945646	0.760359392	5406

Mucispirillum	TSP_vs_DSS	-1.858440026	-1.860353564	-2.035017674	-1.651008722	0.019733716	0.458234688	5329
Clostridium	TSP_vs_DSS	0.567797134	0.56767337	0.334803256	0.845347907	0.50415605	0.760359392	5301
Bifidobacterium	TSP_vs_DSS	-1.412016077	-1.437468252	-1.870300619	-1.153471033	0.494508311	0.760359392	5234
Vescimonas	TSP_vs_DSS	-0.992998791	-0.999059578	-1.221526714	-0.824537638	0.154441672	0.760359392	5154
Intestinimonas	TSP_vs_DSS	-0.811029717	-0.814074197	-1.004445685	-0.627933569	0.015781593	0.458234688	4998
Desulfovibrio	TSP_vs_DSS	-0.346585744	-0.349573933	-0.551897015	-0.143818038	0.617174831	0.760359392	4468
Flintibacter	TSP_vs_DSS	-0.928025837	-0.927650389	-1.159340003	-0.716551675	0.014236429	0.458234688	4382
Ruminiclostridium	TSP_vs_DSS	0.350760702	0.345042301	0.107293387	0.604892258	0.789743311	0.82752557	4283
Ruminococcus	TSP_vs_DSS	7.446454875	7.604437798	5.935155638	10.32596796	0.013784901	0.458234688	4256
Mammaliicoccus	TSP_vs_DSS	1.769128996	1.857601184	0.999355089	3.224457318	0.301743139	0.760359392	3810
Anaerotignum	TSP_vs_DSS	0.120183075	0.110277863	-0.18508938	0.393231231	0.892250027	0.909976849	3414
Sangeribacter	TSP_vs_DSS	-0.454801693	-0.440613863	-0.667808924	-0.187351157	0.60311616	0.760359392	3235
Neglectibacter	TSP_vs_DSS	-0.40542222	-0.412521889	-0.676471901	-0.183946639	0.700604149	0.793331169	2840
Butyrivibrio	TSP_vs_DSS	0.048387728	0.092548077	-2.345967675	2.380603169	0.789910772	0.82752557	2593
Anaeromicropila	TSP_vs_DSS	-0.643070341	-0.637589052	-0.949245559	-0.271526194	0.500136267	0.760359392	2464
Christensenella	TSP_vs_DSS	2.34981153	2.36918595	1.531030785	3.632612393	0.207196379	0.760359392	2461
Barnesiella	TSP_vs_DSS	-2.384335345	-2.532088592	-5.065404815	-0.37541614	0.486534528	0.760359392	2281
Oscillibacter	TSP_vs_DSS	-0.806515751	-0.802367405	-1.084164693	-0.565755702	0.30878858	0.760359392	2266
Guopingia	TSP_vs_DSS	0.65411302	0.651857507	0.136524712	1.199255225	0.613136913	0.760359392	1993
Staphylococcus	TSP_vs_DSS	1.777215305	1.797450514	1.150039946	2.502139259	0.299597566	0.760359392	1854
Limosilactobacillus	TSP_vs_DSS	2.045835098	2.21108342	1.178292598	3.730115109	0.370358147	0.760359392	1723
Catenibacillus	TSP_vs_DSS	0.658518405	0.767486553	-1.107937617	3.294818814	0.760335059	0.818822371	1660
Dehalobacterium	TSP_vs_DSS	-0.445729176	-0.46026206	-0.795862285	-0.12914187	0.522396961	0.760359392	1435
Allobaculum	TSP_vs_DSS	-9.127826696	-9.340978385	-13.00471384	-6.835070871	0.004544712	0.458234688	1327
Angelakisella	TSP_vs_DSS	-1.230959763	-1.250030699	-1.768208175	-0.742864854	0.155097961	0.760359392	1233
Pseudoflavonifractor	TSP_vs_DSS	-2.403846592	-2.400713188	-2.713911165	-2.087253437	0.021396863	0.458234688	1222
Acetivibrio	TSP_vs_DSS	2.383609676	2.45039597	1.376203255	4.044539506	0.218165983	0.760359392	1218
Butyricicoccus	TSP_vs_DSS	-1.110241815	-1.127388673	-1.551295131	-0.716518495	0.117879877	0.760359392	1156
Marasmitruncus	TSP_vs_DSS	-1.531229304	-1.55308669	-2.043820832	-1.110113473	0.32464863	0.760359392	1082

Feifania	TSP_vs_DSS	0.408333945	0.411987956	0.077161551	0.861365867	0.58853477	0.760359392	1001
Roseburia	TSP_vs_DSS	2.258173329	2.373045294	1.067800209	4.36603057	0.32405856	0.760359392	994
Peptococcus	TSP_vs_DSS	0.804381575	0.808753625	0.072444528	1.533999817	0.571162514	0.760359392	986
Vampirovibrio	TSP_vs_DSS	1.958001421	2.128575186	1.160386237	4.689023338	0.214008723	0.760359392	916
Luoshenia	TSP_vs_DSS	0.908101684	0.933986932	0.331648182	1.493524612	0.355482219	0.760359392	912
Muricomes	TSP_vs_DSS	-3.883359923	-3.945223199	-5.21879146	-3.152427779	0.034369409	0.529288893	910
Aestuariispira	TSP_vs_DSS	1.85239392	2.003799919	-0.13274205	4.923498289	0.544573811	0.760359392	906
Qiania	TSP_vs_DSS	0.144312739	0.300676574	-0.532990613	1.625151835	0.818885485	0.852083545	903
Sporofaciens	TSP_vs_DSS	-1.010408351	-1.035083363	-2.789633572	0.668286075	0.646629236	0.777287225	872
Fusimonas	TSP_vs_DSS	-0.875550961	-0.813595228	-2.437583799	0.940587321	0.670879122	0.777287225	856
Bilophila	TSP_vs_DSS	0.372793735	0.401530337	-0.356415213	1.275371388	0.785369427	0.82752557	835
Petroclostridium	TSP_vs_DSS	5.495609027	5.446830366	3.083638908	8.108596137	0.088475514	0.760359392	828
Harryflintia	TSP_vs_DSS	-0.813793285	-0.813584281	-1.23346396	-0.420982131	0.272253268	0.760359392	816
Gracilibacter	TSP_vs_DSS	0.646537875	0.862594764	-0.241810492	2.590914873	0.681388152	0.777287225	810
Petrocella	TSP_vs_DSS	1.821920487	1.944200957	0.587953963	4.128531881	0.397496424	0.760359392	805
Leptogranulimonas	TSP_vs_DSS	-1.358739926	-1.53718412	-3.310518944	-0.467951069	0.565372317	0.760359392	709
Coprococcus	TSP_vs_DSS	0.551668947	0.56967821	0.247024555	1.004655262	0.301747433	0.760359392	683
Anaerotruncus	TSP_vs_DSS	-1.14160874	-1.132815073	-2.734820466	0.771264728	0.574685502	0.760359392	643
Faecalimonas	TSP_vs_DSS	0.532208872	0.709851605	-0.206111628	2.198851588	0.675948815	0.777287225	639
Congzhengia	TSP_vs_DSS	2.190410567	2.337748743	1.346679346	3.959063161	0.220536552	0.760359392	630
Eubacterium	TSP_vs_DSS	1.834129518	1.838239624	1.250841084	2.419837831	0.09734528	0.760359392	587
Marvinbryantia	TSP_vs_DSS	0.767371872	0.673419095	-1.133126063	2.183319283	0.648530339	0.777287225	574
Odoribacter	TSP_vs_DSS	-0.135652166	-0.203281577	-1.974069035	1.420569657	0.759278596	0.818822371	566
Enterococcus	TSP_vs_DSS	-2.706060848	-2.897328141	-6.24618681	0.823649309	0.449849671	0.760359392	547
Rhodospirillum	TSP_vs_DSS	-3.42864216	-3.505876833	-6.982404126	-1.239548398	0.26761989	0.760359392	544
Adlercreutzia	TSP_vs_DSS	-1.974013323	-2.049998979	-3.472416422	-1.223605915	0.27676367	0.760359392	524
Anaerocolumna	TSP_vs_DSS	-2.025504753	-1.996526778	-3.66014689	-0.5400467	0.439758702	0.760359392	523
Ruthenibacterium	TSP_vs_DSS	-0.613886891	-0.615475895	-1.100878198	-0.092599417	0.519667224	0.760359392	523
Streptococcus	TSP_vs_DSS	-1.141690389	-1.150525875	-1.778085494	-0.572745246	0.412024001	0.760359392	511

Dorea	TSP_vs_DSS	1.734192505	1.856965053	0.873552569	3.434803883	0.276712157	0.760359392	496
Faecalicatena	TSP_vs_DSS	0.758625541	0.826921209	-1.907496841	3.279385327	0.720754485	0.810191173	479
Saccharofermentans	TSP_vs_DSS	0.989842414	0.925135154	-2.071288594	3.365352931	0.656067068	0.777287225	462
Waltera	TSP_vs_DSS	0.052625609	0.186796552	-1.139410181	2.213624336	0.74229653	0.817714135	441
Thomasclavelia	TSP_vs_DSS	6.32069309	6.37165356	4.489236739	8.898137002	0.026318369	0.458234688	425
Jeotgalicoccus	TSP_vs_DSS	1.354801584	1.316503794	-2.108119063	4.074704738	0.510574125	0.760359392	421
Eisenbergiella	TSP_vs_DSS	0.519345862	0.542431825	-0.464223872	2.007791853	0.743376486	0.817714135	418
Merdimmobilis	TSP_vs_DSS	5.618834341	5.53410419	3.170191382	8.279905303	0.079584075	0.760359392	345
Anaeroplasma	TSP_vs_DSS	-1.230241596	-1.305110517	-4.241054876	1.11001482	0.611658026	0.760359392	327
Variimorphobacter	TSP_vs_DSS	-0.349235883	-0.363873424	-2.860641155	1.926700817	0.726241905	0.810443865	325
Natranaerovirga	TSP_vs_DSS	1.193954092	1.162479556	-0.693472579	3.116278318	0.572337532	0.760359392	313
Amedibacillus	TSP_vs_DSS	-3.702687329	-3.784851091	-5.433837046	-2.740660409	0.026779949	0.458234688	298
Fumia	TSP_vs_DSS	-2.240250864	-2.206718855	-3.052848528	-1.466768951	0.051562978	0.610822976	297
Lentihominibacter	TSP_vs_DSS	2.482570701	2.762795746	1.290125871	5.083767526	0.105566538	0.760359392	281
Corynebacterium	TSP_vs_DSS	-3.006800447	-3.023739117	-5.875116673	-0.887734316	0.295458271	0.760359392	269
Dysosmobacter	TSP_vs_DSS	-1.012642067	-1.001084395	-2.879387005	1.254238734	0.567151354	0.760359392	267
Anaerobium	TSP_vs_DSS	-0.939611593	-0.907456792	-2.90019363	1.291729393	0.614364897	0.760359392	255
Lactobacillus	TSP_vs_DSS	-2.043451892	-2.157158855	-5.021091587	0.835527126	0.43545869	0.760359392	247
Sinanaerobacter	TSP_vs_DSS	-0.859538403	-0.814781686	-2.642273472	1.239544905	0.586013106	0.760359392	235
Sporobacter	TSP_vs_DSS	-0.904863201	-0.905885474	-2.498234116	0.887595335	0.569595459	0.760359392	229
Ileibacterium	TSP_vs_DSS	-6.543805782	-6.547344486	-9.295800984	-4.012849127	0.043368251	0.606920645	228
Ihubacter	TSP_vs_DSS	1.871322504	2.093064274	0.663928862	4.885025539	0.291976028	0.760359392	192
Enterocloster	TSP_vs_DSS	-1.210018128	-1.105782203	-2.615938078	1.060403382	0.515384224	0.760359392	186
Abyssivirga	TSP_vs_DSS	2.307736074	2.340047461	1.089293712	3.847500713	0.111256345	0.760359392	176
Acutalibacter	TSP_vs_DSS	0.958922433	0.901959419	-2.955898374	4.274513091	0.607051071	0.760359392	176
Xiamenia	TSP_vs_DSS	-1.041758961	-1.127501009	-2.655622165	0.080441021	0.471570015	0.760359392	174
Holdemania	TSP_vs_DSS	1.27277122	1.372666996	0.010953161	3.488509467	0.291158671	0.760359392	148
Zhenpiania	TSP_vs_DSS	-1.092493042	-1.074028772	-2.437833597	0.477249346	0.542879101	0.760359392	142
Paludihabitans	TSP_vs_DSS	-3.871644201	-3.979492962	-6.940487117	-1.096965931	0.115914278	0.760359392	127

Jingyaoa	TSP_vs_DSS	-0.979877537	-1.028363504	-3.524806474	1.889624096	0.578524788	0.760359392	124
Evtepia	TSP_vs_DSS	1.054104462	1.040464644	-3.075759855	4.289237395	0.517511515	0.760359392	119
Tannockella	TSP_vs_DSS	1.10283706	1.112030582	-1.495006128	4.644469116	0.573290888	0.760359392	116
Mageeibacillus	TSP_vs_DSS	-0.774329379	-0.723345861	-3.816595279	2.258627046	0.661114592	0.777287225	102
Berryella	TSP_vs_DSS	1.981837699	2.068179063	-0.33697347	5.08777771	0.389028328	0.760359392	95
Oxalobacter	TSP_vs_DSS	2.021980972	2.166404069	-0.544783954	5.145696985	0.350981789	0.760359392	94
Rhizobium	TSP_vs_DSS	-3.894058295	-3.845155171	-6.710150484	-0.730721283	0.182952477	0.760359392	92
Anaerobacterium	TSP_vs_DSS	0.171260316	0.165298935	-3.832737207	3.896563944	0.591001173	0.760359392	89
Breznakia	TSP_vs_DSS	0.448013625	0.574675338	-2.144723672	3.563035686	0.596219685	0.760359392	85
Raoultibacter	TSP_vs_DSS	1.067775523	1.153383963	-0.001854078	2.882230173	0.300252863	0.760359392	84
Frisingicoccus	TSP_vs_DSS	-3.25829971	-3.284931751	-6.100687046	-0.398402566	0.243902933	0.760359392	80
Gordonibacter	TSP_vs_DSS	-1.152743022	-1.35457276	-3.723093321	0.424286754	0.480433748	0.760359392	76
Lachnotalea	TSP_vs_DSS	-0.81977254	-0.720227027	-3.775800716	2.237657262	0.603438737	0.760359392	67
Hungatella	TSP_vs_DSS	1.386544995	1.417268976	-1.246864077	3.855128707	0.490742207	0.760359392	56
Ructibacterium	TSP_vs_DSS	3.232130108	3.397452656	0.75829568	6.489253784	0.252707497	0.760359392	55
Diplocloster	TSP_vs_DSS	1.904121366	1.974912108	-0.997413908	5.067821075	0.453379394	0.760359392	40
Intestinibacillus	TSP_vs_DSS	-2.510505267	-2.435175589	-5.243800657	0.226803042	0.278362047	0.760359392	30
Alkalibaculum	TSP_vs_DSS	-0.603424036	-0.530590779	-3.680361627	2.683233879	0.567972953	0.760359392	25
Longibaculum	TSP_vs_DSS	-0.442730759	-0.354415272	-4.073846002	3.194482908	0.544351596	0.760359392	25
Monoglobus	TSP_vs_DSS	-0.584285969	-0.669645059	-4.104050471	2.257573245	0.591782958	0.760359392	24
Cuneatibacter	TSP_vs_DSS	0.490342908	0.460660817	-2.503594812	4.441590819	0.511016086	0.760359392	21
Solibaculum	TSP_vs_DSS	0.745543772	0.714452925	-2.650482285	3.941886098	0.573378641	0.760359392	21
Cupriavidus	TSP_vs_DSS	-0.087564785	-0.362158724	-4.293343445	3.309692253	0.513806769	0.760359392	20
Gehongia	TSP_vs_DSS	-0.340932787	-0.385968922	-3.915153456	2.887081472	0.596581509	0.760359392	18
Jutongia	TSP_vs_DSS	0.785785926	0.93010239	-2.561310302	4.419023274	0.559026349	0.760359392	17
Bittarella	TSP_vs_DSS	-0.902450062	-0.956574202	-4.105991872	2.322834401	0.542422245	0.760359392	16
Massiliimalia	TSP_vs_DSS	-1.240948211	-1.180351278	-5.566297405	2.890193017	0.429266154	0.760359392	16
Enteroscipio	TSP_vs_DSS	-3.362026436	-3.164263328	-6.070261315	-0.60455264	0.221586543	0.760359392	15
Hydrogeniiclostridium	TSP_vs_DSS	1.544316039	1.582418129	-1.01789927	4.530894123	0.467291798	0.760359392	12

Arabiibacter	TSP_vs_DSS	-0.355451807	-0.402076766	-4.709191539	3.84931665	0.485430271	0.760359392	11
Aminipila	TSP_vs_DSS	-2.06436272	-1.913590737	-5.95972343	1.938150118	0.3895576	0.760359392	9
Victivallis	TSP_vs_DSS	0.766472476	0.594399717	-2.867902684	3.585103976	0.539419718	0.760359392	9
Massilioclostridium	TSP_vs_DSS	-0.542236123	-0.480839914	-3.840802359	4.246847471	0.504551066	0.760359392	8
Pseudomonas	TSP_vs_DSS	0.434672667	0.440442854	-3.963069871	3.951216445	0.509025116	0.760359392	7
Scatolibacter	TSP_vs_DSS	1.214629921	1.109279695	-2.471544619	4.31047562	0.475318564	0.760359392	5
Blautia	TSP_vs_DSS	-1.145917596	-1.12953141	-4.468257449	2.400293967	0.504649825	0.760359392	4
Robinsoniella	TSP_vs_DSS	-0.689347679	-0.612292824	-4.569357122	3.458679393	0.499750785	0.760359392	4
Vagococcus	TSP_vs_DSS	-0.417355287	-0.528235706	-4.222246075	3.148578194	0.476516861	0.760359392	4
Pusillibacter	TSP_vs_DSS	0.300038726	0.227188873	-3.230480727	3.467857651	0.528250382	0.760359392	3
Shigella	TSP_vs_DSS	-1.015729611	-0.960211839	-4.72458653	3.352080247	0.48174488	0.760359392	3
Turicibacter	Cur_vs_DSS	0.90663411	0.91593733	0.671074791	1.130234394	0.518370966	0.841239962	72642
Kineothrix	Cur_vs_DSS	0.136198712	0.136127192	-0.056557132	0.296035741	0.885703428	0.901226516	70740
Duncaniella	Cur_vs_DSS	1.661542108	1.660918986	1.505909375	1.833673374	0.025912459	0.626677373	53394
Bacteroides	Cur_vs_DSS	1.324187242	1.320662187	1.147537797	1.490087026	0.26645029	0.841239962	47788
Phocaeicola	Cur_vs_DSS	0.481420099	0.476714271	0.297833645	0.614393116	0.737139753	0.847647732	45692
Alistipes	Cur_vs_DSS	1.747576972	1.741484994	1.594608023	1.907883056	0.086804986	0.705338355	39461
Escherichia	Cur_vs_DSS	-5.660647614	-5.636979901	-6.048782923	-5.185965226	0.071561513	0.705338355	34306
Faecalibaculum	Cur_vs_DSS	-1.175167343	-1.173264293	-1.344050609	-1.011012314	0.511064214	0.841239962	24274
Parabacteroides	Cur_vs_DSS	-0.32104743	-0.312981009	-0.498901292	-0.140981934	0.836684658	0.876526784	16673
Lawsonibacter	Cur_vs_DSS	0.382442999	0.372941483	0.210681066	0.521376752	0.636681946	0.847647732	16576
Ligilactobacillus	Cur_vs_DSS	2.19256291	2.187961552	2.013428167	2.366155852	0.04789714	0.626677373	16313
Muribaculum	Cur_vs_DSS	-0.353337073	-0.341139752	-0.521830353	-0.176598616	0.686381437	0.847647732	13905
Akkermansia	Cur_vs_DSS	-0.759277122	-0.73794019	-1.260014213	-0.233537021	0.665376646	0.847647732	11460
Heminiphilus	Cur_vs_DSS	-0.174505822	-0.154235151	-0.370434436	0.122063606	0.904682364	0.904682364	11253
Paraprevotella	Cur_vs_DSS	4.707137305	4.85851768	3.983839472	6.400243554	0.059591611	0.655507726	10096
Lachnoclostridium	Cur_vs_DSS	0.346748725	0.346234918	0.12260993	0.558797648	0.566737851	0.841239962	9496
Unclassified	Cur_vs_DSS	1.022616661	1.023553113	0.838527616	1.210157223	0.03163429	0.626677373	9340
Romboutsia	Cur_vs_DSS	3.597713635	3.795238017	2.841004688	6.042265376	0.103202804	0.705338355	9090

Simiaoa	Cur_vs_DSS	0.651506108	0.645835141	0.304244267	0.970534072	0.687405673	0.847647732	8275
Marseillibacter	Cur_vs_DSS	0.016742659	0.023299524	-0.154662018	0.201427995	0.896627379	0.902487689	7520
Paramuribaculum	Cur_vs_DSS	-0.029758034	-0.043831138	-0.252740297	0.123519635	0.889522276	0.901226516	7407
Lacrimispora	Cur_vs_DSS	-0.256343332	-0.253028251	-0.442025977	-0.051171822	0.767171701	0.852237256	7368
Mediterraneibacter	Cur_vs_DSS	-0.378632655	-0.378653708	-0.580489769	-0.149889855	0.608548338	0.842431118	6960
Parasutterella	Cur_vs_DSS	0.237032667	0.223867786	-0.015469305	0.455314816	0.874211299	0.8975236	6731
Butyricimonas	Cur_vs_DSS	-0.255301799	-0.246403313	-0.428591277	-0.017744009	0.798413598	0.852237256	6433
Acetatifactor	Cur_vs_DSS	-2.751879128	-2.923994009	-4.572486183	-2.023258841	0.236478702	0.841239962	5455
Vallitalea	Cur_vs_DSS	2.339241782	2.335019259	2.049034704	2.582114216	0.033975599	0.626677373	5406
Mucispirillum	Cur_vs_DSS	-2.053778113	-2.043052695	-2.249128583	-1.815056089	0.010235767	0.548536971	5329
Clostridium	Cur_vs_DSS	0.49477131	0.479130437	0.18189454	0.741646182	0.618147508	0.842431118	5301
Bifidobacterium	Cur_vs_DSS	-5.861977662	-6.058788215	-8.682880664	-4.69090054	0.052901337	0.626677373	5234
Vescimonas	Cur_vs_DSS	-1.040797433	-1.044594197	-1.229069927	-0.855943953	0.158293908	0.761789433	5154
Intestinimonas	Cur_vs_DSS	-0.368856506	-0.361831765	-0.573564652	-0.171169265	0.173190682	0.769102101	4998
Desulfovibrio	Cur_vs_DSS	-0.056364207	-0.066629336	-0.293515406	0.108994243	0.849340175	0.882747118	4468
Flintibacter	Cur_vs_DSS	-0.915726062	-0.921863262	-1.155552013	-0.730005586	0.051210413	0.626677373	4382
Ruminiclostridium	Cur_vs_DSS	1.940777753	1.941426507	1.651813139	2.198966734	0.148172933	0.736084891	4283
Ruminococcus	Cur_vs_DSS	7.913240077	8.118574264	6.415869939	10.79010395	0.010685785	0.548536971	4256
Mammaliicoccus	Cur_vs_DSS	1.04500108	1.122073001	0.278351581	2.511014982	0.488296899	0.841239962	3810
Anaerotignum	Cur_vs_DSS	-0.503622383	-0.497775849	-0.802537301	-0.218411238	0.676581377	0.847647732	3414
Sangeribacter	Cur_vs_DSS	-0.23718621	-0.232186213	-0.464763907	0.056177588	0.787811188	0.852237256	3235
Neglectibacter	Cur_vs_DSS	-0.712284501	-0.707783916	-1.016027317	-0.423051415	0.449661788	0.841239962	2840
Butyrivibrio	Cur_vs_DSS	0.110900756	0.154040676	-2.29560436	2.940365237	0.795971551	0.852237256	2593
Anaeromicropila	Cur_vs_DSS	0.516363786	0.523142523	0.135493787	0.909598544	0.579035299	0.841239962	2464
Christensenella	Cur_vs_DSS	1.523007629	1.588579885	0.743687535	2.86742217	0.39956182	0.841239962	2461
Barnesiella	Cur_vs_DSS	0.289090897	0.292906931	-1.208940255	1.995746327	0.802431182	0.852237256	2281
Oscillibacter	Cur_vs_DSS	-0.772235482	-0.7789589	-1.118755187	-0.53410046	0.33409287	0.841239962	2266
Guopingia	Cur_vs_DSS	3.047199411	3.04756949	2.589316507	3.539215267	0.039069012	0.626677373	1993
Staphylococcus	Cur_vs_DSS	1.577920129	1.630608674	1.080327315	2.336550922	0.292765332	0.841239962	1854

Limosilactobacillus	Cur_vs_DSS	0.521397528	0.631959381	-1.199500443	2.442020631	0.784354549	0.852237256	1723
Catenibacillus	Cur_vs_DSS	1.242205336	1.389769119	-0.63994755	3.821840509	0.66103645	0.847647732	1660
Dehalobacterium	Cur_vs_DSS	0.018984601	-0.001513108	-0.330750453	0.362737279	0.854086497	0.882747118	1435
Allobaculum	Cur_vs_DSS	-8.846563639	-8.932968766	-12.13783187	-6.590200534	0.005182427	0.548536971	1327
Angelakisella	Cur_vs_DSS	0.28751623	0.124222425	-1.424726625	1.144925538	0.79329423	0.852237256	1233
Pseudoflavonifractor	Cur_vs_DSS	-2.039385807	-2.047883519	-2.420131066	-1.689372263	0.085908936	0.705338355	1222
Acetivibrio	Cur_vs_DSS	1.566009397	1.687639138	0.598432754	3.317727727	0.3951925	0.841239962	1218
Butyricicoccus	Cur_vs_DSS	0.512080031	0.529861756	0.21159001	0.891413357	0.517514146	0.841239962	1156
Marasmitruncus	Cur_vs_DSS	-2.752407895	-2.865073782	-4.124719095	-1.900047859	0.147049241	0.736084891	1082
Feifania	Cur_vs_DSS	0.506277889	0.513881606	0.134986859	0.854867786	0.480052987	0.841239962	1001
Roseburia	Cur_vs_DSS	-1.70842737	-1.616451303	-4.053261457	0.788724171	0.574917297	0.841239962	994
Peptococcus	Cur_vs_DSS	0.455848282	0.441507873	-0.100140898	1.170633321	0.721892816	0.847647732	986
Vampirovibrio	Cur_vs_DSS	2.747622658	2.904913087	1.956438424	5.712219523	0.102156394	0.705338355	916
Luoshenia	Cur_vs_DSS	1.493863541	1.511028137	1.002159001	2.068868462	0.125842372	0.717767605	912
Muricomes	Cur_vs_DSS	-3.057400029	-3.202757387	-5.130735048	-2.22959629	0.105342741	0.705338355	910
Aestuariispira	Cur_vs_DSS	2.613172802	2.530116079	0.557437396	4.996101707	0.370841577	0.841239962	906
Qiania	Cur_vs_DSS	-0.424196265	-0.283685717	-1.278966511	0.864976146	0.741936219	0.847647732	903
Sporofaciens	Cur_vs_DSS	-2.644226672	-2.682056438	-4.489642028	-0.406168911	0.353176369	0.841239962	872
Fusimonas	Cur_vs_DSS	-0.178732278	-0.202403386	-1.706311057	1.677017432	0.79922587	0.852237256	856
Bilophila	Cur_vs_DSS	-2.634034843	-2.77295675	-5.189808747	-1.354416697	0.316387924	0.841239962	835
Petroclostridium	Cur_vs_DSS	6.263684441	6.334582188	4.329205833	9.156959041	0.017502884	0.626677373	828
Harryflintia	Cur_vs_DSS	-1.253371647	-1.248353854	-1.898852868	-0.689573405	0.313401409	0.841239962	816
Gracilibacter	Cur_vs_DSS	3.095614362	3.247369799	2.181676062	5.079360562	0.124991367	0.717767605	810
Petrocella	Cur_vs_DSS	1.598861963	1.599471885	-1.001411752	3.852997577	0.585652151	0.842431118	805
Leptogranulimonas	Cur_vs_DSS	-3.602427591	-3.80216655	-6.34481022	-2.317864988	0.174795932	0.769102101	709
Coprococcus	Cur_vs_DSS	-0.326392172	-0.322322229	-0.781167668	0.135419718	0.753334181	0.847647732	683
Anaerotruncus	Cur_vs_DSS	-0.562980081	-0.522532725	-1.574618064	0.847395361	0.664346062	0.847647732	643
Faecalimonas	Cur_vs_DSS	1.641555387	1.767883684	0.826766621	3.428603506	0.251302533	0.841239962	639
Congzhengia	Cur_vs_DSS	2.544841718	2.6328391	1.620346301	3.998610457	0.141562018	0.736084891	630

Eubacterium	Cur_vs_DSS	0.816068017	0.792001976	0.07181378	1.477664319	0.50899988	0.841239962	587
Marvinbryantia	Cur_vs_DSS	0.470793925	0.462333077	-1.535078792	2.237137741	0.692097624	0.847647732	574
Odoribacter	Cur_vs_DSS	1.893897219	1.91867935	-0.121845913	3.703889278	0.430705312	0.841239962	566
Enterococcus	Cur_vs_DSS	-2.668871851	-2.589701909	-5.376069331	0.372523599	0.48394746	0.841239962	547
Rhodospirillum	Cur_vs_DSS	0.132449736	0.16218764	-1.648682421	2.449442242	0.821737707	0.866764431	544
Adlercreutzia	Cur_vs_DSS	-0.511566507	-0.537452002	-0.933807268	-0.186294517	0.530573008	0.841239962	524
Anaerocolumna	Cur_vs_DSS	-4.14707135	-4.208708734	-6.226157697	-2.376455486	0.087816302	0.705338355	523
Ruthenibacterium	Cur_vs_DSS	-0.287861024	-0.284138034	-0.875011563	0.271030125	0.723080993	0.847647732	523
Streptococcus	Cur_vs_DSS	-2.064596004	-2.133614829	-3.83395352	-1.084580042	0.27709018	0.841239962	511
Dorea	Cur_vs_DSS	1.116138107	1.24571921	0.361610117	2.7942404	0.449500916	0.841239962	496
Faecalicatena	Cur_vs_DSS	0.69756601	0.643367686	-1.769316863	3.071358941	0.720217915	0.847647732	479
Saccharofermentans	Cur_vs_DSS	0.068818802	0.058052042	-2.571420873	2.38106782	0.714552989	0.847647732	462
Waltera	Cur_vs_DSS	-0.797790774	-0.720814255	-2.852519928	1.675771312	0.704336835	0.847647732	441
Thomasclavelia	Cur_vs_DSS	4.108009417	4.202626099	1.636962014	7.019652677	0.167878055	0.769102101	425
Jeotgalicoccus	Cur_vs_DSS	1.562910824	1.525560195	-1.72239109	4.56456754	0.471549208	0.841239962	421
Eisenbergiella	Cur_vs_DSS	-2.888398722	-2.982140214	-5.219565646	-1.162288244	0.29448719	0.841239962	418
Merdimmobilis	Cur_vs_DSS	5.770352896	5.794230564	3.528734826	8.71938756	0.052696729	0.626677373	345
Anaeroplasma	Cur_vs_DSS	0.303102999	0.295807426	-1.813285078	3.440073429	0.74395147	0.847647732	327
Variimorphobacter	Cur_vs_DSS	0.156981402	0.301308015	-1.360696515	2.655542528	0.754076229	0.847647732	325
Natranaerovirga	Cur_vs_DSS	1.281096219	1.243872113	-1.010076416	3.173045742	0.5497241	0.841239962	313
Amedibacillus	Cur_vs_DSS	-1.761540435	-1.910827979	-3.402672172	-1.047151973	0.27563793	0.841239962	298
Fumia	Cur_vs_DSS	-2.470685705	-2.604047017	-3.983897238	-1.544496847	0.140655227	0.736084891	297
Lentihominibacter	Cur_vs_DSS	2.829493199	3.045796343	1.614772914	5.150459724	0.080895543	0.705338355	281
Corynebacterium	Cur_vs_DSS	-1.707646325	-1.739561535	-4.683998189	0.510080288	0.497860335	0.841239962	269
Dysosmobacter	Cur_vs_DSS	0.160844162	0.296546151	-0.869073546	2.147523951	0.698498607	0.847647732	267
Anaerobium	Cur_vs_DSS	-1.285094746	-1.318189635	-3.464260748	0.767824742	0.558051313	0.841239962	255
Lactobacillus	Cur_vs_DSS	-0.809489908	-0.918066285	-3.760737746	1.56036671	0.690397671	0.847647732	247
Sinanaerobacter	Cur_vs_DSS	-0.167178473	-0.031415677	-1.35663219	1.645267068	0.691690659	0.847647732	235
Sporobacter	Cur_vs_DSS	-0.159062331	-0.077331798	-1.365125642	1.607871762	0.662429453	0.847647732	229

Ileibacterium	Cur_vs_DSS	-6.316905709	-6.333773018	-9.381681836	-3.700400637	0.049037328	0.626677373	228
Ihubacter	Cur_vs_DSS	0.791541469	1.064420934	-0.846199875	3.876574365	0.612972767	0.842431118	192
Enterocloster	Cur_vs_DSS	-3.210211841	-3.302182345	-6.188678334	-0.70497704	0.201114755	0.841239962	186
Abyssivirga	Cur_vs_DSS	0.905311877	0.965980341	-0.668969092	2.947530148	0.577337179	0.841239962	176
Acutalibacter	Cur_vs_DSS	2.469064077	2.50965748	-0.228140041	5.667399566	0.442117589	0.841239962	176
Xiamenia	Cur_vs_DSS	-0.099220171	-0.076228673	-0.797678759	0.716734192	0.737794186	0.847647732	174
Holdemania	Cur_vs_DSS	1.118173056	1.219616035	-0.886701269	3.647456144	0.470239197	0.841239962	148
Zhenpiania	Cur_vs_DSS	-0.893991728	-0.738055771	-1.912587382	0.551246669	0.577712129	0.841239962	142
Paludihabitans	Cur_vs_DSS	-3.697185005	-3.843186935	-7.294668738	-1.376470012	0.125786406	0.717767605	127
Jingyaoa	Cur_vs_DSS	-2.966526604	-3.052361691	-6.149721799	-0.021242627	0.275410775	0.841239962	124
Evtepia	Cur_vs_DSS	0.525612241	0.52766215	-3.216804479	3.882972107	0.521315677	0.841239962	119
Tannockella	Cur_vs_DSS	0.785605821	0.844821856	-2.507214189	4.415060189	0.551876087	0.841239962	116
Mageeibacillus	Cur_vs_DSS	-1.040252894	-0.977933446	-3.796614209	1.99061225	0.561739518	0.841239962	102
Berryella	Cur_vs_DSS	1.584632521	1.610203236	-1.097330293	4.375828785	0.434239111	0.841239962	95
Oxalobacter	Cur_vs_DSS	3.017182749	3.147575579	1.058055822	5.938316356	0.121602578	0.717767605	94
Rhizobium	Cur_vs_DSS	-0.601401483	-0.557790293	-2.598344019	1.441619111	0.68954983	0.847647732	92
Anaerobacterium	Cur_vs_DSS	2.997371701	3.067072527	0.267189456	6.1872802	0.289191792	0.841239962	89
Breznakia	Cur_vs_DSS	2.448374922	2.7366074	0.367682391	6.57076352	0.32609321	0.841239962	85
Raoultibacter	Cur_vs_DSS	0.508570094	0.653529376	-0.554056047	2.509481927	0.539652545	0.841239962	84
Frisingicoccus	Cur_vs_DSS	-4.524554409	-4.484966684	-7.447050769	-1.78669661	0.098911843	0.705338355	80
Gordonibacter	Cur_vs_DSS	1.316087699	1.363382435	0.332703751	2.786553944	0.262419323	0.841239962	76
Lachnotalea	Cur_vs_DSS	0.270434284	0.28658554	-2.5406231	3.546366027	0.599556218	0.842431118	67
Hungatella	Cur_vs_DSS	-0.221895077	-0.110529006	-3.392941774	3.166379767	0.546241351	0.841239962	56
Ructibacterium	Cur_vs_DSS	-0.288787299	-0.434765484	-4.671241005	3.255818844	0.517391435	0.841239962	55
Diplocloster	Cur_vs_DSS	-0.491728097	-0.652409125	-4.531865937	2.394606622	0.522303876	0.841239962	40
Intestinibacillus	Cur_vs_DSS	-2.430925005	-2.502045043	-5.186460414	-0.048873912	0.30204633	0.841239962	30
Alkalibaculum	Cur_vs_DSS	0.334647004	0.252271111	-2.585593884	3.092513115	0.604442209	0.842431118	25
Longibaculum	Cur_vs_DSS	1.065866148	1.095861986	-2.207270307	5.017732585	0.540457932	0.841239962	25
Monoglobus	Cur_vs_DSS	-1.313568305	-1.156643831	-4.018135792	2.418569305	0.5183785	0.841239962	24

Cuneatibacter	Cur_vs_DSS	-0.46641586	-0.529416262	-4.58812893	3.029917472	0.481556894	0.841239962	21
Solibaculum	Cur_vs_DSS	-0.242209075	-0.273741716	-3.713272494	3.307652556	0.59281318	0.842431118	21
Cupriavidus	Cur_vs_DSS	0.588728832	0.531035795	-3.781992415	4.000519378	0.499533224	0.841239962	20
Gehongia	Cur_vs_DSS	-0.181795685	-0.224493163	-3.899636341	2.98217567	0.541469707	0.841239962	18
Jutongia	Cur_vs_DSS	0.031978697	-0.092564467	-3.257630027	3.124139875	0.52932351	0.841239962	17
Bittarella	Cur_vs_DSS	-1.080710332	-1.191821997	-4.821580113	1.777679492	0.516348646	0.841239962	16
Massiliimalia	Cur_vs_DSS	-1.146996472	-1.129706451	-4.216464625	2.792539587	0.44490862	0.841239962	16
Enteroscipio	Cur_vs_DSS	-3.114352321	-3.164325893	-6.341045	-0.086261607	0.234423498	0.841239962	15
Hydrogeniiclostridium	Cur_vs_DSS	0.698611032	0.621893226	-2.541184805	3.995576237	0.504750811	0.841239962	12
Arabiibacter	Cur_vs_DSS	-0.580646824	-0.33850442	-3.580397171	3.594661709	0.52590774	0.841239962	11
Aminipila	Cur_vs_DSS	-1.617774012	-1.751978238	-5.712067698	1.74466196	0.403899274	0.841239962	9
Victivallis	Cur_vs_DSS	0.8399322	0.628894383	-3.357823372	4.051950798	0.511798089	0.841239962	9
Massilioclostridium	Cur_vs_DSS	1.058723985	1.253833168	-2.274000775	4.502640641	0.502347095	0.841239962	8
Pseudomonas	Cur_vs_DSS	-0.02780527	-0.140423471	-4.163045229	3.181832635	0.510729932	0.841239962	7
Scatolibacter	Cur_vs_DSS	-0.094980032	-0.349445293	-4.616574285	3.192194009	0.513312881	0.841239962	5
Blautia	Cur_vs_DSS	-0.925729587	-0.779371284	-4.717537858	2.870471184	0.48813811	0.841239962	4
Robinsoniella	Cur_vs_DSS	1.36609827	1.177642471	-2.401551502	4.59806073	0.483266672	0.841239962	4
Vagococcus	Cur_vs_DSS	0.751376925	0.650694691	-2.641844903	4.142079135	0.521272859	0.841239962	4
Pusillibacter	Cur_vs_DSS	-0.490051917	-0.349253299	-4.637190915	3.950798287	0.490725552	0.841239962	3
Shigella	Cur_vs_DSS	-0.798492926	-0.993772905	-6.10919238	3.862151825	0.449069361	0.841239962	3
Turicibacter	Stv_vs_DSS	1.297546578	1.302030236	1.122842852	1.504477306	0.382614107	0.850661408	72642
Kineothrix	Stv_vs_DSS	-0.029994965	-0.023726422	-0.183544751	0.156667801	0.92646126	0.92646126	70740
Duncaniella	Stv_vs_DSS	1.584290787	1.583408894	1.440797158	1.77350996	0.088363013	0.850661408	53394
Bacteroides	Stv_vs_DSS	0.619685972	0.62773438	0.458431149	0.827847522	0.536538905	0.850661408	47788
Phocaeicola	Stv_vs_DSS	-0.546542666	-0.539479147	-0.759177704	-0.325592557	0.739509642	0.850661408	45692
Alistipes	Stv_vs_DSS	0.128993065	0.12953244	-0.054865203	0.301218301	0.901214012	0.919118926	39461
Escherichia	Stv_vs_DSS	-2.22655818	-2.236740514	-2.535687854	-1.909189149	0.416579085	0.850661408	34306
Faecalibaculum	Stv_vs_DSS	-3.467359131	-3.465613134	-3.695003664	-3.276733417	0.089307412	0.850661408	24274
Parabacteroides	Stv_vs_DSS	0.2963654	0.297868482	0.11290853	0.477408193	0.84508307	0.891685842	16673

Lawsonibacter	Stv_vs_DSS	0.226756537	0.218727648	0.039625503	0.39880276	0.780181205	0.852112806	16576
Ligilactobacillus	Stv_vs_DSS	0.470032131	0.471850093	0.313374138	0.65642452	0.564752009	0.850661408	16313
Muribaculum	Stv_vs_DSS	0.116132275	0.12328144	-0.042651859	0.316105024	0.884624361	0.914309742	13905
Akkermansia	Stv_vs_DSS	0.126384824	0.145879479	-0.225101159	0.595539414	0.897744266	0.919118926	11460
Heminiphilus	Stv_vs_DSS	-0.85092137	-0.870891482	-1.137692256	-0.636857698	0.573201777	0.850661408	11253
Paraprevotella	Stv_vs_DSS	3.191620558	3.361006861	2.533167548	5.149830271	0.145104293	0.850661408	10096
Lachnoclostridium	Stv_vs_DSS	-0.767702775	-0.765029685	-0.966459509	-0.550679148	0.443268406	0.850661408	9496
Unclassified	Stv_vs_DSS	0.924827922	0.934726822	0.760298251	1.138542574	0.132966656	0.850661408	9340
Romboutsia	Stv_vs_DSS	5.59856923	5.822705898	4.919081014	8.055666648	0.031690858	0.81339868	9090
Simiaoa	Stv_vs_DSS	0.03309667	0.047874248	-0.390030241	0.447158968	0.923082371	0.92646126	8275
Marseillibacter	Stv_vs_DSS	-0.485098172	-0.481148243	-0.714077763	-0.228713282	0.582883264	0.850661408	7520
Paramuribaculum	Stv_vs_DSS	-0.052564521	-0.058346936	-0.290450752	0.200062967	0.926234979	0.92646126	7407
Lacrimispora	Stv_vs_DSS	1.343220159	1.351715584	1.098330634	1.571422475	0.161332309	0.850661408	7368
Mediterraneibacter	Stv_vs_DSS	-0.80208362	-0.805987715	-1.000500618	-0.568646501	0.329842373	0.850661408	6960
Parasutterella	Stv_vs_DSS	0.416985019	0.414505197	0.177342951	0.648926383	0.750435294	0.850661408	6731
Butyricimonas	Stv_vs_DSS	0.16631456	0.170027938	-0.031524816	0.390231976	0.851154667	0.891685842	6433
Acetatifactor	Stv_vs_DSS	-0.515325577	-0.508190793	-0.775995352	-0.213816924	0.68656014	0.850661408	5455
Vallitalea	Stv_vs_DSS	1.356059792	1.350506197	1.116454931	1.588179536	0.173548495	0.850661408	5406
Mucispirillum	Stv_vs_DSS	-1.734401014	-1.715361537	-1.979743702	-1.441676368	0.225701707	0.850661408	5329
Clostridium	Stv_vs_DSS	0.91557262	0.918315986	0.691607684	1.220237925	0.343312711	0.850661408	5301
Bifidobacterium	Stv_vs_DSS	-3.751835853	-3.746731675	-4.159030117	-3.3175919	0.067243599	0.850661408	5234
Vescimonas	Stv_vs_DSS	-1.033926755	-1.02703031	-1.255457071	-0.76884666	0.209842683	0.850661408	5154
Intestinimonas	Stv_vs_DSS	-0.643470664	-0.639428076	-0.853296344	-0.42058453	0.287716428	0.850661408	4998
Desulfovibrio	Stv_vs_DSS	-0.297209219	-0.294750485	-0.483268472	-0.068037564	0.621736076	0.850661408	4468
Flintibacter	Stv_vs_DSS	-1.150772394	-1.161855313	-1.424356892	-0.965038328	0.095892159	0.850661408	4382
Ruminiclostridium	Stv_vs_DSS	0.557035243	0.575023084	0.34169808	0.877129336	0.641012432	0.850661408	4283
Ruminococcus	Stv_vs_DSS	8.058592153	8.227194557	6.53040384	10.93835233	0.008722589	0.67163939	4256
Mammaliicoccus	Stv_vs_DSS	2.117652275	2.163924816	1.273745443	3.413806763	0.359618061	0.850661408	3810
Anaerotignum	Stv_vs_DSS	-0.206468445	-0.21033775	-0.473497835	0.063680463	0.848267887	0.891685842	3414

Sangeribacter	Stv_vs_DSS	0.21358595	0.234728352	0.022910932	0.463270174	0.797189547	0.852549932	3235
Neglectibacter	Stv_vs_DSS	-0.957934719	-0.93607636	-1.313762696	-0.546566545	0.378462192	0.850661408	2840
Butyrivibrio	Stv_vs_DSS	-1.052701729	-1.204166235	-4.155206519	0.899922491	0.727071483	0.850661408	2593
Anaeromicropila	Stv_vs_DSS	-0.071794542	-0.070641617	-0.414245801	0.345779561	0.877774643	0.913360101	2464
Christensenella	Stv_vs_DSS	1.319153985	1.366725879	0.476455635	2.723967389	0.487103085	0.850661408	2461
Barnesiella	Stv_vs_DSS	0.865053586	0.850964667	-0.765544116	2.565331242	0.749063941	0.850661408	2281
Oscillibacter	Stv_vs_DSS	-1.495324096	-1.492004842	-1.753270743	-1.140776014	0.209701652	0.850661408	2266
Guopingia	Stv_vs_DSS	1.987237466	1.985940183	1.470522596	2.46170964	0.167318193	0.850661408	1993
Staphylococcus	Stv_vs_DSS	0.855644577	0.873079312	-0.03473768	1.676514688	0.686431246	0.850661408	1854
Limosilactobacillus	Stv_vs_DSS	-1.78567074	-1.695051815	-3.771602589	0.459252975	0.606503996	0.850661408	1723
Catenibacillus	Stv_vs_DSS	2.693670713	2.860470068	1.346877514	5.080419283	0.356417536	0.850661408	1660
Dehalobacterium	Stv_vs_DSS	0.297842969	0.295194609	0.002669796	0.587247473	0.684728511	0.850661408	1435
Allobaculum	Stv_vs_DSS	-8.641403794	-8.944555318	-12.53655455	-6.787809078	0.004398643	0.67163939	1327
Angelakisella	Stv_vs_DSS	-3.10773653	-3.251410801	-5.592326886	-1.730044716	0.199509223	0.850661408	1233
Pseudoflavonifractor	Stv_vs_DSS	-1.912897491	-1.915233517	-2.229596828	-1.612145766	0.019160757	0.73768913	1222
Acetivibrio	Stv_vs_DSS	1.74859901	1.845813476	0.766445315	3.552771602	0.392806693	0.850661408	1218
Butyricicoccus	Stv_vs_DSS	-0.275510217	-0.277476133	-0.627148091	0.114209376	0.680431623	0.850661408	1156
Marasmitruncus	Stv_vs_DSS	-0.891123681	-0.923441342	-1.360111049	-0.52719117	0.463076473	0.850661408	1082
Feifania	Stv_vs_DSS	0.235564807	0.245225014	-0.134046561	0.659119863	0.763646499	0.850661408	1001
Roseburia	Stv_vs_DSS	-1.176611002	-1.188400986	-3.852604321	1.39577075	0.637267925	0.850661408	994
Peptococcus	Stv_vs_DSS	0.183152003	0.207282097	-0.214746608	0.64877076	0.745514026	0.850661408	986
Vampirovibrio	Stv_vs_DSS	2.028822156	2.205828588	1.211711152	4.7334965	0.192816183	0.850661408	916
Luoshenia	Stv_vs_DSS	0.177411713	0.03845653	-1.357066363	0.942494824	0.767455146	0.850661408	912
Muricomes	Stv_vs_DSS	-0.712618134	-0.721075049	-1.138694909	-0.324949337	0.463733378	0.850661408	910
Aestuariispira	Stv_vs_DSS	-1.042661812	-0.968680721	-3.272853204	1.831771099	0.661766327	0.850661408	906
Qiania	Stv_vs_DSS	-1.00759035	-1.001613212	-2.601179093	0.516559781	0.664473724	0.850661408	903
Sporofaciens	Stv_vs_DSS	-0.32661785	-0.362111597	-2.131228302	1.087602033	0.773328553	0.850661408	872
Fusimonas	Stv_vs_DSS	1.785869683	1.893271212	0.846424946	3.653947182	0.307390483	0.850661408	856
Bilophila	Stv_vs_DSS	1.023431902	1.066233658	0.568497064	1.907008927	0.498065107	0.850661408	835

Petroclostridium	Stv_vs_DSS	6.420852276	6.614066359	4.51835591	9.290361437	0.01458836	0.73768913	828
Harryflintia	Stv_vs_DSS	-1.371332959	-1.47580714	-2.945493052	-0.634784578	0.42562848	0.850661408	816
Gracilibacter	Stv_vs_DSS	1.209363781	1.234011998	-0.572029785	3.083831525	0.613364921	0.850661408	810
Petrocella	Stv_vs_DSS	-0.451697407	-0.479496608	-2.785197859	2.389138804	0.728923168	0.850661408	805
Leptogranulimonas	Stv_vs_DSS	-6.842192785	-6.951114044	-9.361913238	-4.922274372	0.026563477	0.81339868	709
Coprococcus	Stv_vs_DSS	-0.154836516	-0.146830628	-0.600820273	0.262877291	0.786957681	0.852549932	683
Anaerotruncus	Stv_vs_DSS	1.272999922	1.428989571	0.474331643	2.86720942	0.380184554	0.850661408	643
Faecalimonas	Stv_vs_DSS	0.928514889	1.106442474	0.112679586	2.495891888	0.49817254	0.850661408	639
Congzhengia	Stv_vs_DSS	1.242129336	1.383788952	0.329028688	3.228070534	0.462224826	0.850661408	630
Eubacterium	Stv_vs_DSS	0.111553005	0.00637017	-1.616138567	1.039660237	0.792449116	0.852549932	587
Marvinbryantia	Stv_vs_DSS	1.366679519	1.263291119	-0.762373944	2.967326252	0.529062757	0.850661408	574
Odoribacter	Stv_vs_DSS	1.073738413	0.986285182	-1.536381767	2.933887932	0.688543097	0.850661408	566
Enterococcus	Stv_vs_DSS	-0.972432646	-0.98152033	-3.61159011	2.212035541	0.701011986	0.850661408	547
Rhodospirillum	Stv_vs_DSS	0.548996506	0.559778213	-1.501993313	2.888901423	0.771123633	0.850661408	544
Adlercreutzia	Stv_vs_DSS	-1.168838656	-1.181924156	-1.597192136	-0.710311233	0.23833136	0.850661408	524
Anaerocolumna	Stv_vs_DSS	0.474402819	0.458716277	-0.184248505	1.191479721	0.649822597	0.850661408	523
Ruthenibacterium	Stv_vs_DSS	-1.730451837	-1.851260159	-3.354460449	-0.647426697	0.356659138	0.850661408	523
Streptococcus	Stv_vs_DSS	0.477378162	0.455902724	-0.055361408	0.93751326	0.75826162	0.850661408	511
Dorea	Stv_vs_DSS	0.366984919	0.437930926	-1.040142098	2.324231865	0.759014568	0.850661408	496
Faecalicatena	Stv_vs_DSS	0.145972638	0.105542694	-2.955559556	3.035853049	0.734546573	0.850661408	479
Saccharofermentans	Stv_vs_DSS	0.673415232	0.745141108	-2.172638729	3.135735288	0.692647644	0.850661408	462
Waltera	Stv_vs_DSS	-0.068357951	-0.018490089	-2.542227851	2.3747611	0.767666208	0.850661408	441
Thomasclavelia	Stv_vs_DSS	3.364626257	3.392613779	1.023555953	6.252518062	0.231594029	0.850661408	425
Jeotgalicoccus	Stv_vs_DSS	1.745308744	1.889819482	-1.042571722	5.097968329	0.551483902	0.850661408	421
Eisenbergiella	Stv_vs_DSS	-0.620755198	-0.631452246	-2.337305297	1.372483334	0.695174141	0.850661408	418
Merdimmobilis	Stv_vs_DSS	4.423220099	4.474035218	2.023196356	7.774965421	0.113546558	0.850661408	345
Anaeroplasma	Stv_vs_DSS	-0.442819686	-0.418443545	-3.14358754	1.867034557	0.712158528	0.850661408	327
Variimorphobacter	Stv_vs_DSS	-3.490240133	-3.489253329	-6.305005601	-0.87861958	0.261879759	0.850661408	325
Natranaerovirga	Stv_vs_DSS	1.067985753	0.996663242	-1.35988607	2.86096441	0.57542052	0.850661408	313

Amedibacillus	Stv_vs_DSS	-1.368029132	-1.371798924	-1.958053734	-0.768144502	0.292359988	0.850661408	298
Fumia	Stv_vs_DSS	-2.512950717	-2.604446703	-4.290037343	-1.442612428	0.116437973	0.850661408	297
Lentihominibacter	Stv_vs_DSS	2.013736039	2.153225292	0.548377927	4.239376178	0.248128719	0.850661408	281
Corynebacterium	Stv_vs_DSS	-0.129647094	0.023897068	-1.094254749	1.620191368	0.721688067	0.850661408	269
Dysosmobacter	Stv_vs_DSS	-2.787974487	-2.808806228	-4.974338337	-0.39252362	0.279561127	0.850661408	267
Anaerobium	Stv_vs_DSS	-0.694805981	-0.708788565	-2.632533346	1.577293199	0.651450675	0.850661408	255
Lactobacillus	Stv_vs_DSS	-2.497626324	-2.709162295	-6.11984947	-0.007132769	0.372673281	0.850661408	247
Sinanaerobacter	Stv_vs_DSS	-0.699338467	-0.585225174	-2.376620141	1.625652588	0.639333999	0.850661408	235
Sporobacter	Stv_vs_DSS	0.498201013	0.590134036	-0.578196091	2.066876714	0.662677853	0.850661408	229
Ileibacterium	Stv_vs_DSS	-6.584343592	-6.609441412	-9.445242637	-4.105079126	0.03876881	0.850661408	228
Ihubacter	Stv_vs_DSS	1.733139647	1.927101384	0.305487781	4.71128794	0.3575902	0.850661408	192
Enterocloster	Stv_vs_DSS	0.515483016	0.683700274	-0.395374596	2.352483835	0.643604485	0.850661408	186
Abyssivirga	Stv_vs_DSS	0.068959986	-0.026872992	-2.263825551	2.082121504	0.691353229	0.850661408	176
Acutalibacter	Stv_vs_DSS	-0.421338518	-0.468662044	-4.464085427	2.804511004	0.607843924	0.850661408	176
Xiamenia	Stv_vs_DSS	-1.743688854	-1.912409186	-4.275883147	-0.391257966	0.420974051	0.850661408	174
Holdemania	Stv_vs_DSS	1.419297744	1.581695686	0.385362281	3.563087896	0.253695017	0.850661408	148
Zhenpiania	Stv_vs_DSS	-0.051484694	0.05588276	-0.945637652	1.140515327	0.76465513	0.850661408	142
Paludihabitans	Stv_vs_DSS	-1.699669108	-1.802045062	-3.995031596	0.401611086	0.416758799	0.850661408	127
Jingyaoa	Stv_vs_DSS	-1.992164642	-1.863408773	-4.498217832	1.021285873	0.456628341	0.850661408	124
Evtepia	Stv_vs_DSS	2.777778029	2.752199585	-0.824481691	6.118045837	0.412661702	0.850661408	119
Tannockella	Stv_vs_DSS	4.31892369	4.444262894	2.267159426	7.501076459	0.114909615	0.850661408	116
Mageeibacillus	Stv_vs_DSS	-0.668299415	-0.608645946	-2.915496865	2.200596102	0.637273871	0.850661408	102
Berryella	Stv_vs_DSS	2.993679851	3.200554752	1.110700407	6.098212829	0.104525619	0.850661408	95
Oxalobacter	Stv_vs_DSS	2.250359136	2.234453546	-0.971141773	5.370112205	0.311170676	0.850661408	94
Rhizobium	Stv_vs_DSS	0.933220093	1.089881555	-0.502431255	3.33544689	0.639043206	0.850661408	92
Anaerobacterium	Stv_vs_DSS	-0.353868898	-0.337829991	-3.411819007	2.803588082	0.598906451	0.850661408	89
Breznakia	Stv_vs_DSS	-0.695945243	-0.720194863	-4.038163872	2.488356505	0.519908297	0.850661408	85
Raoultibacter	Stv_vs_DSS	-0.44352197	-0.501489678	-2.267773698	1.561403401	0.582234824	0.850661408	84
Frisingicoccus	Stv_vs_DSS	-1.182782485	-1.279378614	-3.84392025	1.211872927	0.508348426	0.850661408	80

Gordonibacter	Stv_vs_DSS	-3.298074363	-3.413207638	-6.559051872	-1.008815137	0.162608746	0.850661408	76
Lachnotalea	Stv_vs_DSS	0.499383867	0.620505147	-1.824441363	3.80695746	0.625598438	0.850661408	67
Hungatella	Stv_vs_DSS	0.071252489	0.173757627	-3.594742696	3.367478662	0.600480388	0.850661408	56
Ructibacterium	Stv_vs_DSS	-0.961135787	-0.720649982	-4.892625097	2.962593466	0.502735516	0.850661408	55
Diplocloster	Stv_vs_DSS	0.804749247	0.827972614	-2.578708191	4.217235111	0.537911725	0.850661408	40
Intestinibacillus	Stv_vs_DSS	-3.329814018	-3.41810963	-6.914540138	-0.501675393	0.156163989	0.850661408	30
Alkalibaculum	Stv_vs_DSS	0.625417936	0.527014058	-3.032092251	3.711618316	0.594959401	0.850661408	25
Longibaculum	Stv_vs_DSS	0.042636308	0.049223162	-4.043425722	4.026366047	0.579561811	0.850661408	25
Monoglobus	Stv_vs_DSS	-0.67888237	-0.677165185	-3.503859372	2.525727932	0.551325071	0.850661408	24
Cuneatibacter	Stv_vs_DSS	1.783126102	1.98400306	-1.505732191	5.886258808	0.415142269	0.850661408	21
Solibaculum	Stv_vs_DSS	-1.26400634	-1.259583531	-4.64972866	1.754362002	0.461100582	0.850661408	21
Cupriavidus	Stv_vs_DSS	1.603568006	1.603546069	-1.946830916	5.426834765	0.478835396	0.850661408	20
Gehongia	Stv_vs_DSS	0.081671999	0.030479297	-3.549127762	3.939628172	0.600194938	0.850661408	18
Jutongia	Stv_vs_DSS	1.547314441	1.292861122	-2.402108103	4.148908734	0.439018933	0.850661408	17
Bittarella	Stv_vs_DSS	-2.0783568	-2.128806355	-5.18145127	1.544016332	0.351314351	0.850661408	16
Massiliimalia	Stv_vs_DSS	-0.013153966	0.093105091	-3.335607583	3.874814468	0.604361642	0.850661408	16
Enteroscipio	Stv_vs_DSS	-1.803553718	-1.859506568	-5.4822469	0.520133697	0.418399778	0.850661408	15
Hydrogeniiclostridium	Stv_vs_DSS	-0.314927402	-0.457226867	-4.272597284	3.053444083	0.512746939	0.850661408	12
Arabiibacter	Stv_vs_DSS	1.419075814	1.458305746	-1.852978185	5.468800868	0.485554627	0.850661408	11
Aminipila	Stv_vs_DSS	-0.861708785	-0.725677844	-3.919627911	3.194409897	0.522942625	0.850661408	9
Victivallis	Stv_vs_DSS	-0.570613164	-0.662374293	-4.909524001	2.860627573	0.490619618	0.850661408	9
Massilioclostridium	Stv_vs_DSS	0.365503907	0.473654527	-2.783025376	4.281223782	0.525402326	0.850661408	8
Pseudomonas	Stv_vs_DSS	0.402748322	0.565110637	-3.104213217	4.220942193	0.527175962	0.850661408	7
Scatolibacter	Stv_vs_DSS	-0.42540988	-0.471113227	-3.145635299	2.590880203	0.567313147	0.850661408	5
Blautia	Stv_vs_DSS	-0.297866283	-0.273377474	-3.60515587	2.955151465	0.589647114	0.850661408	4
Robinsoniella	Stv_vs_DSS	-0.474543192	-0.515099055	-4.529954732	3.245103756	0.533076677	0.850661408	4
Vagococcus	Stv_vs_DSS	0.603264957	0.326263221	-3.659592882	3.329756148	0.490746085	0.850661408	4
Pusillibacter	Stv_vs_DSS	0.144207538	0.220438778	-3.96072094	4.080727011	0.494115714	0.850661408	3
Shigella	Stv_vs_DSS	-0.209101485	-0.100987269	-3.636878786	3.643794487	0.516725704	0.850661408	3

Turicibacter	Cur_vs_TSP	-0.450587141	-0.452988959	-0.622459483	-0.294564321	0.257416689	0.83521047	72642
Kineothrix	Cur_vs_TSP	0.29282363	0.294352369	0.113651666	0.482131285	0.693762325	0.83521047	70740
Duncaniella	Cur_vs_TSP	0.442032733	0.442934047	0.276667021	0.619366958	0.303863072	0.83521047	53394
Bacteroides	Cur_vs_TSP	-0.046192498	-0.049860778	-0.233470776	0.112089645	0.917102997	0.923097134	47788
Phocaeicola	Cur_vs_TSP	-0.487759293	-0.488056043	-0.660798988	-0.292095515	0.697330845	0.83521047	45692
Alistipes	Cur_vs_TSP	1.645732935	1.65424765	1.46473101	1.826989575	0.059549842	0.83521047	39461
Escherichia	Cur_vs_TSP	-3.871158234	-3.850731046	-4.216689825	-3.421576933	0.042002774	0.83521047	34306
Faecalibaculum	Cur_vs_TSP	0.794584335	0.79466081	0.588125399	1.003443995	0.597829679	0.83521047	24274
Parabacteroides	Cur_vs_TSP	-0.326710718	-0.318617466	-0.472073474	-0.117743458	0.660381224	0.83521047	16673
Lawsonibacter	Cur_vs_TSP	0.96744519	0.97130166	0.787441467	1.15189837	0.119815853	0.83521047	16576
Ligilactobacillus	Cur_vs_TSP	2.480188427	2.483898946	2.314128844	2.669986523	0.033464952	0.83521047	16313
Muribaculum	Cur_vs_TSP	0.597947476	0.605003572	0.424390945	0.811342133	0.428928437	0.83521047	13905
Akkermansia	Cur_vs_TSP	-4.651158302	-4.648855657	-5.029933086	-4.274164062	0.047391125	0.83521047	11460
Heminiphilus	Cur_vs_TSP	0.030256936	0.021598861	-0.269171618	0.255570844	0.948343635	0.948343635	11253
Paraprevotella	Cur_vs_TSP	-1.947127316	-1.950824278	-2.267055445	-1.69104853	0.200441826	0.83521047	10096
Lachnoclostridium	Cur_vs_TSP	-0.529654759	-0.539081979	-0.744050045	-0.370429234	0.342202089	0.83521047	9496
Unclassified	Cur_vs_TSP	0.897158921	0.888538766	0.716166747	1.044128531	0.064939198	0.83521047	9340
Romboutsia	Cur_vs_TSP	5.818501032	5.953660008	4.331606879	8.437689286	0.057076434	0.83521047	9090
Simiaoa	Cur_vs_TSP	-0.631732565	-0.6268561	-0.857042691	-0.404632284	0.599079095	0.83521047	8275
Marseillibacter	Cur_vs_TSP	0.570891444	0.562060179	0.329604888	0.774004533	0.349881952	0.83521047	7520
Paramuribaculum	Cur_vs_TSP	-1.35191772	-1.359588569	-1.579008555	-1.121755018	0.038853244	0.83521047	7407
Lacrimispora	Cur_vs_TSP	-0.730609642	-0.717475652	-0.930645599	-0.462011025	0.315651674	0.83521047	7368
Mediterraneibacter	Cur_vs_TSP	-0.24503332	-0.242328514	-0.436483575	-0.038413788	0.415724679	0.83521047	6960
Parasutterella	Cur_vs_TSP	-0.318566888	-0.313558144	-0.516470072	-0.105163385	0.717846985	0.83521047	6731
Butyricimonas	Cur_vs_TSP	-0.760567061	-0.771354031	-0.996502374	-0.571399869	0.239255579	0.83521047	6433
Acetatifactor	Cur_vs_TSP	-2.415443904	-2.568016026	-4.162670119	-1.753567356	0.264725003	0.83521047	5455
Vallitalea	Cur_vs_TSP	1.617909727	1.610651488	1.392355806	1.848104128	0.104927247	0.83521047	5406
Mucispirillum	Cur_vs_TSP	-0.177910903	-0.182699131	-0.421855188	0.055058381	0.768932488	0.83521047	5329
Clostridium	Cur_vs_TSP	-0.081195436	-0.088542933	-0.342148143	0.100884079	0.878110832	0.891227839	5301

Bifidobacterium	Cur_vs_TSP	-4.362681931	-4.621319963	-7.255795336	-3.196266884	0.124513883	0.83521047	5234
Vescimonas	Cur_vs_TSP	-0.062432501	-0.045534619	-0.27479555	0.204633142	0.879653451	0.891227839	5154
Intestinimonas	Cur_vs_TSP	0.45007735	0.452242433	0.227352314	0.633701953	0.160553458	0.83521047	4998
Desulfovibrio	Cur_vs_TSP	0.282857402	0.282944598	0.02341951	0.530792371	0.708557989	0.83521047	4468
Flintibacter	Cur_vs_TSP	-0.00335676	0.005787127	-0.2283231	0.238038665	0.799862028	0.83521047	4382
Ruminiclostridium	Cur_vs_TSP	1.58760117	1.596384207	1.37113123	1.834142363	0.072293925	0.83521047	4283
Ruminococcus	Cur_vs_TSP	0.516500119	0.514136466	0.313259923	0.72531586	0.370228195	0.83521047	4256
Mammaliicoccus	Cur_vs_TSP	-0.729596712	-0.735528183	-1.101157025	-0.311717939	0.491724168	0.83521047	3810
Anaerotignum	Cur_vs_TSP	-0.614445155	-0.608053712	-0.900015928	-0.354279072	0.527146583	0.83521047	3414
Sangeribacter	Cur_vs_TSP	0.205368563	0.20842765	-0.015070964	0.449521668	0.599611022	0.83521047	3235
Neglectibacter	Cur_vs_TSP	-0.305464944	-0.295262027	-0.519945346	-0.035925219	0.738639151	0.83521047	2840
Butyrivibrio	Cur_vs_TSP	0.024505275	0.061492599	-2.604298401	2.400339492	0.798521533	0.83521047	2593
Anaeromicropila	Cur_vs_TSP	1.153859786	1.160731575	0.890537962	1.441669597	0.141715082	0.83521047	2464
Christensenella	Cur_vs_TSP	-0.773514042	-0.780606065	-1.041515199	-0.529757058	0.336186233	0.83521047	2461
Barnesiella	Cur_vs_TSP	2.716088474	2.824995523	0.339675686	5.82508879	0.441033301	0.83521047	2281
Oscillibacter	Cur_vs_TSP	0.036285443	0.023408505	-0.255880245	0.256606142	0.853797721	0.87656566	2266
Guopingia	Cur_vs_TSP	2.370971709	2.395711984	2.06913113	2.906094806	0.016302573	0.83521047	1993
Staphylococcus	Cur_vs_TSP	-0.183913933	-0.16684184	-0.50857148	0.280638456	0.808093247	0.83521047	1854
Limosilactobacillus	Cur_vs_TSP	-1.409525963	-1.579124039	-3.117662471	-0.674001766	0.496715935	0.83521047	1723
Catenibacillus	Cur_vs_TSP	0.542443436	0.622282566	-1.066384871	2.457833975	0.729769028	0.83521047	1660
Dehalobacterium	Cur_vs_TSP	0.466425731	0.458748953	0.124705947	0.714579259	0.398653932	0.83521047	1435
Allobaculum	Cur_vs_TSP	0.237518732	0.408009619	-4.142113928	5.172939627	0.488840621	0.83521047	1327
Angelakisella	Cur_vs_TSP	1.489894053	1.374253124	-0.206300108	2.386412399	0.51891178	0.83521047	1233
Pseudoflavonifractor	Cur_vs_TSP	0.35812027	0.35282967	-0.162261868	0.819920743	0.744729364	0.83521047	1222
Acetivibrio	Cur_vs_TSP	-0.771148754	-0.762756832	-1.096822156	-0.405470094	0.190840051	0.83521047	1218
Butyricicoccus	Cur_vs_TSP	1.64972697	1.657250429	1.312700473	1.998612255	0.060505359	0.83521047	1156
Marasmitruncus	Cur_vs_TSP	-1.228903168	-1.311987092	-2.783831173	-0.279085279	0.526172665	0.83521047	1082
Feifania	Cur_vs_TSP	0.096872424	0.10189365	-0.202708784	0.470050831	0.790467556	0.83521047	1001
Roseburia	Cur_vs_TSP	-3.952758062	-3.989496597	-5.68787309	-2.805742083	0.111171036	0.83521047	994

Peptococcus	Cur_vs_TSP	-0.419682244	-0.367245753	-0.938673217	0.343294366	0.807964681	0.83521047	986
Vampirovibrio	Cur_vs_TSP	0.792051171	0.776337901	0.445262035	1.075596756	0.307215612	0.83521047	916
Luoshenia	Cur_vs_TSP	0.553006186	0.577041205	0.25433483	1.013409821	0.45565786	0.83521047	912
Muricomes	Cur_vs_TSP	0.774354057	0.742465812	-1.166978404	2.260103235	0.656798921	0.83521047	910
Aestuariispira	Cur_vs_TSP	0.540449513	0.52631616	-1.470447852	2.388804667	0.792155689	0.83521047	906
Qiania	Cur_vs_TSP	-0.572674434	-0.584362291	-1.034688407	-0.184258944	0.44146387	0.83521047	903
Sporofaciens	Cur_vs_TSP	-1.660004799	-1.646973074	-3.854515059	0.148246059	0.558627527	0.83521047	872
Fusimonas	Cur_vs_TSP	0.573074798	0.611191842	-1.072054716	2.063081944	0.729322248	0.83521047	856
Bilophila	Cur_vs_TSP	-3.06978008	-3.174487087	-5.357883034	-1.856415912	0.221971068	0.83521047	835
Petroclostridium	Cur_vs_TSP	0.787381484	0.887751822	0.019274508	2.296412583	0.692435468	0.83521047	828
Harryflintia	Cur_vs_TSP	-0.399869476	-0.434769573	-1.139592212	0.155681879	0.70877522	0.83521047	816
Gracilibacter	Cur_vs_TSP	2.391539735	2.384775035	1.889832353	2.735419441	0.026348887	0.83521047	810
Petrocella	Cur_vs_TSP	-0.207159537	-0.344729072	-2.575815758	0.992899077	0.796291593	0.83521047	805
Leptogranulimonas	Cur_vs_TSP	-2.200948342	-2.26498243	-5.123208718	0.112226506	0.41503513	0.83521047	709
Coprococcus	Cur_vs_TSP	-0.876611297	-0.892000438	-1.309881326	-0.449167815	0.4132509	0.83521047	683
Anaerotruncus	Cur_vs_TSP	0.601522214	0.610282348	-0.360390179	1.865726591	0.736590562	0.83521047	643
Faecalimonas	Cur_vs_TSP	1.055658561	1.05803208	0.642290192	1.480558264	0.199439985	0.83521047	639
Congzhengia	Cur_vs_TSP	0.321035874	0.295090357	-0.156325501	0.688601985	0.778773306	0.83521047	630
Eubacterium	Cur_vs_TSP	-1.023426275	-1.046237649	-1.561659081	-0.471251985	0.349425005	0.83521047	587
Marvinbryantia	Cur_vs_TSP	-0.187830537	-0.211086018	-2.318738819	1.799650524	0.767768711	0.83521047	574
Odoribacter	Cur_vs_TSP	2.006813998	2.121960927	0.569835768	4.217184177	0.39368583	0.83521047	566
Enterococcus	Cur_vs_TSP	0.11778064	0.307626232	-2.764047493	3.46643314	0.615505226	0.83521047	547
Rhodospirillum	Cur_vs_TSP	3.450713544	3.668064473	1.385454656	6.907976607	0.246837993	0.83521047	544
Adlercreutzia	Cur_vs_TSP	1.398551801	1.512546977	0.631903177	2.864615788	0.391715166	0.83521047	524
Anaerocolumna	Cur_vs_TSP	-2.140708286	-2.212181956	-4.650300592	-0.013543844	0.463214641	0.83521047	523
Ruthenibacterium	Cur_vs_TSP	0.332848953	0.33133786	-0.253787297	0.854032141	0.722865485	0.83521047	523
Streptococcus	Cur_vs_TSP	-0.895563574	-0.983088954	-2.745157845	0.085133023	0.579852604	0.83521047	511
Dorea	Cur_vs_TSP	-0.588837197	-0.611245843	-1.074650679	-0.197800361	0.405845438	0.83521047	496
Faecalicatena	Cur_vs_TSP	-0.151956233	-0.183553524	-2.15997116	1.839804432	0.763354777	0.83521047	479

Saccharofermentans	Cur_vs_TSP	-1.020866373	-0.867083112	-3.641479319	2.670912938	0.667800398	0.83521047	462
Waltera	Cur_vs_TSP	-0.859792361	-0.907610807	-2.841975165	0.449971886	0.678684206	0.83521047	441
Thomasclavelia	Cur_vs_TSP	-1.996676912	-2.16902746	-3.556972365	-1.209365096	0.248453493	0.83521047	425
Jeotgalicoccus	Cur_vs_TSP	0.198903757	0.209056401	-2.608164299	3.271549175	0.659494849	0.83521047	421
Eisenbergiella	Cur_vs_TSP	-3.420212969	-3.524572038	-5.60128041	-1.839420468	0.156753611	0.83521047	418
Merdimmobilis	Cur_vs_TSP	0.217714333	0.260126374	-1.527349521	1.871521047	0.796244989	0.83521047	345
Anaeroplasma	Cur_vs_TSP	1.540664844	1.600917942	-1.092735222	4.433336496	0.564767207	0.83521047	327
Variimorphobacter	Cur_vs_TSP	0.54089768	0.665181439	-1.119801275	2.932601826	0.7299279	0.83521047	325
Natranaerovirga	Cur_vs_TSP	0.166829145	0.081392556	-1.821139302	1.82763767	0.779832644	0.83521047	313
Amedibacillus	Cur_vs_TSP	1.879537649	1.874023112	0.077717148	3.668063917	0.352967392	0.83521047	298
Fumia	Cur_vs_TSP	-0.257645931	-0.397328162	-1.996619144	0.760557266	0.745954613	0.83521047	297
Lentihominibacter	Cur_vs_TSP	0.282444392	0.283000597	-0.20217467	0.686696813	0.576657182	0.83521047	281
Corynebacterium	Cur_vs_TSP	1.47703192	1.284177582	-1.628456654	4.207318524	0.601092035	0.83521047	269
Dysosmobacter	Cur_vs_TSP	1.118655107	1.297630546	0.135768932	2.865377679	0.425529482	0.83521047	267
Anaerobium	Cur_vs_TSP	-0.298923451	-0.410732843	-3.019402786	1.199889873	0.714974662	0.83521047	255
Lactobacillus	Cur_vs_TSP	1.28872968	1.23909257	-1.479046047	3.972662295	0.631846121	0.83521047	247
Sinanaerobacter	Cur_vs_TSP	0.725605544	0.783366009	-0.450155919	2.464646523	0.646900562	0.83521047	235
Sporobacter	Cur_vs_TSP	0.745388163	0.828553676	-0.253564045	2.543214349	0.603747449	0.83521047	229
Ileibacterium	Cur_vs_TSP	0.011704769	0.213571468	-2.71196228	2.970588556	0.50502032	0.83521047	228
Ihubacter	Cur_vs_TSP	-0.95125491	-1.02864334	-2.464543173	-0.091904328	0.540239417	0.83521047	192
Enterocloster	Cur_vs_TSP	-2.181395881	-2.196400142	-5.149877911	0.355062432	0.371043807	0.83521047	186
Abyssivirga	Cur_vs_TSP	-1.271505784	-1.374067121	-2.711149614	-0.352543719	0.405999068	0.83521047	176
Acutalibacter	Cur_vs_TSP	1.549759627	1.607698061	-1.071836238	4.634222066	0.586271543	0.83521047	176
Xiamenia	Cur_vs_TSP	1.000261166	1.051272336	-0.155311321	2.512957393	0.512524778	0.83521047	174
Holdemania	Cur_vs_TSP	-0.026660471	-0.153050961	-1.703555043	0.841315286	0.734706162	0.83521047	148
Zhenpiania	Cur_vs_TSP	0.308537335	0.335973001	-0.805944248	1.574145147	0.717298099	0.83521047	142
Paludihabitans	Cur_vs_TSP	0.18499164	0.136306027	-3.479041206	3.48442384	0.619525437	0.83521047	127
Jingyaoa	Cur_vs_TSP	-2.155204568	-2.023998187	-5.725594077	0.477700138	0.450531462	0.83521047	124
Evtepia	Cur_vs_TSP	-0.490810159	-0.512802494	-3.715011237	2.419435283	0.569377419	0.83521047	119

Tannockella	Cur_vs_TSP	-0.067241642	-0.267208725	-3.234308851	2.377454135	0.613583316	0.83521047	116
Mageeibacillus	Cur_vs_TSP	-0.188730103	-0.254587584	-3.031100521	2.187288961	0.676589393	0.83521047	102
Berryella	Cur_vs_TSP	-0.4450175	-0.457975827	-2.884237844	0.9578639	0.672051138	0.83521047	95
Oxalobacter	Cur_vs_TSP	0.800164561	0.98117151	-0.371392505	3.182706161	0.514952683	0.83521047	94
Rhizobium	Cur_vs_TSP	3.176477896	3.287364879	0.266931884	6.573753087	0.227715653	0.83521047	92
Anaerobacterium	Cur_vs_TSP	2.865213326	2.901773592	-0.150114062	6.051088409	0.324035415	0.83521047	89
Breznakia	Cur_vs_TSP	2.078805017	2.161932063	-0.244360833	5.223499857	0.43147136	0.83521047	85
Raoultibacter	Cur_vs_TSP	-0.506327967	-0.499854587	-1.504250454	0.387242596	0.407162434	0.83521047	84
Frisingicoccus	Cur_vs_TSP	-1.387725732	-1.200034933	-3.950164152	2.175038609	0.515917676	0.83521047	80
Gordonibacter	Cur_vs_TSP	2.532198409	2.717955195	1.310950736	4.54268641	0.102576802	0.83521047	76
Lachnotalea	Cur_vs_TSP	0.84273993	1.006812567	-2.346058808	4.044687014	0.603226124	0.83521047	67
Hungatella	Cur_vs_TSP	-1.490339328	-1.527797982	-4.860638399	1.434971916	0.49903793	0.83521047	56
Ructibacterium	Cur_vs_TSP	-3.56164448	-3.83221814	-8.038179044	-0.743022841	0.21777484	0.83521047	55
Diplocloster	Cur_vs_TSP	-2.672892991	-2.627321233	-6.803879949	0.773152536	0.343630224	0.83521047	40
Intestinibacillus	Cur_vs_TSP	-0.085828097	-0.066869454	-3.648234477	3.696103596	0.587325857	0.83521047	30
Alkalibaculum	Cur_vs_TSP	0.742794016	0.78286189	-2.526516136	3.869669656	0.531633974	0.83521047	25
Longibaculum	Cur_vs_TSP	1.290527519	1.450277258	-1.565912252	4.809743609	0.502393519	0.83521047	25
Monoglobus	Cur_vs_TSP	-0.640174852	-0.486998772	-3.583147413	3.309432083	0.579781525	0.83521047	24
Cuneatibacter	Cur_vs_TSP	-0.88423776	-0.990077079	-4.768012409	2.293539219	0.504090408	0.83521047	21
Solibaculum	Cur_vs_TSP	-0.902699036	-0.98819464	-3.778449937	2.396865282	0.56823951	0.83521047	21
Cupriavidus	Cur_vs_TSP	1.067194037	0.893194519	-2.733940006	4.333501089	0.460183375	0.83521047	20
Gehongia	Cur_vs_TSP	0.265217225	0.161475759	-3.791581309	3.353747524	0.566936739	0.83521047	18
Jutongia	Cur_vs_TSP	-1.096628552	-1.022666857	-4.644543967	2.513638316	0.517534713	0.83521047	17
Bittarella	Cur_vs_TSP	-0.37444205	-0.235247795	-3.707639361	3.97582982	0.554819127	0.83521047	16
Massiliimalia	Cur_vs_TSP	0.022475932	0.050644827	-3.850954684	5.12605923	0.518026258	0.83521047	16
Enteroscipio	Cur_vs_TSP	-0.145154225	-6.25645E-05	-3.373321493	3.862851267	0.510174719	0.83521047	15
Hydrogeniiclostridium	Cur_vs_TSP	-1.028619734	-0.960524903	-3.719958898	1.891078483	0.523173662	0.83521047	12
Arabiibacter	Cur_vs_TSP	-0.155325218	0.063572346	-3.448486887	4.173553363	0.502381179	0.83521047	11
Aminipila	Cur_vs_TSP	0.22173761	0.161612499	-3.323949246	4.209673006	0.502823249	0.83521047	9

Victivallis	Cur_vs_TSP	-0.167049305	0.034494667	-3.553691902	3.245121107	0.607107727	0.83521047	9
Massilioclostridium	Cur_vs_TSP	1.552431914	1.734673082	-1.721222884	5.605277263	0.405996391	0.83521047	8
Pseudomonas	Cur_vs_TSP	-0.525209675	-0.580866325	-4.387224852	3.446896996	0.516171395	0.83521047	7
Scatolibacter	Cur_vs_TSP	-1.396180816	-1.458724989	-5.021081146	1.691689737	0.422956367	0.83521047	5
Blautia	Cur_vs_TSP	0.259499625	0.350160126	-2.892678995	3.845369519	0.515784009	0.83521047	4
Robinsoniella	Cur_vs_TSP	1.691909878	1.789935296	-1.453558513	5.471240997	0.424279851	0.83521047	4
Vagococcus	Cur_vs_TSP	0.997658035	1.178930397	-2.03387263	4.707649236	0.483240865	0.83521047	4
Pusillibacter	Cur_vs_TSP	-0.576430193	-0.576442172	-3.877249477	3.287714749	0.474928797	0.83521047	3
Shigella	Cur_vs_TSP	0.19109972	-0.033561066	-4.410444747	4.180984062	0.471823585	0.83521047	3
Turicibacter	Stv_vs_TSP	-0.06482747	-0.066896053	-0.219886772	0.085597111	0.877997421	0.907460422	72642
Kineothrix	Stv_vs_TSP	0.137509622	0.134498755	-0.022216447	0.291343677	0.797104825	0.852581244	70740
Duncaniella	Stv_vs_TSP	0.358382408	0.365423955	0.20983027	0.516533842	0.622467639	0.834684094	53394
Bacteroides	Stv_vs_TSP	-0.742888647	-0.742788585	-0.899724314	-0.594710915	0.197715955	0.834684094	47788
Phocaeicola	Stv_vs_TSP	-1.50098312	-1.504249461	-1.68895015	-1.338834795	0.328755356	0.834684094	45692
Alistipes	Stv_vs_TSP	0.041888971	0.042295097	-0.118089662	0.201248242	0.938911584	0.938911584	39461
Escherichia	Stv_vs_TSP	-0.462149277	-0.450491659	-0.729035694	-0.148333194	0.77127935	0.839750629	34306
Faecalibaculum	Stv_vs_TSP	-1.501623635	-1.497688031	-1.723556064	-1.277549452	0.352799412	0.834684094	24274
Parabacteroides	Stv_vs_TSP	0.301285316	0.292232025	0.11492743	0.447826145	0.687239373	0.834684094	16673
Lawsonibacter	Stv_vs_TSP	0.817168251	0.817087825	0.648337117	0.983844503	0.179441813	0.834684094	16576
Ligilactobacillus	Stv_vs_TSP	0.758505795	0.767787487	0.585303552	0.949550726	0.389577381	0.834684094	16313
Muribaculum	Stv_vs_TSP	1.070906605	1.069424765	0.904787732	1.214426161	0.20614759	0.834684094	13905
Akkermansia	Stv_vs_TSP	-3.765169557	-3.765035988	-4.025442992	-3.447583085	0.07828899	0.834684094	11460
Heminiphilus	Stv_vs_TSP	-0.694412288	-0.69505747	-0.985303344	-0.381778466	0.689472357	0.834684094	11253
Paraprevotella	Stv_vs_TSP	-3.450574511	-3.448335097	-3.722958562	-3.192406303	0.024548277	0.834684094	10096
Lachnoclostridium	Stv_vs_TSP	-1.652009828	-1.650346581	-1.894888873	-1.433743067	0.121634007	0.834684094	9496
Unclassified	Stv_vs_TSP	0.796488165	0.799712475	0.654219565	0.993906336	0.195201524	0.834684094	9340
Romboutsia	Stv_vs_TSP	7.807445518	7.981127889	6.361534091	10.64678074	0.023699058	0.834684094	9090
Simiaoa	Stv_vs_TSP	-1.206687797	-1.224816992	-1.576426106	-0.932661846	0.437278649	0.834684094	8275
Marseillibacter	Stv_vs_TSP	0.059369083	0.057612413	-0.148086612	0.262091588	0.905908688	0.91182966	7520

Paramuribaculum	Stv_vs_TSP	-1.364878293	-1.374104366	-1.609728723	-1.127389607	0.286513309	0.834684094	7407
Lacrimispora	Stv_vs_TSP	0.892007058	0.887268183	0.711244806	1.056285352	0.21638163	0.834684094	7368
Mediterraneibacter	Stv_vs_TSP	-0.674265499	-0.669662521	-0.855864328	-0.452960814	0.156275812	0.834684094	6960
Parasutterella	Stv_vs_TSP	-0.119138915	-0.122920734	-0.30907294	0.072295788	0.799889878	0.852581244	6731
Butyricimonas	Stv_vs_TSP	-0.354288338	-0.35492278	-0.54131989	-0.181135588	0.512098644	0.834684094	6433
Acetatifactor	Stv_vs_TSP	-0.148746661	-0.15221281	-0.419373646	0.070034186	0.849684776	0.890145956	5455
Vallitalea	Stv_vs_TSP	0.630486843	0.626138425	0.399109673	0.863698958	0.492432866	0.834684094	5406
Mucispirillum	Stv_vs_TSP	0.153502824	0.144992027	-0.148840314	0.446895419	0.894332965	0.91182966	5329
Clostridium	Stv_vs_TSP	0.352539335	0.350642616	0.140324205	0.554450746	0.642810957	0.834684094	5301
Bifidobacterium	Stv_vs_TSP	-2.308304065	-2.309263424	-2.77379334	-1.726617661	0.238679851	0.834684094	5234
Vescimonas	Stv_vs_TSP	-0.033314024	-0.027970732	-0.232888079	0.206296626	0.902903242	0.91182966	5154
Intestinimonas	Stv_vs_TSP	0.184296337	0.174646121	-0.098858159	0.397780975	0.749211527	0.836076632	4998
Desulfovibrio	Stv_vs_TSP	0.051357253	0.054823449	-0.176233722	0.30479366	0.895553192	0.91182966	4468
Flintibacter	Stv_vs_TSP	-0.221211059	-0.234204925	-0.444925298	-0.044405785	0.70018197	0.834684094	4382
Ruminiclostridium	Stv_vs_TSP	0.240248638	0.229980783	-0.014771985	0.449823124	0.772495338	0.839750629	4283
Ruminococcus	Stv_vs_TSP	0.624380877	0.622756758	0.405930407	0.847800696	0.625764584	0.834684094	4256
Mammaliicoccus	Stv_vs_TSP	0.324156642	0.306323632	-0.065596801	0.739391143	0.872447115	0.907460422	3810
Anaerotignum	Stv_vs_TSP	-0.308307833	-0.320615613	-0.622457294	-0.071487104	0.72648657	0.834684094	3414
Sangeribacter	Stv_vs_TSP	0.683799406	0.675342215	0.426713518	0.924627898	0.205865517	0.834684094	3235
Neglectibacter	Stv_vs_TSP	-0.533530742	-0.52355447	-0.829782449	-0.217239619	0.608282719	0.834684094	2840
Butyrivibrio	Stv_vs_TSP	-1.200718028	-1.296714312	-3.708610992	0.89827482	0.724789201	0.834684094	2593
Anaeromicropila	Stv_vs_TSP	0.565321474	0.566947435	0.284403383	0.858458289	0.528411028	0.834684094	2464
Christensenella	Stv_vs_TSP	-1.007935843	-1.00246007	-1.282940526	-0.730872315	0.299447511	0.834684094	2461
Barnesiella	Stv_vs_TSP	3.166318288	3.38305326	0.766846614	6.155900786	0.400464965	0.834684094	2281
Oscillibacter	Stv_vs_TSP	-0.675676635	-0.689637436	-0.986736549	-0.386378936	0.512460702	0.834684094	2266
Guopingia	Stv_vs_TSP	1.319592115	1.334082676	0.936865665	1.713703967	0.215430062	0.834684094	1993
Staphylococcus	Stv_vs_TSP	-0.882636174	-0.924371202	-1.626673314	-0.320944469	0.636851232	0.834684094	1854
Limosilactobacillus	Stv_vs_TSP	-3.829387565	-3.906135234	-5.692723276	-2.50059469	0.18649357	0.834684094	1723
Catenibacillus	Stv_vs_TSP	1.971398097	2.092983515	1.155961956	3.68179352	0.277672676	0.834684094	1660

Dehalobacterium	Stv_vs_TSP	0.760995179	0.75545667	0.524218592	0.977964607	0.139641996	0.834684094	1435
Allobaculum	Stv_vs_TSP	0.169622075	0.396423066	-3.476487284	4.744244868	0.507386602	0.834684094	1327
Angelakisella	Stv_vs_TSP	-1.78130422	-2.001380102	-4.571744257	-0.421325283	0.440014398	0.834684094	1233
Pseudoflavonifractor	Stv_vs_TSP	0.485318414	0.485479672	0.125093546	0.901309853	0.551230994	0.834684094	1222
Acetivibrio	Stv_vs_TSP	-0.611613357	-0.604582494	-0.978827454	-0.286691096	0.571029769	0.834684094	1218
Butyricicoccus	Stv_vs_TSP	0.841635182	0.84991254	0.466424326	1.223797324	0.237158574	0.834684094	1156
Marasmitruncus	Stv_vs_TSP	0.627190886	0.629645348	0.148544424	1.171875309	0.66092951	0.834684094	1082
Feifania	Stv_vs_TSP	-0.169324904	-0.166762942	-0.542236386	0.226493752	0.802755068	0.852581244	1001
Roseburia	Stv_vs_TSP	-3.359837535	-3.56144628	-5.518612876	-2.361895376	0.139695116	0.834684094	994
Peptococcus	Stv_vs_TSP	-0.602011393	-0.601471528	-1.211085211	0.223248214	0.670263972	0.834684094	986
Vampirovibrio	Stv_vs_TSP	0.07670756	0.077253402	-0.293231488	0.442203058	0.817913727	0.862730918	916
Luoshenia	Stv_vs_TSP	-0.762791398	-0.895530402	-2.369333839	0.072121005	0.646850347	0.834684094	912
Muricomes	Stv_vs_TSP	3.137764524	3.22414815	2.367802087	4.663826582	0.072631183	0.834684094	910
Aestuariispira	Stv_vs_TSP	-2.895988873	-2.97248064	-6.005893079	-0.780497405	0.435124343	0.834684094	906
Qiania	Stv_vs_TSP	-1.255859402	-1.302289786	-2.69772372	-0.276342741	0.528408863	0.834684094	903
Sporofaciens	Stv_vs_TSP	0.649253038	0.672971766	-1.147225302	2.821943446	0.727419868	0.834684094	872
Fusimonas	Stv_vs_TSP	2.604620166	2.70686644	1.786600031	4.415999583	0.184131948	0.834684094	856
Bilophila	Stv_vs_TSP	0.643484136	0.664703321	0.294573545	1.116685621	0.589877208	0.834684094	835
Petroclostridium	Stv_vs_TSP	1.065481112	1.167235993	0.291842013	2.59182684	0.597776488	0.834684094	828
Harryflintia	Stv_vs_TSP	-0.586453684	-0.662222859	-1.988834835	0.338064662	0.715445114	0.834684094	816
Gracilibacter	Stv_vs_TSP	0.519989898	0.371417234	-1.118685198	1.378131064	0.717173815	0.834684094	810
Petrocella	Stv_vs_TSP	-2.357735666	-2.423697565	-4.504950553	-0.802898367	0.39074553	0.834684094	805
Leptogranulimonas	Stv_vs_TSP	-5.451797433	-5.413929924	-7.75894552	-2.794523923	0.097818907	0.834684094	709
Coprococcus	Stv_vs_TSP	-0.708568249	-0.716508838	-1.074363014	-0.311109126	0.37918742	0.834684094	683
Anaerotruncus	Stv_vs_TSP	2.479264188	2.561804644	1.596842268	3.663455423	0.150358837	0.834684094	643
Faecalimonas	Stv_vs_TSP	0.386252007	0.396590869	-0.032491077	0.882703139	0.626130546	0.834684094	639
Congzhengia	Stv_vs_TSP	-0.937159951	-0.953959791	-1.453721956	-0.49557435	0.457810547	0.834684094	630
Eubacterium	Stv_vs_TSP	-1.724195607	-1.831869454	-3.406055194	-0.862919295	0.407809221	0.834684094	587
Marvinbryantia	Stv_vs_TSP	0.582480562	0.589872024	-1.397799896	2.514145421	0.723994487	0.834684094	574

Odoribacter	Stv_vs_TSP	1.227176178	1.189566759	-1.273449481	3.441042693	0.657155503	0.834684094	566
Enterococcus	Stv_vs_TSP	1.837471616	1.915807811	-1.25208946	5.463376582	0.458473851	0.834684094	547
Rhodospirillum	Stv_vs_TSP	4.0266297	4.065655046	1.526819936	7.614738581	0.201957634	0.834684094	544
Adlercreutzia	Stv_vs_TSP	0.78883516	0.868074823	-0.076639728	2.294073333	0.654261361	0.834684094	524
Anaerocolumna	Stv_vs_TSP	2.296255398	2.455243055	1.190221747	4.115045187	0.318211853	0.834684094	523
Ruthenibacterium	Stv_vs_TSP	-1.160370705	-1.235784264	-2.839549779	-0.202352198	0.557623616	0.834684094	523
Streptococcus	Stv_vs_TSP	1.607076162	1.6064286	1.110223567	2.139405962	0.194086409	0.834684094	511
Dorea	Stv_vs_TSP	-1.345438859	-1.419034127	-2.716677731	-0.531448136	0.445613436	0.834684094	496
Faecalicatena	Stv_vs_TSP	-0.720432351	-0.721378515	-3.072382388	1.962075541	0.691481112	0.834684094	479
Saccharofermentans	Stv_vs_TSP	-0.188825244	-0.179994046	-2.356062537	2.624983583	0.744130131	0.836076632	462
Waltera	Stv_vs_TSP	-0.043681148	-0.205286641	-1.834145891	0.863883592	0.774315515	0.839750629	441
Thomasclavelia	Stv_vs_TSP	-2.873614313	-2.979039781	-4.602227302	-1.965301191	0.075242266	0.834684094	425
Jeotgalicoccus	Stv_vs_TSP	0.503128883	0.573315688	-1.97367375	3.106431759	0.730631531	0.834684094	421
Eisenbergiella	Stv_vs_TSP	-1.02300687	-1.17388407	-2.615598024	-0.257201108	0.504099042	0.834684094	418
Merdimmobilis	Stv_vs_TSP	-1.26653835	-1.060068972	-2.704496798	1.034236576	0.618931898	0.834684094	345
Anaeroplasma	Stv_vs_TSP	0.847439566	0.886666972	-1.789726764	3.926828228	0.669471045	0.834684094	327
Variimorphobacter	Stv_vs_TSP	-2.897022221	-3.125379905	-6.571249651	-0.353455272	0.359443108	0.834684094	325
Natranaerovirga	Stv_vs_TSP	-0.224223701	-0.165816314	-1.728469126	1.402029641	0.76605295	0.839750629	313
Amedibacillus	Stv_vs_TSP	2.321594277	2.413052167	1.020896171	4.022299079	0.165001768	0.834684094	298
Fumia	Stv_vs_TSP	-0.261343433	-0.397727848	-2.080682115	0.791412481	0.71610062	0.834684094	297
Lentihominibacter	Stv_vs_TSP	-0.618122991	-0.609570454	-1.232544262	0.030805538	0.487720028	0.834684094	281
Corynebacterium	Stv_vs_TSP	2.963664601	3.047636185	1.37703656	5.638379896	0.215002368	0.834684094	269
Dysosmobacter	Stv_vs_TSP	-1.756770993	-1.807721832	-4.330968288	0.41056765	0.479117926	0.834684094	267
Anaerobium	Stv_vs_TSP	0.169878729	0.198668227	-1.616477918	1.872262795	0.7108955	0.834684094	255
Lactobacillus	Stv_vs_TSP	-0.628402615	-0.55200344	-3.423329543	2.825313531	0.661102273	0.834684094	247
Sinanaerobacter	Stv_vs_TSP	0.124525664	0.229556512	-1.367983519	2.287250544	0.731703589	0.834684094	235
Sporobacter	Stv_vs_TSP	1.405990211	1.49601951	0.334530401	3.099610792	0.356685436	0.834684094	229
Ileibacterium	Stv_vs_TSP	-0.040647771	-0.062096927	-3.613279164	3.762398061	0.529982221	0.834684094	228
Ihubacter	Stv_vs_TSP	-0.179826385	-0.16596289	-0.900609712	0.616769412	0.746266523	0.836076632	192

Enterocloster	Stv_vs_TSP	1.722831752	1.789482477	0.627812825	3.320281519	0.247396632	0.834684094	186
Abyssivirga	Stv_vs_TSP	-2.169553881	-2.366920453	-4.086147918	-1.060160294	0.216970292	0.834684094	176
Acutalibacter	Stv_vs_TSP	-1.53004407	-1.370621463	-4.611491853	2.098034269	0.533323542	0.834684094	176
Xiamenia	Stv_vs_TSP	-0.750306727	-0.784908176	-2.682481234	1.196114225	0.687864477	0.834684094	174
Holdemania	Stv_vs_TSP	0.197110001	0.20902869	-0.434133259	0.9332117	0.714758846	0.834684094	148
Zhenpiania	Stv_vs_TSP	1.080521351	1.129911532	0.082239051	2.496518577	0.470666024	0.834684094	142
Paludihabitans	Stv_vs_TSP	2.156641295	2.1774479	-0.855284856	5.099413308	0.399658656	0.834684094	127
Jingyaoa	Stv_vs_TSP	-0.829996462	-0.83504527	-4.354955865	2.223810186	0.61422082	0.834684094	124
Evtepia	Stv_vs_TSP	1.794753558	1.711734941	-1.765043171	5.082532108	0.550454595	0.834684094	119
Tannockella	Stv_vs_TSP	3.267629419	3.332232313	1.188548356	5.972934542	0.201778454	0.834684094	116
Mageeibacillus	Stv_vs_TSP	0.075745765	0.114699915	-2.615984869	2.31242188	0.709187948	0.834684094	102
Berryella	Stv_vs_TSP	1.100543059	1.132375689	0.254260874	2.375644507	0.405649193	0.834684094	95
Oxalobacter	Stv_vs_TSP	-0.021074178	0.068049477	-2.186556799	2.589337009	0.670875423	0.834684094	94
Rhizobium	Stv_vs_TSP	4.788825015	4.935036727	2.359521992	8.633134627	0.071320221	0.834684094	92
Anaerobacterium	Stv_vs_TSP	-0.499104586	-0.503128925	-3.707211671	3.271357453	0.591365522	0.834684094	89
Breznakia	Stv_vs_TSP	-1.22115958	-1.2948702	-4.572731518	1.469238215	0.525433871	0.834684094	85
Raoultibacter	Stv_vs_TSP	-1.550458161	-1.654873641	-3.086398299	-0.547224509	0.187369872	0.834684094	84
Frisingicoccus	Stv_vs_TSP	2.056973489	2.005553137	-0.890266846	5.10640966	0.457371854	0.834684094	80
Gordonibacter	Stv_vs_TSP	-1.948695782	-2.058634878	-5.425281459	0.829456581	0.398866029	0.834684094	76
Lachnotalea	Stv_vs_TSP	1.359644264	1.340732174	-1.031964332	4.347871655	0.535707668	0.834684094	67
Hungatella	Stv_vs_TSP	-1.243048052	-1.243511349	-3.992520282	2.265929794	0.555965678	0.834684094	56
Ructibacterium	Stv_vs_TSP	-4.088347419	-4.118102637	-7.303842517	-1.262331186	0.179087924	0.834684094	55
Diplocloster	Stv_vs_TSP	-1.296003306	-1.146939494	-4.761956717	2.219755412	0.567512585	0.834684094	40
Intestinibacillus	Stv_vs_TSP	-0.948049479	-0.982934041	-4.287886489	2.563277672	0.47079829	0.834684094	30
Alkalibaculum	Stv_vs_TSP	1.116024342	1.057604837	-2.427691215	4.210852051	0.523963297	0.834684094	25
Longibaculum	Stv_vs_TSP	0.581894967	0.403638434	-3.416986787	3.987981354	0.554129748	0.834684094	25
Monoglobus	Stv_vs_TSP	-0.040212204	-0.007520126	-3.232446088	3.417664976	0.588482461	0.834684094	24
Cuneatibacter	Stv_vs_TSP	1.542879901	1.523342243	-1.554346637	4.8679629	0.491061318	0.834684094	21
Solibaculum	Stv_vs_TSP	-1.948318947	-1.974036456	-5.439054338	1.926090143	0.399071975	0.834684094	21

Cupriavidus	Stv_vs_TSP	1.851303733	1.965704793	-1.496750236	5.118115483	0.426084987	0.834684094	20
Gehongia	Stv_vs_TSP	0.502720578	0.41644822	-2.860741278	3.504724171	0.573273313	0.834684094	18
Jutongia	Stv_vs_TSP	0.114195944	0.362758732	-3.368138272	4.883653652	0.601725665	0.834684094	17
Bittarella	Stv_vs_TSP	-1.01320414	-1.172232153	-5.012004609	2.629033601	0.509942994	0.834684094	16
Massiliimalia	Stv_vs_TSP	1.563674135	1.273456369	-2.572300788	4.631937637	0.443108569	0.834684094	16
Enteroscipio	Stv_vs_TSP	1.289286988	1.30475676	-3.539771361	4.986905341	0.462281765	0.834684094	15
Hydrogeniiclostridium	Stv_vs_TSP	-1.839848343	-2.039644995	-5.459799997	0.914908983	0.394509681	0.834684094	12
Arabiibacter	Stv_vs_TSP	1.983083725	1.860382512	-1.37891294	5.622783349	0.408124148	0.834684094	11
Aminipila	Stv_vs_TSP	1.297201663	1.187912893	-2.820202224	5.460693063	0.477008946	0.834684094	9
Victivallis	Stv_vs_TSP	-1.102825377	-1.25677401	-5.92911187	2.247331688	0.462106393	0.834684094	9
Massilioclostridium	Stv_vs_TSP	0.959653043	0.954494441	-2.29914142	4.946366016	0.502711896	0.834684094	8
Pseudomonas	Stv_vs_TSP	0.011333095	0.124667783	-3.12304729	3.860785383	0.595457061	0.834684094	7
Scatolibacter	Stv_vs_TSP	-1.722251382	-1.580392923	-4.612894706	1.884165098	0.416535403	0.834684094	5
Blautia	Stv_vs_TSP	0.92594341	0.856153936	-3.078330061	3.658979883	0.480769905	0.834684094	4
Robinsoniella	Stv_vs_TSP	0.150451165	0.097193769	-3.990525753	3.858347783	0.478951615	0.834684094	4
Vagococcus	Stv_vs_TSP	0.643830894	0.854498927	-2.551001124	4.510892872	0.479194194	0.834684094	4
Pusillibacter	Stv_vs_TSP	0.077420071	-0.006750095	-4.082060203	3.629882077	0.509450796	0.834684094	3
Shigella	Stv_vs_TSP	0.638436869	0.859224569	-2.847246618	4.577170825	0.484780926	0.834684094	3
Turicibacter	Stv_vs_Cur	0.377432724	0.386092906	0.227231115	0.59373116	0.512630298	0.857616425	72642
Kineothrix	Stv_vs_Cur	-0.163937081	-0.159853614	-0.305371329	0.050240187	0.814770649	0.857616425	70740
Duncaniella	Stv_vs_Cur	-0.089621835	-0.077510091	-0.22007013	0.134598706	0.891163576	0.908868812	53394
Bacteroides	Stv_vs_Cur	-0.708648615	-0.692927807	-0.845780198	-0.504827177	0.347415416	0.857616425	47788
Phocaeicola	Stv_vs_Cur	-1.017984068	-1.016193418	-1.210794146	-0.794220972	0.554035622	0.857616425	45692
Alistipes	Stv_vs_Cur	-1.619529279	-1.611952554	-1.771642728	-1.409748436	0.127388903	0.857616425	39461
Escherichia	Stv_vs_Cur	3.390345123	3.400239387	2.945180381	3.812791614	0.094676551	0.857616425	34306
Faecalibaculum	Stv_vs_Cur	-2.292037197	-2.292348841	-2.513527953	-2.092177168	0.103644385	0.857616425	24274
Parabacteroides	Stv_vs_Cur	0.610960974	0.610849491	0.461934549	0.820419971	0.484352823	0.857616425	16673
Lawsonibacter	Stv_vs_Cur	-0.161205883	-0.154213836	-0.283338208	0.060795679	0.806038853	0.857616425	16576
Ligilactobacillus	Stv_vs_Cur	-1.72757432	-1.716111459	-1.892196526	-1.490248529	0.134284649	0.857616425	16313

Muribaculum	Stv_vs_Cur	0.457031029	0.464421192	0.318379881	0.663543987	0.507609003	0.857616425	13905
Akkermansia	Stv_vs_Cur	0.891276856	0.883819669	0.466695325	1.380707648	0.508002601	0.857616425	11460
Heminiphilus	Stv_vs_Cur	-0.71379643	-0.716656331	-0.999166176	-0.464213343	0.656179504	0.857616425	11253
Paraprevotella	Stv_vs_Cur	-1.494154876	-1.497510818	-1.830713393	-1.129511938	0.383336576	0.857616425	10096
Lachnoclostridium	Stv_vs_Cur	-1.12330341	-1.111264603	-1.303861896	-0.892577657	0.270833684	0.857616425	9496
Unclassified	Stv_vs_Cur	-0.102842599	-0.088826291	-0.267448973	0.12497234	0.834115982	0.857616425	9340
Romboutsia	Stv_vs_Cur	2.019046667	2.02746788	1.825700224	2.250903799	0.012405846	0.857616425	9090
Simiaoa	Stv_vs_Cur	-0.564624001	-0.597960892	-0.972600822	-0.313330047	0.744387032	0.857616425	8275
Marseillibacter	Stv_vs_Cur	-0.504459347	-0.504447767	-0.70785486	-0.287784496	0.583643476	0.857616425	7520
Paramuribaculum	Stv_vs_Cur	-0.019616435	-0.014515798	-0.308490592	0.256959648	0.931309815	0.931309815	7407
Lacrimispora	Stv_vs_Cur	1.591482591	1.604743835	1.385381737	1.807939837	0.004162507	0.641026013	7368
Mediterraneibacter	Stv_vs_Cur	-0.433434948	-0.427334007	-0.623551321	-0.220767062	0.321214873	0.857616425	6960
Parasutterella	Stv_vs_Cur	0.184365628	0.19063741	-0.004133741	0.450225748	0.824238093	0.857616425	6731
Butyricimonas	Stv_vs_Cur	0.424009962	0.416431251	0.218741434	0.650151303	0.493179976	0.857616425	6433
Acetatifactor	Stv_vs_Cur	2.224379733	2.415803216	1.578647089	3.950841888	0.292680983	0.857616425	5455
Vallitalea	Stv_vs_Cur	-0.99759924	-0.984513062	-1.173285988	-0.742449133	0.279888426	0.857616425	5406
Mucispirillum	Stv_vs_Cur	0.338191125	0.327691158	0.03184419	0.658642792	0.799932766	0.857616425	5329
Clostridium	Stv_vs_Cur	0.431080137	0.439185549	0.228785353	0.675208654	0.622896776	0.857616425	5301
Bifidobacterium	Stv_vs_Cur	2.131957614	2.31205654	0.929501682	4.692224658	0.402791271	0.857616425	5234
Vescimonas	Stv_vs_Cur	0.00039265	0.017563887	-0.182900837	0.279290693	0.910683579	0.922666258	5154
Intestinimonas	Stv_vs_Cur	-0.277348744	-0.277596312	-0.493723391	-0.070451052	0.637180044	0.857616425	4998
Desulfovibrio	Stv_vs_Cur	-0.237499445	-0.228121149	-0.449402311	-0.011624158	0.730488018	0.857616425	4468
Flintibacter	Stv_vs_Cur	-0.225043661	-0.239992051	-0.470808207	-0.018188903	0.713621709	0.857616425	4382
Ruminiclostridium	Stv_vs_Cur	-1.376319444	-1.366403423	-1.561943457	-1.180083294	0.065945631	0.857616425	4283
Ruminococcus	Stv_vs_Cur	0.107580879	0.108620293	-0.064319325	0.317606752	0.921591141	0.927614613	4256
Mammaliicoccus	Stv_vs_Cur	1.032715772	1.041851815	0.664093676	1.484205741	0.572094785	0.857616425	3810
Anaerotignum	Stv_vs_Cur	0.275922998	0.287438099	0.035074901	0.573971729	0.771489496	0.857616425	3414
Sangeribacter	Stv_vs_Cur	0.463588634	0.466914565	0.271309706	0.715142506	0.424609629	0.857616425	3235
Neglectibacter	Stv_vs_Cur	-0.229572386	-0.228292444	-0.501011216	0.084662923	0.776157625	0.857616425	2840

Butyrivibrio	Stv_vs_Cur	-1.261698393	-1.35820691	-3.908786615	0.918386436	0.718308408	0.857616425	2593
Anaeromicropila	Stv_vs_Cur	-0.593646759	-0.593784139	-0.881220695	-0.325891357	0.499951712	0.857616425	2464
Christensenella	Stv_vs_Cur	-0.202552967	-0.221854005	-0.534010075	0.113720076	0.816951087	0.857616425	2461
Barnesiella	Stv_vs_Cur	0.602182458	0.558057736	-1.233492374	2.079440187	0.79552689	0.857616425	2281
Oscillibacter	Stv_vs_Cur	-0.726999205	-0.713045942	-0.999182637	-0.423927781	0.503733271	0.857616425	2266
Guopingia	Stv_vs_Cur	-1.063942365	-1.061629308	-1.332393995	-0.807568371	0.278775039	0.857616425	1993
Staphylococcus	Stv_vs_Cur	-0.687463481	-0.757529362	-1.491126722	-0.234610592	0.674711859	0.857616425	1854
Limosilactobacillus	Stv_vs_Cur	-2.320098383	-2.327011195	-4.307408383	-0.077089687	0.496824954	0.857616425	1723
Catenibacillus	Stv_vs_Cur	1.399017614	1.470700949	0.560376568	3.007112809	0.451210757	0.857616425	1660
Dehalobacterium	Stv_vs_Cur	0.279681664	0.296707717	0.029199507	0.635379982	0.598402722	0.857616425	1435
Allobaculum	Stv_vs_Cur	0.12857206	-0.011586553	-4.486879263	3.340996134	0.529897598	0.857616425	1327
Angelakisella	Stv_vs_Cur	-3.286123895	-3.375633226	-5.911950437	-1.211695564	0.309632361	0.857616425	1233
Pseudoflavonifractor	Stv_vs_Cur	0.15722915	0.132650002	-0.301533881	0.544645535	0.835340673	0.857616425	1222
Acetivibrio	Stv_vs_Cur	0.176888829	0.158174338	-0.314426652	0.580209287	0.834007066	0.857616425	1218
Butyricicoccus	Stv_vs_Cur	-0.811660977	-0.80733789	-1.15037612	-0.527177682	0.343397996	0.857616425	1156
Marasmitruncus	Stv_vs_Cur	1.886612697	1.94163244	1.097874742	3.190750146	0.283596699	0.857616425	1082
Feifania	Stv_vs_Cur	-0.291507251	-0.268656592	-0.648178989	0.102140762	0.719539101	0.857616425	1001
Roseburia	Stv_vs_Cur	0.440839097	0.428050318	-1.590157545	2.271674688	0.76082251	0.857616425	994
Peptococcus	Stv_vs_Cur	-0.234757741	-0.234225775	-0.692634095	0.313547914	0.828721102	0.857616425	986
Vampirovibrio	Stv_vs_Cur	-0.702016303	-0.699084499	-0.974465455	-0.311147489	0.317509789	0.857616425	916
Luoshenia	Stv_vs_Cur	-1.371230788	-1.472571607	-2.820656425	-0.607845887	0.416595994	0.857616425	912
Muricomes	Stv_vs_Cur	2.332454366	2.481682338	1.373361895	4.241688624	0.212824921	0.857616425	910
Aestuariispira	Stv_vs_Cur	-3.483102261	-3.4987968	-6.174700099	-0.940922234	0.30490369	0.857616425	906
Qiania	Stv_vs_Cur	-0.675840749	-0.717927495	-1.968758236	0.360675076	0.719660054	0.857616425	903
Sporofaciens	Stv_vs_Cur	2.35226737	2.319944841	0.295368055	4.339377871	0.424584254	0.857616425	872
Fusimonas	Stv_vs_Cur	1.964042339	2.095674598	1.150454507	3.638807127	0.309492545	0.857616425	856
Bilophila	Stv_vs_Cur	3.673060901	3.839190409	2.392001008	5.891407481	0.146200429	0.857616425	835
Petroclostridium	Stv_vs_Cur	0.267939863	0.279484171	-0.066765261	0.670688313	0.767946217	0.857616425	828
Harryflintia	Stv_vs_Cur	-0.10016853	-0.227453286	-1.745256941	0.807738867	0.820761857	0.857616425	816

Gracilibacter	Stv_vs_Cur	-1.907635345	-2.013357801	-3.295537817	-1.158646043	0.332673843	0.857616425	810
Petrocella	Stv_vs_Cur	-2.053549389	-2.078968493	-4.455365722	0.584953078	0.545750715	0.857616425	805
Leptogranulimonas	Stv_vs_Cur	-3.179900215	-3.148947494	-6.001570711	-0.287281905	0.310901372	0.857616425	709
Coprococcus	Stv_vs_Cur	0.186614108	0.175491601	-0.361813096	0.64534146	0.830760203	0.857616425	683
Anaerotruncus	Stv_vs_Cur	1.972776048	1.951522296	1.414251708	2.48154011	0.089565602	0.857616425	643
Faecalimonas	Stv_vs_Cur	-0.640595119	-0.66144121	-1.115429532	-0.30725968	0.428462108	0.857616425	639
Congzhengia	Stv_vs_Cur	-1.214795397	-1.249050149	-1.772055848	-0.756320875	0.253204289	0.857616425	630
Eubacterium	Stv_vs_Cur	-0.690563175	-0.785631805	-2.542551137	0.254517642	0.738480994	0.857616425	587
Marvinbryantia	Stv_vs_Cur	0.731583494	0.800958042	-1.289099826	3.264967235	0.691293554	0.857616425	574
Odoribacter	Stv_vs_Cur	-0.928713903	-0.932394168	-2.838820747	1.276190095	0.731925818	0.857616425	566
Enterococcus	Stv_vs_Cur	1.477047153	1.608181579	-1.352411866	4.425759909	0.493589565	0.857616425	547
Rhodospirillum	Stv_vs_Cur	0.41541695	0.397590573	-1.376014715	2.133635688	0.789496002	0.857616425	544
Adlercreutzia	Stv_vs_Cur	-0.641514636	-0.644472154	-1.097829548	-0.118215765	0.352747784	0.857616425	524
Anaerocolumna	Stv_vs_Cur	4.637305847	4.667425011	3.034920626	6.593071551	0.055641858	0.857616425	523
Ruthenibacterium	Stv_vs_Cur	-1.438908443	-1.567122124	-3.202669207	-0.375936786	0.446176226	0.857616425	523
Streptococcus	Stv_vs_Cur	2.525792744	2.589517554	1.564654579	4.513710645	0.156778564	0.857616425	511
Dorea	Stv_vs_Cur	-0.710575108	-0.807788284	-2.058019901	0.08318853	0.660749658	0.857616425	496
Faecalicatena	Stv_vs_Cur	-0.556635862	-0.537824992	-3.113565199	1.97267546	0.703875306	0.857616425	479
Saccharofermentans	Stv_vs_Cur	0.804174012	0.687089066	-1.829484319	2.287802368	0.700875589	0.857616425	462
Waltera	Stv_vs_Cur	0.843362626	0.702324166	-1.498042468	2.603913138	0.716943304	0.857616425	441
Thomasclavelia	Stv_vs_Cur	-0.819272821	-0.81001232	-2.846931257	0.778788539	0.652795097	0.857616425	425
Jeotgalicoccus	Stv_vs_Cur	0.307180674	0.364259287	-2.266713611	3.352134897	0.75043461	0.857616425	421
Eisenbergiella	Stv_vs_Cur	2.381001383	2.350687968	0.194246366	4.493810974	0.388875089	0.857616425	418
Merdimmobilis	Stv_vs_Cur	-1.33577575	-1.320195346	-3.205120131	0.597498802	0.550064694	0.857616425	345
Anaeroplasma	Stv_vs_Cur	-0.766371015	-0.714250971	-2.795873321	1.637283992	0.700354551	0.857616425	327
Variimorphobacter	Stv_vs_Cur	-3.611027809	-3.790561344	-7.244457504	-1.236699247	0.207479954	0.857616425	325
Natranaerovirga	Stv_vs_Cur	-0.312886932	-0.247208871	-2.304113229	1.59748305	0.750339608	0.857616425	313
Amedibacillus	Stv_vs_Cur	0.372332276	0.539029055	-0.506943537	2.362054534	0.75532723	0.857616425	298
Fumia	Stv_vs_Cur	0.045824294	-0.000399686	-1.817988992	1.385815263	0.79134269	0.857616425	297

Lentihominibacter	Stv_vs_Cur	-0.924426867	-0.892571051	-1.382830836	-0.307156363	0.327471713	0.857616425	281
Corynebacterium	Stv_vs_Cur	1.570992599	1.763458603	0.15250285	4.207784403	0.464991537	0.857616425	269
Dysosmobacter	Stv_vs_Cur	-3.121091084	-3.105352378	-5.471999025	-1.5683637	0.167650984	0.857616425	267
Anaerobium	Stv_vs_Cur	0.608148114	0.60940107	-1.178883275	2.797255219	0.646360747	0.857616425	255
Lactobacillus	Stv_vs_Cur	-1.808095665	-1.79109601	-5.178966902	1.593185335	0.553504917	0.857616425	247
Sinanaerobacter	Stv_vs_Cur	-0.463202609	-0.553809497	-1.896741929	0.64623241	0.690226259	0.857616425	235
Sporobacter	Stv_vs_Cur	0.658622208	0.667465835	-0.058850822	1.448028343	0.517849688	0.857616425	229
Ileibacterium	Stv_vs_Cur	-0.452457553	-0.275668395	-3.614851348	3.582271042	0.487263681	0.857616425	228
Ihubacter	Stv_vs_Cur	0.793347644	0.86268045	-0.335647902	2.139327113	0.606322818	0.857616425	192
Enterocloster	Stv_vs_Cur	3.754502629	3.985882619	2.119595813	7.082809895	0.066054466	0.857616425	186
Abyssivirga	Stv_vs_Cur	-0.872822424	-0.992853332	-3.249169209	0.948733093	0.634007903	0.857616425	176
Acutalibacter	Stv_vs_Cur	-2.79170528	-2.978319524	-6.175908928	0.05073674	0.324705145	0.857616425	176
Xiamenia	Stv_vs_Cur	-1.686714877	-1.836180512	-4.185940073	-0.45113063	0.439128761	0.857616425	174
Holdemania	Stv_vs_Cur	0.206210698	0.362079651	-0.796646831	2.156035279	0.748419847	0.857616425	148
Zhenpiania	Stv_vs_Cur	0.698959391	0.793938531	0.025338679	1.663015603	0.459694219	0.857616425	142
Paludihabitans	Stv_vs_Cur	1.963170884	2.041141874	-0.931773826	4.954025183	0.422753726	0.857616425	127
Jingyaoa	Stv_vs_Cur	1.033273225	1.188952917	-2.185013316	4.313010811	0.578910166	0.857616425	124
Evtepia	Stv_vs_Cur	2.144783614	2.224537435	-0.973223859	5.625348804	0.482107007	0.857616425	119
Tannockella	Stv_vs_Cur	3.469204818	3.599441038	1.551248472	6.722532238	0.154524258	0.857616425	116
Mageeibacillus	Stv_vs_Cur	0.305741846	0.369287499	-2.115315188	2.769617915	0.683827159	0.857616425	102
Berryella	Stv_vs_Cur	1.459438185	1.590351516	0.38672982	3.142937297	0.223221963	0.857616425	95
Oxalobacter	Stv_vs_Cur	-0.826298919	-0.913122033	-2.526885884	0.356903452	0.501451704	0.857616425	94
Rhizobium	Stv_vs_Cur	1.631240966	1.647671848	-0.223771314	3.322162402	0.461893178	0.857616425	92
Anaerobacterium	Stv_vs_Cur	-3.469374771	-3.404902517	-5.862254017	-0.758411381	0.260693177	0.857616425	89
Breznakia	Stv_vs_Cur	-3.370845799	-3.456802263	-7.082913379	-0.491275373	0.246302941	0.857616425	85
Raoultibacter	Stv_vs_Cur	-1.028733065	-1.155019054	-2.674454784	-0.049167583	0.377892545	0.857616425	84
Frisingicoccus	Stv_vs_Cur	3.148859196	3.20558807	0.221241134	6.384416795	0.232761505	0.857616425	80
Gordonibacter	Stv_vs_Cur	-4.601057216	-4.776590073	-7.994968988	-2.693462643	0.036970934	0.857616425	76
Lachnotalea	Stv_vs_Cur	0.266930685	0.333919607	-1.823211222	3.266601202	0.665329515	0.857616425	67

Hungatella	Stv_vs_Cur	0.339442699	0.284286633	-3.220534339	3.658001356	0.620209807	0.857616425	56
Ructibacterium	Stv_vs_Cur	-0.219688319	-0.285884498	-4.040485986	4.274087452	0.543366468	0.857616425	55
Diplocloster	Stv_vs_Cur	1.317122586	1.480381739	-2.16349829	5.730362265	0.490505985	0.857616425	40
Intestinibacillus	Stv_vs_Cur	-0.906973051	-0.916064587	-4.284918014	2.692739861	0.501544088	0.857616425	30
Alkalibaculum	Stv_vs_Cur	0.238643926	0.274742947	-2.605180409	3.742829141	0.57828397	0.857616425	25
Longibaculum	Stv_vs_Cur	-0.954445867	-1.046638824	-4.217459947	2.126307811	0.582890074	0.857616425	25
Monoglobus	Stv_vs_Cur	0.459882585	0.479478646	-2.561334032	3.25280262	0.62085773	0.857616425	24
Cuneatibacter	Stv_vs_Cur	2.56206395	2.513419322	-0.949835456	5.923787778	0.343242972	0.857616425	21
Solibaculum	Stv_vs_Cur	-1.015612129	-0.985841816	-5.069603036	2.978604257	0.490733019	0.857616425	21
Cupriavidus	Stv_vs_Cur	0.772170938	1.072510274	-1.784406838	4.982628864	0.569696416	0.857616425	20
Gehongia	Stv_vs_Cur	0.194195069	0.254972461	-3.127585944	4.667425076	0.592314975	0.857616425	18
Jutongia	Stv_vs_Cur	1.441732691	1.385425589	-1.75125295	4.568231674	0.4444924	0.857616425	17
Bittarella	Stv_vs_Cur	-0.941862174	-0.936984358	-4.901964194	3.208932466	0.507300384	0.857616425	16
Massiliimalia	Stv_vs_Cur	1.448728033	1.222811542	-2.593399181	4.880175978	0.508559571	0.857616425	16
Enteroscipio	Stv_vs_Cur	1.257070708	1.304819325	-2.963889295	5.414333626	0.468113033	0.857616425	15
Hydrogeniiclostridium	Stv_vs_Cur	-0.815655572	-1.079120092	-4.913407769	2.09113993	0.484641759	0.857616425	12
Arabiibacter	Stv_vs_Cur	1.984933887	1.796810166	-1.995589165	4.589257347	0.412138613	0.857616425	11
Aminipila	Stv_vs_Cur	0.967128643	1.026300394	-2.160552606	5.008116547	0.480415818	0.857616425	9
Victivallis	Stv_vs_Cur	-1.516388351	-1.291268677	-5.059490136	2.211761532	0.440178633	0.857616425	9
Massilioclostridium	Stv_vs_Cur	-0.775413478	-0.780178641	-4.270626106	3.212678308	0.50127831	0.857616425	8
Pseudomonas	Stv_vs_Cur	0.582420951	0.705534108	-2.91375371	4.811314196	0.562882586	0.857616425	7
Scatolibacter	Stv_vs_Cur	-0.302075606	-0.121667934	-3.510731166	4.152153891	0.530750659	0.857616425	5
Blautia	Stv_vs_Cur	0.60856337	0.50599381	-3.555291943	3.764326773	0.524629704	0.857616425	4
Robinsoniella	Stv_vs_Cur	-1.499722646	-1.692741527	-5.986905904	1.846657926	0.408376891	0.857616425	4
Vagococcus	Stv_vs_Cur	-0.302200075	-0.32443147	-3.452069009	3.055133842	0.562363961	0.857616425	4
Pusillibacter	Stv_vs_Cur	0.627259456	0.569692077	-3.011420869	4.981781271	0.451425507	0.857616425	3
Shigella	Stv_vs_Cur	0.758598891	0.892785636	-2.717310721	5.397508104	0.462882517	0.857616425	3

Supplementary Table S2. Genus-level raw count matrix used for ANCOM-BC analysis

Taxon	DSS_1	DSS_2	DSS_3	DSS_4	DSS_5	TSP_1	TSP_2	TSP_3	TSP_4	TSP_5	Cur_1	Cur_2	Cur_3	Cur_4	Cur_5	Stv_1	Stv_2	Stv_3	Stv_4	Stv_5
Turicibacter	17	3761	5048	5888	1174	5176	4991	3858	2576	4736	1337	2954	2521	3658	3836	3530	8268	3456	2465	3392
Kineothrix	614	6072	4228	11214	205	1872	815	3276	3909	4927	467	3643	4032	4190	5109	3992	1550	1012	6103	3510
Duncaniella	369	456	1580	1514	796	3164	3120	1964	1536	3386	2898	2043	3558	2554	4782	5087	4952	1239	1433	6963
Bacteroides	442	230	2281	592	2646	6169	3606	3560	1144	2134	6943	1862	804	4698	1826	2779	1649	1826	1677	920
Phocaeicola	439	248	2922	775	2790	1559	4324	2111	7188	886	5451	1357	134	3464	2731	1105	1039	6032	57	1080
Alistipes	128	1478	541	933	2081	1407	504	895	445	4329	4054	2371	7496	1006	2877	2123	557	5342	511	383
Escherichia	17299	812	793	7	9034	311	278	660	1881	61	16	15	17	3	500	464	639	9	421	1086
Faecalibaculum	7698	1990	104	480	3812	531	1436	1472	662	20	1632	1101	94	1265	575	29	184	842	295	52
Parabacteroides	214	80	326	436	5348	650	888	318	590	561	773	190	172	1152	383	857	567	1692	1345	131
Lawsonibacter	137	863	1344	288	1278	922	421	401	347	651	1244	754	1225	342	1402	1176	317	1168	755	1541
Ligilactobacillus	202	246	371	141	202	165	210	102	382	658	1092	6103	2722	316	468	429	1764	257	287	196
Muribaculum	350	195	1155	1998	286	559	549	736	388	87	338	1456	319	627	354	307	553	1592	1232	824
Akkermansia	148	61	146	2	46	2035	3338	2149	2737	12	13	5	12	230	128	45	15	137	84	117
Heminiphilus	335	179	1152	956	18	726	748	901	512	12	103	1837	39	767	333	178	73	13	1011	1360
Paraprevotella	2	0	73	13	38	1193	1797	2090	430	578	297	326	11	1583	427	889	10	228	62	49
Lachnoclostridium	55	724	905	414	67	623	684	317	962	764	242	237	670	285	761	654	52	40	269	771
Unclassified	74	356	447	500	80	299	202	234	603	398	250	583	915	801	377	739	136	695	800	851
Romboutsia	6	454	340	7	0	35	115	36	0	0	126	477	456	319	120	728	1672	438	1635	2126
Simiaoa	4	146	207	739	217	525	212	268	339	741	22	109	1477	158	875	959	147	4	161	965
Marseillibacter	147	1129	456	164	90	308	89	408	299	240	168	167	375	350	847	127	63	718	1256	119
Paramuribaculum	208	316	383	80	74	525	962	494	324	793	233	252	477	84	140	565	834	14	143	506
Lacrimispora	19	807	128	154	365	250	104	179	888	652	90	214	250	114	221	759	255	623	663	633
Mediterraneibacter	105	678	677	1176	30	244	338	302	360	539	155	295	400	266	231	229	187	93	286	369
Parasutterella	281	413	18	159	497	374	602	497	213	212	526	359	44	560	196	285	416	334	632	113
Butyricimonas	138	175	491	51	575	520	436	385	619	181	480	97	149	241	224	423	524	202	377	145
Acetatifactor	120	1856	797	21	23	299	46	110	147	501	26	45	400	0	113	279	117	30	168	357
Vallitalea	20	33	117	103	140	237	160	82	41	490	1237	711	205	281	182	122	126	231	190	698
Mucispirillum	271	761	207	603	150	122	88	297	37	239	63	113	46	229	110	188	24	764	1005	12
Clostridium	33	383	57	505	32	141	70	355	231	475	91	262	205	57	529	464	58	107	643	603
Bifidobacterium	1005	1359	19	83	346	105	343	1182	176	3	54	66	0	21	0	8	12	4	408	40
Vescimonas	205	609	695	390	38	245	48	299	255	208	50	112	216	192	373	427	32	94	342	324
Intestinimonas	107	542	416	264	144	195	117	193	177	315	157	182	323	239	293	316	42	206	416	354
Desulfovibrio	111	363	343	90	93	237	285	246	34	281	347	130	123	385	109	234	93	49	677	238
Flintibacter	131	655	450	271	77	191	92	200	187	235	77	108	253	162	258	222	31	78	484	220
Ruminiclostridium	38	10	130	262	174	107	380	84	42	245	585	624	220	419	146	300	125	147	70	175
Ruminococcus	2	0	0	17	0	118	214	113	166	310	187	442	111	391	134	253	582	479	21	716
Mammaliicoccus	12	10	21	63	0	12	16	14	77	305	20	33	26	25	7	13	3105	13	26	12
Anaerotignum	51	714	28	338	15	88	61	159	564	68	27	150	22	228	108	45	54	38	405	251
Sangeribacter	34	76	247	105	427	107	93	127	138	163	196	112	124	76	158	269	245	97	136	305
Neglectibacter	74	661	285	45	10	62	34	307	342	31	19	146	92	75	77	161	15	20	319	65

Butyrivibrio	0	90	15	213	0	72	0	653	20	0	95	0	965	0	11	0	178	281	0	0
Anaeromicropila	5	333	196	164	23	26	27	84	190	60	32	119	190	122	232	125	23	24	209	280
Christensenella	0	328	39	34	35	112	55	113	115	467	27	130	47	271	31	63	92	41	451	10
Barnesiella	0	6	14	44	187	0	0	0	167	156	141	0	18	15	133	10	109	1230	0	51
Oscillibacter	104	145	401	109	12	61	67	141	124	30	26	43	206	109	53	11	19	67	515	23
Guopingia	2	213	28	3	20	7	23	109	28	62	124	397	64	173	122	20	107	162	305	24
Staphylococcus	13	4	3	226	5	21	57	11	119	242	25	60	56	41	36	5	846	2	40	42
Limosilactobacillus	98	323	6	1	0	67	96	84	8	50	232	155	7	14	0	0	25	0	556	1
Catenibacillus	0	161	852	0	0	66	23	12	0	15	0	53	15	17	82	264	7	20	30	43
Dehalobacterium	16	149	67	173	7	30	33	41	47	90	31	41	182	65	31	60	30	84	181	77
Allobaculum	1109	52	73	91	2	0	0	0	0	0	0	0	0	0	0	0	0	0	0	0
Angelakisella	5	50	39	48	35	34	7	31	24	6	330	43	134	265	0	6	0	92	84	0
Pseudoflavonifractor	106	144	60	192	45	12	9	51	105	15	7	10	115	17	119	46	19	12	98	40
Acetivibrio	7	140	14	0	30	93	39	73	37	130	11	55	52	51	31	74	116	13	247	5
Butyricicoccus	21	96	73	78	6	25	13	57	27	13	32	63	105	24	208	22	21	29	207	36
Marasmitruncus	63	14	262	110	6	57	3	7	190	16	17	5	35	44	0	19	18	171	30	15
Feifania	11	31	53	14	48	27	81	25	70	54	54	22	41	43	80	244	20	32	24	27
Roseburia	0	142	177	64	0	145	62	61	20	58	5	74	38	0	0	80	28	0	40	0
Peptococcus	13	16	60	14	7	66	115	103	1	118	105	47	3	151	6	9	37	10	55	50
Vampirovibrio	0	10	18	16	39	31	60	25	17	120	124	88	74	39	25	47	77	47	44	15
Luoshenia	7	118	22	4	4	66	11	109	24	20	15	30	62	63	86	35	27	43	166	0
Muricomes	17	98	304	42	18	0	10	8	20	9	39	15	44	5	0	96	72	78	9	26
Aestuariispira	10	4	32	0	0	268	78	197	0	0	58	0	9	61	106	42	0	0	0	41
Qiania	23	49	233	70	0	14	16	48	96	30	6	12	21	55	24	11	0	41	58	96
Sporofaciens	12	128	208	20	0	7	0	47	71	48	9	45	0	0	23	99	18	0	42	95
Fusimonas	3	65	47	58	0	17	41	0	19	10	0	29	55	18	31	48	7	292	96	20
Bilophila	7	49	53	1	64	40	28	5	126	31	38	0	0	11	33	93	28	182	13	33
Petroclostridium	0	0	14	0	0	87	87	37	0	80	10	114	53	50	13	53	24	104	15	87
Harryflintia	28	184	29	30	7	29	11	60	20	21	1	32	42	20	40	36	0	27	172	27
Gracilibacter	0	52	7	1	44	18	11	23	3	10	169	61	28	18	61	19	10	181	94	0
Petrocella	7	68	8	0	0	21	3	31	30	3	0	79	91	175	0	49	0	0	240	0
Leptogranulimonas	221	41	3	6	180	32	70	64	31	0	22	12	0	17	0	0	0	10	0	0
Coprococcus	12	50	15	27	9	47	23	34	23	81	144	5	12	10	23	69	7	8	26	58
Anaerotruncus	7	45	51	90	0	7	0	69	22	15	2	4	70	27	5	44	20	43	82	40
Faecalimonas	0	51	17	43	7	5	19	34	13	48	28	62	61	54	8	70	9	23	81	6
Congzhengia	5	11	12	0	14	16	35	30	4	214	27	85	51	10	20	11	26	7	5	47
Eubacterium	8	3	32	5	6	40	85	65	9	28	2	7	30	53	34	96	48	32	0	4
Marvinbryantia	0	41	4	15	2	0	7	16	42	49	10	12	0	20	31	79	8	10	228	0
Odoribacter	1	0	2	20	14	3	6	0	2	50	29	0	23	37	50	59	44	226	0	0
Enterococcus	503	0	0	0	10	0	0	0	10	0	0	0	0	0	8	0	6	3	7	0
Rhodospirillum	4	0	18	36	49	0	0	0	50	3	150	8	19	24	0	0	49	70	19	45
Adlercreutzia	81	36	17	24	18	0	34	54	16	20	37	27	23	22	17	18	12	43	10	15
Anaerocolumna	5	87	42	38	1	35	0	69	47	0	0	0	26	8	0	22	17	43	71	12

Ruthenibacterium	28	32	22	30	3	13	7	12	88	10	5	11	12	78	35	27	0	5	9	96
Streptococcus	81	9	7	51	3	6	23	3	10	16	0	24	14	7	4	9	171	10	30	33
Dorea	10	29	15	0	8	32	15	11	85	32	12	20	29	15	8	20	0	4	52	99
Faecalicatena	0	0	307	0	5	63	0	8	19	0	8	14	0	0	25	8	0	12	0	10
Saccharofermentans	0	145	7	0	0	86	19	0	0	17	8	4	0	34	0	7	115	0	20	0
Waltera	0	15	97	81	0	5	8	5	9	11	0	24	21	0	39	33	1	0	42	50
Thomasclavelia	0	0	0	0	9	34	40	107	8	45	10	10	0	103	8	3	8	12	28	0
Jeotgalicoccus	0	0	0	4	0	0	1	3	0	19	0	5	3	0	5	0	371	0	10	0
Eisenbergiella	20	93	2	21	0	7	9	12	6	105	0	0	69	5	0	26	27	0	9	7
Aerococcus	0	0	0	0	0	0	0	0	0	0	0	0	0	0	0	0	380	0	0	0
Merdimmobilis	0	0	0	0	0	34	30	0	30	21	7	71	62	19	0	48	6	0	2	15
Anaeroplasma	0	92	5	0	4	0	0	31	0	24	0	23	26	22	0	11	2	0	87	0
Variimorphobacter	0	50	20	28	0	0	0	12	79	30	6	12	21	18	0	49	0	0	0	0
Natranaerovirga	0	52	5	7	0	0	13	21	6	44	0	42	27	3	21	21	15	0	31	5
Amedibacillus	10	11	74	11	32	0	1	6	6	5	11	0	22	14	22	6	12	29	2	24
Fumia	4	72	48	24	7	1	12	4	13	8	3	0	30	4	18	7	0	4	17	21
Lentihominibacter	0	5	13	13	0	13	13	12	13	35	16	21	29	7	20	31	2	10	8	20
Corynebacterium	2	30	28	55	0	0	0	0	33	12	0	0	25	8	25	11	11	5	4	20
Dysosmobacter	3	47	23	27	0	0	4	17	7	28	8	5	12	16	13	37	0	0	20	0
Anaerobium	0	56	57	42	0	5	0	5	15	12	2	0	4	10	9	14	6	0	9	9
Lactobacillus	4	17	30	0	0	1	0	43	0	0	0	0	98	0	29	0	25	0	0	0
Sinanaerobacter	4	29	21	25	0	9	0	7	21	11	1	6	13	11	18	23	0	5	14	17
Sporobacter	0	21	15	22	4	8	5	6	0	22	14	6	14	2	3	36	4	14	31	2
Ileibacterium	171	16	24	17	0	0	0	0	0	0	0	0	0	0	0	0	0	0	0	0
Ihubacter	0	5	12	0	11	4	31	12	5	10	20	5	0	10	7	4	3	33	8	12
Enterocloster	0	23	17	22	5	5	6	9	13	0	5	6	0	0	0	20	4	6	33	12
Abyssivirga	0	1	12	3	3	10	25	12	13	5	32	4	5	0	8	28	9	0	6	0
Acutalibacter	0	42	0	0	0	0	0	0	23	24	0	0	43	10	24	0	0	0	10	0
Xiamenia	8	22	6	2	4	6	11	8	0	8	4	5	3	8	27	34	8	0	0	10
Holdemania	1	3	8	5	0	4	10	5	3	13	0	9	5	25	15	4	3	11	6	18
Zhenpiania	12	8	14	0	11	5	0	8	14	4	6	3	4	1	8	17	3	5	3	16
Paludihabitans	0	15	31	13	6	0	0	1	8	0	0	0	10	0	1	12	0	0	17	13
Jingyaoa	0	8	13	19	0	9	0	6	7	0	0	0	13	0	0	3	0	0	46	0
Evtepia	0	0	0	0	0	15	0	0	0	0	0	2	0	0	0	0	0	81	0	21
Tannockella	0	0	0	0	2	0	4	8	0	0	5	0	1	0	0	3	0	56	14	23
Mageeibacillus	0	6	10	0	19	21	5	4	0	0	4	0	0	4	4	5	0	0	12	8
Berryella	0	5	0	5	0	0	4	10	9	6	4	3	8	3	0	7	4	7	15	5
Oxalobacter	0	0	3	0	3	4	9	6	0	9	10	3	6	8	3	10	3	12	5	0
Rhizobium	0	0	2	4	22	0	0	0	0	0	14	2	0	3	0	18	4	19	0	4
Anaerobacterium	0	0	0	4	0	0	8	0	0	1	0	40	21	10	0	0	0	5	0	0
Breznakia	0	4	0	0	0	0	0	4	0	9	7	51	8	0	0	0	2	0	0	0
Raoultibacter	3	3	6	3	0	6	6	6	5	9	3	6	4	2	6	2	6	3	5	0
Frisingicoccus	0	16	12	13	0	0	0	0	0	16	0	0	0	0	0	16	0	3	4	0

Gordonibacter	0	5	5	3	6	2	0	7	0	6	7	9	6	8	7	0	0	0	0	5
Lachnotalea	0	2	0	14	0	5	0	0	0	4	0	0	6	19	0	5	0	8	4	0
Hungatella	0	2	0	0	0	2	0	2	16	0	0	0	5	0	0	0	0	0	29	0
Rhabdanaerobium	0	0	0	0	0	0	0	0	0	0	0	0	55	0	0	0	0	0	0	0
Ructibacterium	0	0	0	0	0	6	38	11	0	0	0	0	0	0	0	0	0	0	0	0
Diplocloster	0	0	0	0	0	0	6	17	0	0	0	0	0	0	0	0	0	0	17	0
Wansuia	0	0	0	0	0	0	0	0	39	0	0	0	0	0	0	0	0	0	0	0
Agathobaculum	0	0	0	0	0	0	0	0	0	0	0	0	0	0	0	0	0	0	32	0
Intestinibacillus	0	17	2	3	0	0	0	4	0	0	0	0	4	0	0	0	0	0	0	0
Alkalibaculum	0	0	0	0	6	0	2	0	0	0	3	0	0	0	2	9	0	0	3	0
Longibaculum	0	0	4	0	0	0	0	2	0	0	0	4	0	7	0	0	0	8	0	0
Monoglobus	0	4	0	3	0	0	5	0	0	2	0	6	0	0	0	2	0	0	2	0
Cuneatibacter	0	0	0	0	0	0	0	0	0	4	0	0	0	0	0	0	10	0	7	0
Solibaculum	0	0	4	0	0	6	0	6	0	0	0	0	0	5	0	0	0	0	0	0
Cupriavidus	0	0	0	0	0	0	0	0	0	0	0	3	0	0	0	0	15	2	0	0
Gehongia	2	0	0	0	0	0	0	0	5	0	4	0	0	0	0	0	0	7	0	0
Jutongia	0	0	0	0	0	0	0	0	0	10	0	0	0	0	0	6	0	0	0	1
Bittarella	0	0	4	2	0	0	7	0	0	0	3	0	0	0	0	0	0	0	0	0
Massiliimalia	0	0	0	3	0	0	0	0	0	0	0	0	0	0	0	0	0	13	0	0
Enteroscipio	3	4	0	3	0	0	0	0	0	0	0	0	0	0	0	0	5	0	0	0
Caloramator	0	0	0	0	0	0	0	0	0	0	0	0	0	0	0	0	0	12	0	0
Hydrogeniiclostridium	0	0	0	0	0	0	3	7	0	0	0	0	2	0	0	0	0	0	0	0
Arabiibacter	0	0	0	0	0	0	0	0	0	0	0	0	0	0	0	0	0	0	7	4
Aminipila	1	2	0	0	0	0	0	0	0	0	0	0	0	0	0	0	0	0	6	0
Victivallis	0	0	0	0	0	5	0	0	0	0	4	0	0	0	0	0	0	0	0	0
Atopostipes	0	0	0	0	0	0	0	0	0	0	0	0	0	0	0	0	8	0	0	0
Massilioclostridium	0	0	0	0	0	0	0	0	0	0	0	0	2	2	0	0	0	0	4	0
Sutterella	0	0	0	0	0	0	0	0	0	0	0	0	0	8	0	0	0	0	0	0
Hominisplanchenecus	0	0	7	0	0	0	0	0	0	0	0	0	0	0	0	0	0	0	0	0
Intestinibacter	0	0	0	0	0	0	0	0	0	0	0	0	0	0	0	0	7	0	0	0
Pseudomonas	0	0	0	0	0	0	2	0	0	0	0	0	0	0	0	0	5	0	0	0
Anaerosacchariphilus	0	0	6	0	0	0	0	0	0	0	0	0	0	0	0	0	0	0	0	0
Scatolibacter	0	0	0	0	0	2	0	3	0	0	0	0	0	0	0	0	0	0	0	0
Anaeromassilibacillus	0	0	0	0	0	0	0	0	4	0	0	0	0	0	0	0	0	0	0	0
Blautia	0	0	0	0	2	0	0	0	0	0	0	0	0	0	0	0	2	0	0	0
Robinsoniella	0	0	0	0	0	0	0	0	0	0	0	1	0	3	0	0	0	0	0	0
Vagococcus	0	0	0	0	0	0	0	0	0	0	0	0	0	2	0	0	0	0	2	0
Anaerostipes	0	0	0	0	0	0	0	0	0	0	0	0	3	0	0	0	0	0	0	0
Pusillibacter	0	0	0	0	0	1	0	0	0	0	0	0	0	0	0	0	0	0	2	0
Serpentinicella	0	0	0	0	0	0	3	0	0	0	0	0	0	0	0	0	0	0	0	0
Shigella	1	0	0	0	0	0	0	0	0	0	0	0	0	0	0	0	0	0	2	0
Anaerotaenia	0	0	0	0	0	0	0	0	0	0	0	0	0	0	0	2	0	0	0	0
Bacillus	0	0	0	0	0	0	0	0	0	0	0	0	0	0	0	0	2	0	0	0

Caldalkalibacillus	0	0	0	0	0	0	0	2	0	0	0	0	0	0	0	0	0	0	0	0
Kiloniella	0	0	0	0	0	0	0	0	0	0	0	0	0	0	0	0	0	0	0	2
Peptacetobacter	0	0	0	0	0	0	0	0	0	0	0	0	0	0	0	0	0	0	0	2
Actinomarinicola	0	0	1	0	0	0	0	0	0	0	0	0	0	0	0	0	0	0	0	0
Anaerosporobacter	0	1	0	0	0	0	0	0	0	0	0	0	0	0	0	0	0	0	0	0
Brassicibacter	0	0	0	0	0	0	0	0	0	1	0	0	0	0	0	0	0	0	0	0
Desulfosporosinus	0	0	0	0	0	0	0	0	0	0	1	0	0	0	0	0	0	0	0	0
Seongchinamella	0	0	0	0	0	0	0	0	0	0	0	0	0	0	0	0	0	0	1	0
Solirubrobacter	0	0	0	0	0	0	0	0	0	0	0	0	0	1	0	0	0	0	0	0
Sphaerobacter	0	0	0	0	0	0	1	0	0	0	0	0	0	0	0	0	0	0	0	0
Abiotrophia	0	0	0	0	0	0	0	0	0	0	0	0	0	0	0	0	0	0	0	0
Absiella	0	0	0	0	0	0	0	0	0	0	0	0	0	0	0	0	0	0	0	0
Anaerofustis	0	0	0	0	0	0	0	0	0	0	0	0	0	0	0	0	0	0	0	0
Culicoidibacter	0	0	0	0	0	0	0	0	0	0	0	0	0	0	0	0	0	0	0	0
Emergencia	0	0	0	0	0	0	0	0	0	0	0	0	0	0	0	0	0	0	0	0
Falcatimonas	0	0	0	0	0	0	0	0	0	0	0	0	0	0	0	0	0	0	0	0
Merdimonas	0	0	0	0	0	0	0	0	0	0	0	0	0	0	0	0	0	0	0	0
Murimonas	0	0	0	0	0	0	0	0	0	0	0	0	0	0	0	0	0	0	0	0
Sporosarcina	0	0	0	0	0	0	0	0	0	0	0	0	0	0	0	0	0	0	0	0
Tepidibaculum	0	0	0	0	0	0	0	0	0	0	0	0	0	0	0	0	0	0	0	0
Tyzzerella	0	0	0	0	0	0	0	0	0	0	0	0	0	0	0	0	0	0	0	0
